# The complex DNA molecular combination with a linear and circular structure in *Magnolia kwangsiensis* mitochondrial genome

**DOI:** 10.3389/fpls.2025.1590173

**Published:** 2025-05-29

**Authors:** Qing Li, Wentao Sheng

**Affiliations:** ^1^ Department of Biological Technology, Nanchang Normal University, Nanchang, Jiangxi, China; ^2^ Jiangxi Provincial Key Laboratory of Poultry Genetic Improvement, Nanchang Normal University, Nanchang, China

**Keywords:** *M. kwangsiensis*, mitochondrial genome, branched structure, repeat, RSCU

## Abstract

**Background:**

*Magnolia kwangsiensis* is an endangered Magnoliaceae species, holding scientific, aesthetic, and economic value. But limited genetic research was reported. To better understand *M. kwangsiensis* genomics, we aimed to assemble and analyze its mitochondrial genome.

**Results:**

*M. kwangsiensis* has a branched structure, composing a linear and circular molecular structure with 428,449 bp and 126,869 bp, and GC contents of 47.51% and 47.38%. The total genome size is 555,318 bp, with GC content of 47.47%. A total of 68 genes were annotated, comprising 40 protein-coding, 23 tRNA, three rRNA genes, and two pseudo-genes. And *nad1*, *atp9*, and *nad6* exhibited the highest nucleotide diversity, while *atp1* and *nad5* exhibited the lowest. Relative synonymous codon usage (RSCU) analysis revealed 29 codons with RSCU values > 1, showing A/U preference for codons ending. Moreover, 211 simple sequence, 1101 dispersed, and 39 tandem repeats were checked. The mitochondrial genome of *M. kwangsiensis* and *Magnolia figo* showed relatively good synteny. And 32 homologous fragments were discriminated between its mitochondrial and chloroplast genome, with a total of 29,253 bp and an average 914 bp. Phylogenetic analysis indicated that *M. kwangsiensis* is the most closely related to *Liriodendron tulipifera*.

**Conclusion:**

The information provided herein contributes genomic knowledge for the *M. kwangsiensis* breeding research.

## 1 Introduction

Mitochondria are the main sites of respiration and the primary cellular energy supply organelles in plants, producing adenosine triphosphate through oxidative phosphorylation, providing energy for life metabolic activities (Martin et al., 2016). Mitochondria are semi-autonomous organelles, playing an important role in the vitamins, amino acids, and fatty acids synthesis (Vothknecht and Szabo, 2020). There are many mitochondria in a cell, each with dozens to hundreds of copies of the genome, so there are multiple mitochondrial genomes in a cell. Most plant mitochondrial genomes are composed of circular DNA molecules and linear DNA molecules of different sizes and proportions (Gualberto and Newton, 2017). The mitochondrial genomes of terrestrial plants vary in size (208 kb~11 Mb), much larger than those of animals (15~17 kb) (Sloan, 2013; Gualberto and Newton, 2017). Due to its complex characteristics, the mitochondrial genomes obtained from plants are far less than those obtained from plant chloroplast genomes and animal mitochondrial genomes, especially the presence of highly enriched repetitive sequences and frequent recombination, which hinder the progress of precise assembly (Wu et al., 2022). Due to the fact that Illumina’s reading length usually does not span different segments, it is necessary to assemble different segments, which cannot accurately determine the length and content of mitochondrial genomes. However, third-generation long-read sequencing such as Oxford Nanopore and PacBio sequencing improves coverage and allows for construction in previously unassembled genomic regions (Bi et al., 2024).

As of February 25, 2025, over 10,003 plant mitochondrial genome sequences have been recorded in NCBI (https://www.ncbi.nlm.nih.gov/nuccore). Currently, relevant research has been conducted in mitochondrial genomics, Feng et al. (2023) utilized second and third-generation sequencing techniques to assemble the genus *Gossypium* mitochondrial genome, identifying that repeat sequences and foreign sequences are significant contributors to the genomic size variations in this genus. Mitochondrial genome comparative analysis of cultivated and wild lettuce revealed a high A/T base content in their coding genes and weak codon preference (Zhang et al., 2022c). The *Capsicum annuum* mitochondrial genome of the cytoplasmic male-sterile line HNUCA00HG was sized at 564,124 bp, annotating 77 known functional genes. Comparison with two other pepper sterile lines indicated that the three sterile lines likely share a common ancestor, though significant differences exist (Li et al., 2022). And SSR markers developed from the *Citrullus lanatus* mitochondrial genome were used for genetic diversity analysis, revealing that the rough-netted thick-skinned melon type formed a distinct branch, while other branches were related to variety origins (Zhang et al., 2020a). Furthermore, Zhang’s research (Zhang et al., 2024a) on the genus *Crataegus* mitochondrial genome revealed that *Crataegus maximowiczii* var. *ninganensis*, *C. maximowiczii*, and *C. bretschneideri* are closely related, providing foundational data for the identification and systematic classification of this genus. It is thus clear that reported research on plant mitochondrial genome primarily focuses on genome size, gene annotation, codon preference, dispersed repeat sequences, nucleotide polymorphism, mitochondrial structure comparisons (Ye et al., 2022; Wang et al., 2024b; Li et al., 2025), comparisons of mitochondrial and chloroplast homologous sequences (Qu et al., 2022), cytoplasmic male sterility (Wang et al., 2020), genetic diversity (Zubaer et al., 2018), and phylogenetic analysis (Sun et al., 2022; Li et al., 2023). From this, it can be seen that the mitochondrial genome is an excellent tool for plant genetic research.

*Magnolia kwangsiensis* Figlar & Noot. is an evergreen broadleaf tree in the Magnoliaceae family, growing in limestone mountainous forests at altitudes of 300 to 500 m. It is a first-class protected plant in China and is recognized as an extremely endangered species by the International Union for Conservation of Nature (Schmidt et al., 2023). This group of plants primarily distributes in southern Guangxi and Xishuangbanna in Yunnan, with sporadic distributions in Luocheng and Huanjiang county in northern Guangxi and Libo county in southeastern Guizhou, making it a precious and rare ornamental tree (Dong et al., 2009). And *M. kwangsiensis* is a monotypic species of the genus *Magnolia* in Magnoliaceae, and it is the only species in the primitive group of Magnoliaceae that is dioecious and has unisexual flowers, which is extremely rare among primitive Magnoliaceae plants, representing a relatively advanced group within primitive Magnoliaceae (Li and Guo, 2014). This plant is the only dioecious species in Magnoliaceae with a facultative apomictic reproductive system, making it of great research value for studying the evolutionary classification system of Magnoliaceae and the species sex differentiation (Shi et al., 2024). And research on *M. kwangsiensis* is scarce, mainly focusing on species identification, morphological structure, disease research, and vegetation diversity surveys (Lin et al., 2008; Wang et al., 2023; Guo et al., 2020). Tan et al. conducted pollen paraffin sectioning and cytological compression observations on *M. kwangsiensis*, concluding that the cytoplasmic division mode during microspore formation is simultaneous and modified, with tetrahedral arrangements being predominant. And the degeneration in male gametophyte development is a major factor affecting the endangerment of *M. kwangsiensis* (Tan et al., 2009).

In terms of genomic research on *M. kwangsiensis*, Zhao analyzed the reasons for its endangerment from sparse and dense population genetic structures (Zhao et al., 2010). Lin et al. (2010) and Xu et al. (2018) developed microsatellite repeat sequences (SSRs) from transcriptome sequences of different tissues for genetic research on this species. Kuang et al. (2011) reported the *M. kwangsiensis* chloroplast genome in 2011, which is a circular double-stranded structure with 159,667 bp, containing 129 genes, 17 of which are duplicated in the IR region. In 2024, Shi et al. reported its nuclear genome sequence, which is 2.67G in size, with a genome coverage of 98% and a total of 35,927 annotated genes, with repetitive sequence lengths accounting for 64.87% of the entire genome sequence (Shi et al., 2024). Up to now, 59 nucleotide sequences of *M. kwangsiensis* are retrieved from NCBI nucleotide database (https://www.ncbi.nlm.nih.gov/nuccore), while no research reports on the *M. kwangsiensis* mitochondrial genome have been found. Therefore, the study of mitochondrial genome will promote the research on the sex mechanism of *M. kwangsiensis*.

Currently, plant mitogenomes can be assembled either based on mitochondrial DNA (mtDNA) sequencing, which requires mtDNA isolation and purification, or directly from whole-genome sequencing (WGS) data (Bi et al., 2024). This study will assemble the *M. kwangsiensis* mitochondrial genome using the original WGS data already registered in NCBI to obtain its complete sequence. The main aims of this study were to: (1) by verifying the reported genome sequencing data, the mitochondrial genome of this species can be assembled; (2) supply newly sequenced complete mitochondrial genomes for the genus *Magnolia* and understand their overall genome structures; (3) compare these six complete mitochondrial genomes and identify highly divergent regions for the Magnoliaceae family; (4) construct phylogeny in the genus *Magnolia* and Magnoliaceae family using protein-coding gene (PCG) sequences of mitochondrial genomes.

## 2 Methods

### 2.1 Data sources

Raw sequencing data for Illumina and PacBio platform of *M. kwangsiensis* genome were downloaded from NCBI under project PRJNA1057110 (https://www.ncbi.nlm.nih.gov/bioproject/1057110). The final dataset utilized for subsequent analyses in this study comprised Illumina, PacBio HiFi, and HIC data, with a total volume of 138.60 Gb, 473.45 Gb, and 140.81 Gb (Shi et al., 2024).

### 2.2 The *M. kwangsiensis* mitochondrial genome assembly and annotation

The CANU v2.3 (https://github.com/marbl/canu/releases) and PMAT (v1.5.3,-g5G) (https://github.com/bichangwei/PMAT) were made to assemble the contig sequences from the *M. kwangsiensis* sequencing data, and BLAST (https://blast.st-va.ncbi.nlm.nih.gov/) was utilized to align counting to the published plant mitochondrial genomes. The contigs that aligned were used as seed sequences, and the original data were utilized to circularize the sequences. NextPolish2 (https://github.com/Nextomics/NextPolish2) was used to correct the results, and manual correction was conducted to gain the final assembly results. BLAST was employed for the *M. kwangsiensis* mitochondrial gene annotation; tRNAscan-SE 2.0 (http://lowelab.ucsc.edu/tRNAscan-SE/) was utilized for tRNA annotation; and OGDRAW 1.3.1 (https://chlorobox.mpimp-golm.mpg.de/OGDraw.html) was made to draw the *M. kwangsiensis* mitochondrial genome map. A custom Perl script was used to filter the coding sequence (CDs) regions of the *M. kwangsiensis* mitochondrial genes and calculate its codon preference.

### 2.3 Nucleotide diversity Pi value in the *M. kwangsiensis* mitochondrial genome

Homologous gene sequences were aligned with MAFFT version 7 (https://mafft.cbrc.jp/alignment/server/), and Dnasp6 (Rozas et al., 2017) was employed to calculate the Pi values and produce visual representation for each gene.

### 2.4 *M. kwangsiensis* mitochondrial genome repetitive sequences

Reputer software (https://bibiserv.cebitec.uni-bielefeld.de/reputer) (Stefan et al., 2001) was utilized to detect dispersed repeats in the *M. kwangsiensis* mitochondrial genome, with a minimum 30 bp repeat length. MISA software (Beier et al., 2017) was made to seek SSRs in the *M. kwangsiensis* mitochondrial genome, with a minimum repeat value of 10 (mononucleotides), 5 (dinucleotides), and 4 (trinucleotides), while the minimum repeat values for tetranucleotides, pentanucleotides, and hexanucleotides were set to 3.

### 2.5 Homology and synteny analysis of the *M. kwangsiensis* mitochondrial sequences

Mauve2.1.0 (https://darlinglab.org/mauve/user-guide/viewer.html) was used to compare the *M. kwangsiensis* genomes with *Liriodendron tulipifera* (MK340747.1), *Magnolia liliiflora* (NC_085212.1), *Magnolia officinalis* (NC_064401.1), *Magnolia figo* (NC_082234.1), and *Magnolia biondii* (NC_049134.1), and to analyze their synteny.

### 2.6 Homologous sequence comparison in the *M. kwangsiensis* organelle genome

BLAST software (https://blast.st-va.ncbi.nlm.nih.gov/) was used with a similarity threshold of 70% and an E-value of 1×10^–5^ to find homologous genome sequences between *M. kwangsiensis* mitochondrion and chloroplast, and circos-0.69-9 (http://circos.ca/software/download/) was utilized for visualization.

### 2.7 Phylogenetic analysis

Mitochondrial genomes from 29 species, including six Magnoliaceae, nine Lauraceae, one Saururaceae, three Brassicaceae, one Caricaceae, four Poaceae, two Schisandraceae, one Nymphaeaceae, and one Amborellaceae plant were downloaded from NCBI (https://www.ncbi.nlm.nih.gov/), using the gymnosperm *Ginkgo biloba* as an out-group. PhyloSuite (V1.2.1) (Zhang et al., 2020b) was made to identify and extract the 23 conserved genes (*nad1*, *nad3*, *nad2*, *nad4*, *nad5*, *nad4L*, *nad6*, *nad9*, *nad7*, *ccmB*, *ccmC*, *cob*, *cox1*, *cox3*, *cox2*, *atp1*, *atp6*, *atp4*, *atp8*, *rps3*, *atp9*, *rps4*, and *rps12*) from each species. MAFFT (V7.450) (Katoh et al., 2019) was utilized to align the conserved gene sequences, and the aligned sequences were concatenated to build a phylogenetic tree. The best model was constructed using ModelFind, and maximum likelihood (ML) analysis was conducted in RaxML (V8.2.4) (HÖhler et al., 2022) with 1000 bootstrap replicates.

## 3 Results

### 3.1 *M. kwangsiensis* mitochondrial genome

**Figure 1:**
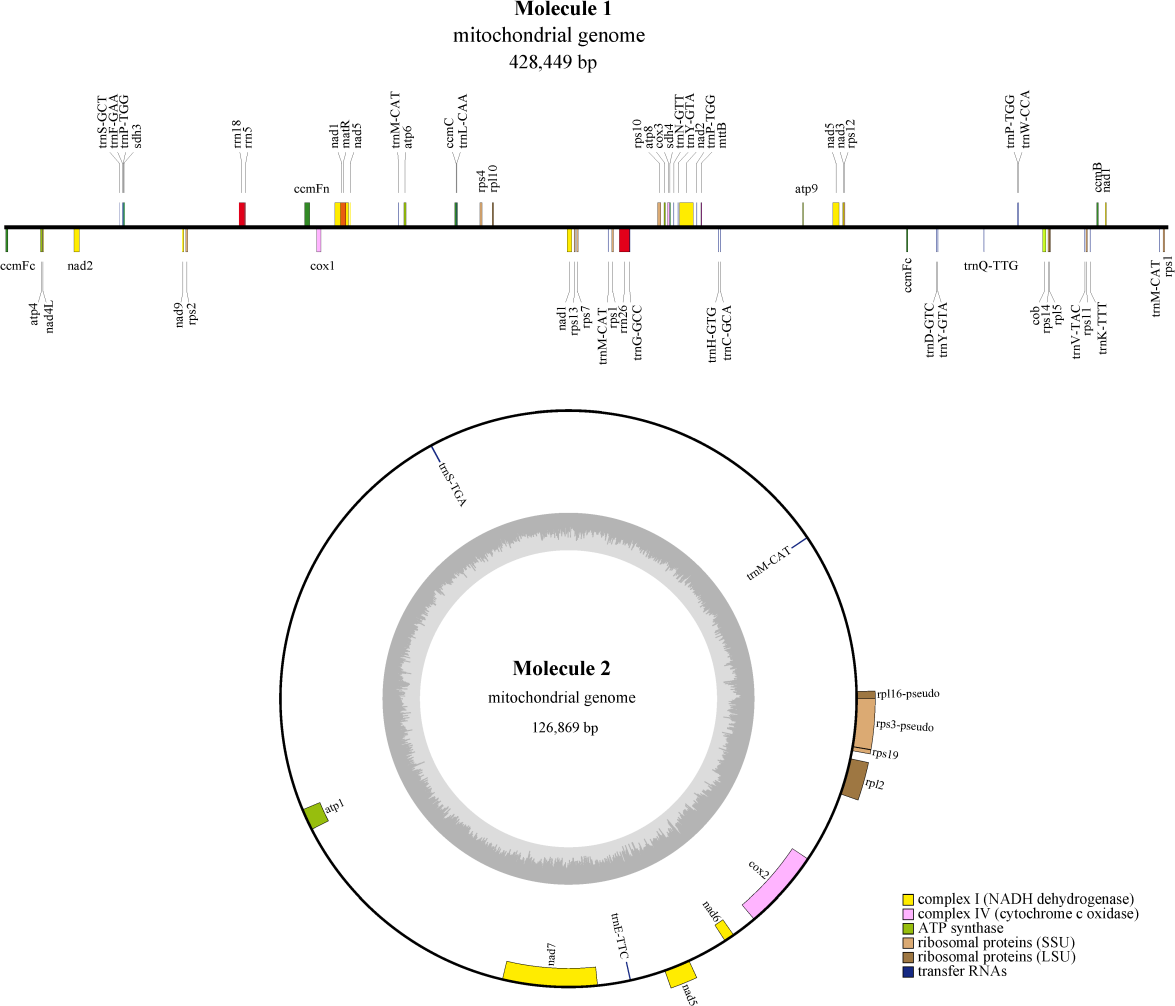
Mitochondrial genome map of *Magnolia kwangsiensis.* Note: Mitochondrial genome molecular 1 map in *Magnolia kwangsiensis*; Mitochondrial genome molecular 2 map in *Magnolia kwangsiensis* (Circle outside genes: Forward coding of genes; circle inside genes: Reverse coding of genes; Internal dark gray circle: GC content).

This study statistically analyzed the raw sequencing data of *M. kwangsiensis* and found that the entire mitochondrial coverage was uniform (**Supplementary Figure S1**), with a minimum sequencing depth of 36 times, a maximum sequencing depth of 903 times, and an average sequencing depth of 253.3 times (**Supplementary Figure S2**). The *M. kwangsiensis* mitochondrial genome was assembled and its complete structure was obtained (**Figure 1**; **Supplementary Figure S3** gfa plot), which consists of two parts: one part is a circular complex structure that includes repeat sequences, while the other part is a free circular structure. After unraveling the gfa plot, we obtained a combination of a linear structure and a circular DNA molecule, with 428,449 bp and 126,869 bp size, respectively, and NCBI accession numbers PQ822236 and PQ822237, with GC contents of 47.51% and 47.38% (**Figure 1**; **Table 1**). The total *M. kwangsiensis* mitochondrial genome size is 555,318 bp, with A content of 27.51%, T content of 27.24%, C content of 22.94%, G content of 22.31%, and GC content of 47.47%. A total of 68 genes were annotated, containing 40 PCGs, 23 tRNA, 3 rRNA, and 2 pseudo-genes (*rpl16* and *rps3*) (**Figure 1**; **Supplementary Table S1**).

**Table 1:** The basic information of *Magnolia kwangsiensis* mitochondrial genome.

Genome	Genome_type	Genome_length	Genome_GC(%)	CDS_nu	CDS_len	CDS_GC(%)	tRNA_nu	tRNA_len	tRNA_GC(%)	rRNA_nu	rRNA_len	rRNA_GC(%)	Pseudo_gene_nu
*Magnolia_kwangsiensis*-molecule 1	linear	428,449	47.51	34	26,107	44.17	20	1498	50.87	3	5876	52.98	0
*Magnolia_kwangsiensis*-molecule 2	circular	12,6869	47.38	6	5,987	45.08	3	233	48.07	0	0	0	2
*Magnolia_kwangsiensis*_Total	–	555,318	47.47	40	32,094	44.34	23	1731	50.49	3	5876	52.98	2

len, length; nu, number.

Furthermore, there are nine genes (*nad1*, *nad3*, *nad2*, *nad4*, *nad5*, *nad4L*, *nad7, nad6*, and *nad9*) for Complex I (NADH dehydrogenase); one gene (*cob*) for Complex III; three genes (*cox1*, *cox3*, and *cox2*) for Complex IV; five genes (*atp1*, *atp6*, *atp4*, *atp9*, and *atp8*) for ATP synthase; three genes (*rrn5*, *rrn18*, and *rrn26*) for ribosomal RNA; and 17 genes (*trnC-GCA*, *trnE-TTC*, *trnD-GTC*, *trnF-GAA*, *trnG-GCC*, *trnK-TTT*, *trnH-GTG*, *trnL-CAA*, *trnM-CAT*, *trnP-TGG*, *trnN-GTT*, *trnQ-TTG*, *trnS-TGA*, *trnS-GCT*, *trnW-CCA*, *trnV-TAC*, and *trnY-GTA*) for tRNA. These genes (*ccmB*, *ccmFc*, *ccmC*, and *ccmFn*) are involved in regulating the biosynthesis of cytochrome c; additionally, genes such as *rpl2*, *rpl5*, *rpl10*, *rpl16*, *rps1*, *rps11*, *rps10*, *rps12*, *rps14*, *rps13*, *rps2*, *rps19*, *rps4*, and *rps7* are used to encode ribosomal proteins. Other genes, such as *matR*, can encode maturation enzymes, and *mttB* can encode transporters, while *sdh3* and *sdh4* encode succinate dehydrogenase. The *M. kwangsiensis* mitochondrial genome contains nine genes with introns, where *ccmFc*, *rpl2*, and *rps10* each contain one intron, *cox2* contains two introns, *nad4* contains three introns, and *nad1*, *nad5*, *nad2*, and *nad7* each contain four introns. The *trnY-GTA*, *trnP-TGG*, and *trnM-CAT* genes contain two, three, and four copies, respectively (**Supplementary Table S1**). The total 40 PCGs length in the *M. kwangsiensis* mitochondrial genome is 32,094 bp, accounting for 5.78%. In addition, *rrn26* is the longest at 3669 bp, *rrn18* is the second longest at 2086 bp. However, *rrn5* is the shortest at 121 bp, and *atp9* is the second shortest at 225 bp (**Supplementary Table S2**).

### 3.2 Codon preference of the *M. kwangsiensis* mitochondrial genome

**Figure 2:**
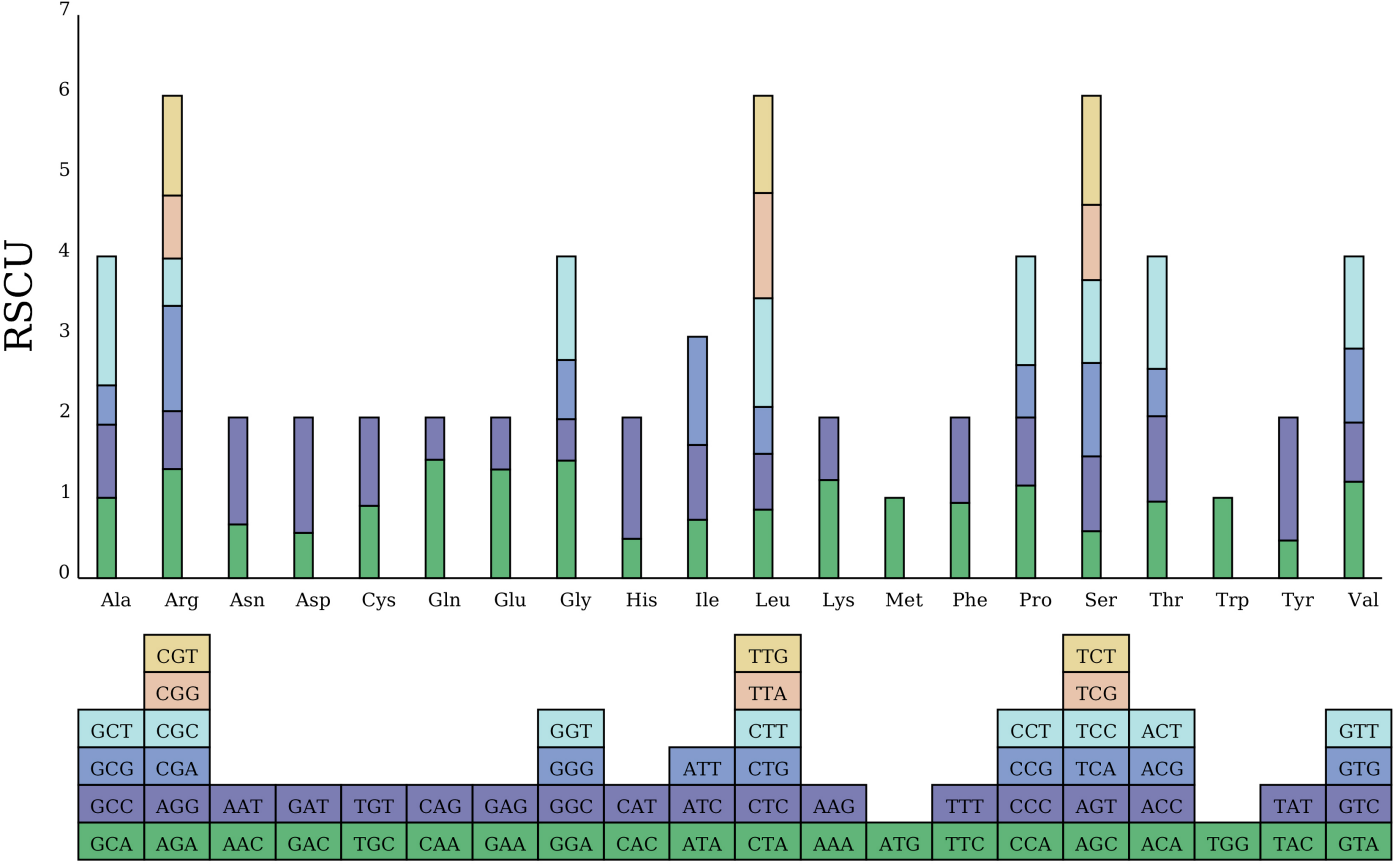
Statistical analysis of *Magnolia kwangsiensis* mitochondrial codon preference.

The codon preference analysis indicates that 31 codons with relative synonymous codon usage (RSCU) values are > 1, of which 28 end with A/U bases; there are two codons with RSCU = 1, which are tryptophan (Trp) and methionine (Met) amino acids, both ending with U; and there are 31 codons with RSCU < 1, of which four end with A/U bases (**Figure 2**), indicating a A/U preference for codons ending in the *M. kwangsiensis* mitochondrial genome. The 68 genes contain a total of 10,496 codons, with leucine (Leu) being the most frequently used codon, totaling 1012, accounting for 9.64%; cysteine (Cys) is the least used, totaling 140, accounting for 1.33%. The codons for Ala (GCU) and Ter (UAA) are the most frequently used, with RSCU values of 1.6047 and 1.5789, showing significant preference; the codons for tryptophan (UGG) and methionine (AUG) show no preference in the *M. kwangsiensis* mitochondrial genome (RSCU = 1); the stop codon UAG and the codon for Tyr (UAC) have RSCU values of 0.3158 and 0.4674, indicating the least usage (**Supplementary Table S3**).

### 3.3 Pi value of the *M. kwangsiensis* mitochondrial genome

**Figure 3:**
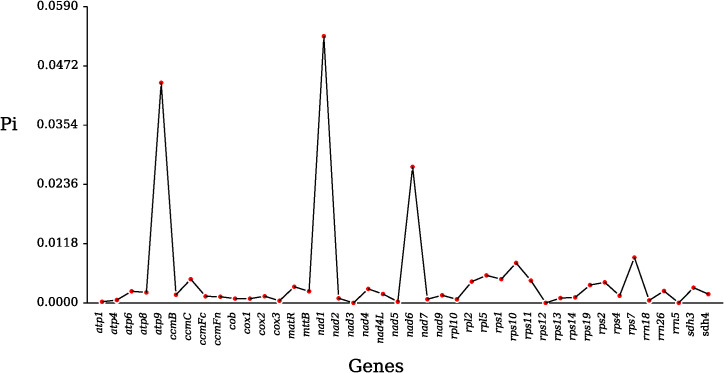
Line graph of gene Pi values in *Magnolia kwangsiensis*.

The Pi values of *nad1*, *atp9*, and *nad6* are 0.05312, 0.04385, and 0.0271, respectively, demonstrating the highest nucleotide diversity; the Pi values of *nad3*, *rps12*, and *rrn5* are zero, indicating no nucleotide diversity; the Pi values of *atp1* and *nad5* are 0.00023 and 0.00024, respectively, indicating the lowest nucleotide diversity (**Figure 3**; **Supplementary Table S4**). The genes with a high number of variant sites are *nad1*, *nad6*, and *atp9*, with 153, 57, and 29. The genes *nad4*, *rpl2*, *rrn26*, and *matR* have moderate variant sites, with 10, 12, 13, and 18. There are 32 genes with variant site counts between 1 and 10, among which *atp1*, *atp4*, *cox3*, *nad4L*, *nad5*, *rpl10*, *rps13*, and *rps14* have one variant site each; *mttB*, *nad7*, *nad9*, *rrn18*, and *sdh4* have two variant sites each; and *atp8*, *atp6*, *ccmFc*, *ccmB*, *cox2*, *cob*, *rps19*, *rps4*, and *sdh3* have three variant sites each (**Supplementary Table S4**).

### 3.4 Repeat sequences

**Figure 4:**
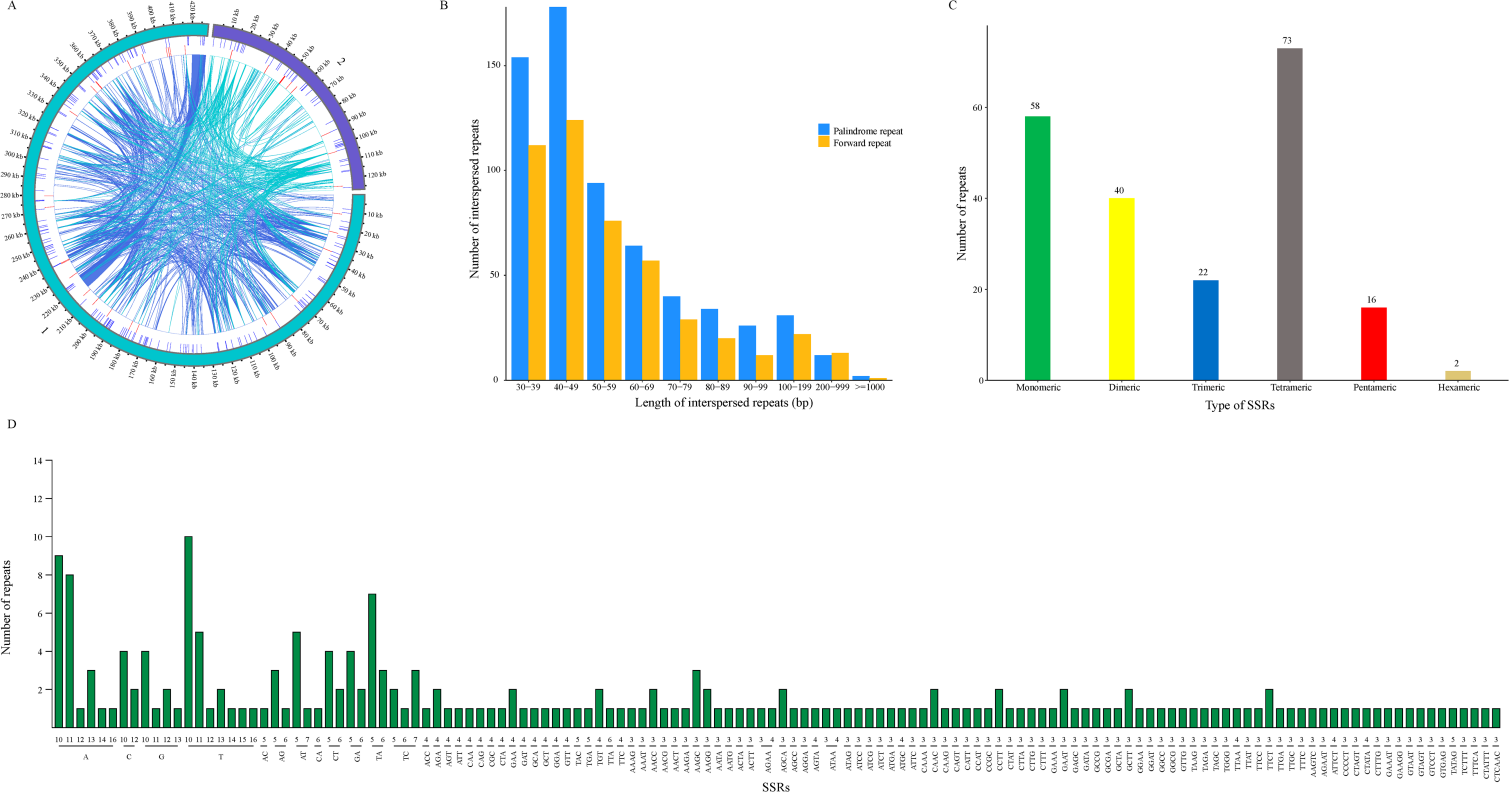
Repeat sequences of the *Magnolia kwangsiensis* mitochondrial genome. **(A)** The distribution of repetitive sequences; **(B)** SSR distribution; **(C)** dispersed repeat length distribution; **(D)** SSR type composition.

A total of 211 SSRs, 1101 dispersed repeats, and 39 long tandem repeat sequences (LTRs) were discriminated in the *M. kwangsiensis* mitochondrial genome (**Figure 4A**). Among the 211 SSRs, four-base repeats were the most abundant (73), followed by single-base (58), two-base (40), three-base (22), five-base (16), and six-base (2) repeats (**Figure 4B**). As shown in **Supplementary Table S5**, these repeat units are primarily composed of single nucleotide repeats. Both base A and base T repeats occurred 44 times, accounting for 20.85% of all SSRs. There are 40 SSRs with two bases as repeat units, predominantly TA (16 occurrences) (**Figure 4C**; **Supplementary Table S5**). Identification of dispersed repeat sequences in the *M. kwangsiensis* mitochondrial genome is made up of 1101 dispersed repeats, with 466 forward and 635 palindromic repeats (**Figure 4D**). The repeat lengths range from 30 to 8865 bp, with the longest being a forward repeat of 8865 bp and the second-longest being a palindromic repeat of 1242 bp. Dispersed repeat sequences are mainly concentrated within 100 bp, with 266 occurrences between 30 and 39 bp; 302 occurrences between 40 and 49 bp; and 170 occurrences between 50 and 59 bp (**Supplementary Table S6**). Furthermore, 39 LTRs were identified in the *M. kwangsiensis* mitochondrial genome (**Supplementary Table S7**). The repeat units of these tandem repeats mostly range between 10 and 30 bp, with repeat copy numbers between 2 and 3. The longest repeat unit (GTTTTGAAGCTGGCTGTAAAAGCCTATTAGTAAAACGCGCTCATAC) is found in the 12,751-12,864 bp region, with a length of 124.8 bp and a repeat copy number of 2.4. The shortest repeat unit (TATAG) is located in the 404,032-404,063 bp region, with a 32 bp length and a repeat copy number of 2.

### 3.5 Synteny analysis of the *M. kwangsiensis* mitochondrial genome

**Figure 5:**
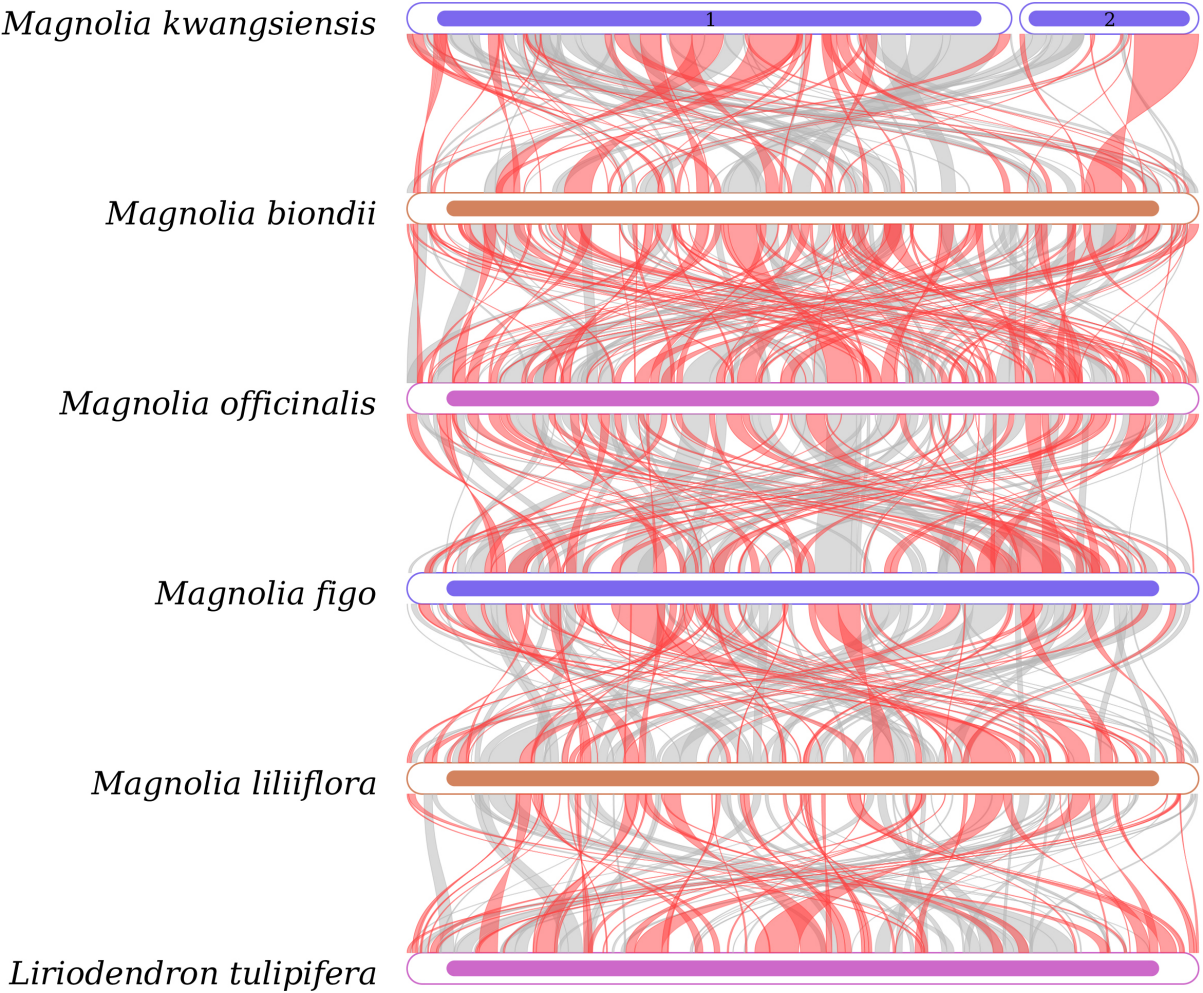
Colinear analysis between *Magnolia kwangsiensis* and Magnoliaceae plants.

Synteny relationship indicates that *M. kwangsiensis* mitochondrial genome exhibits a relatively high similarity with the *L. tulipifera* mitochondrial genome, proving a high synteny degree with species within the genus *Magnolia*, while the similarity with *L. tulipifera* is low (**Supplementary Table S8**). Compared to the mitochondrial genomes of the above-mentioned species in the Magnoliaceae family, large segments of inversion can be observed (**Figure 5**).

### 3.6 Homologous sequence analysis of *M. kwangsiensis* mitochondrial and chloroplast genome

**Figure 6:**
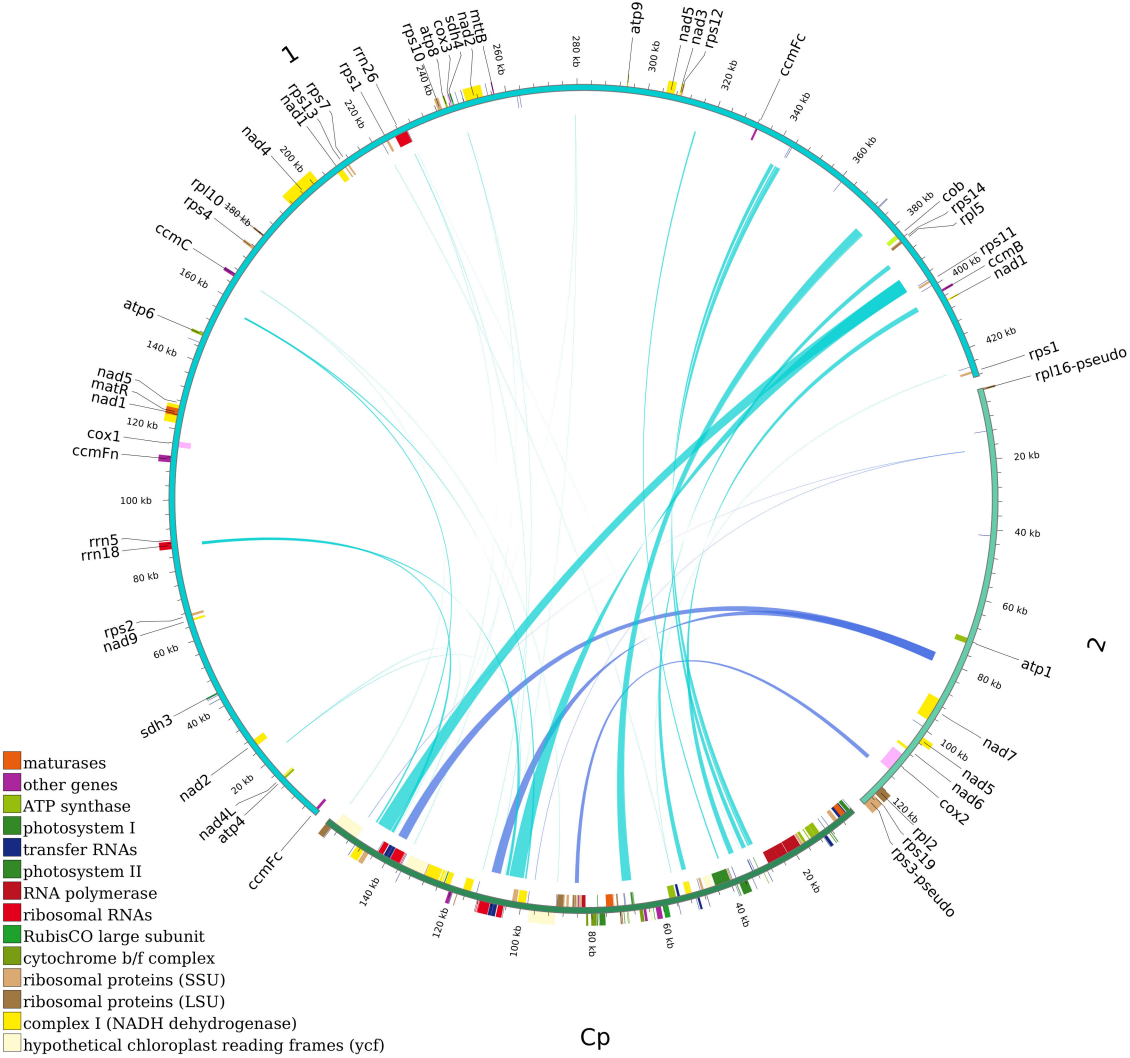
Homologous sequence analysis of chloroplast and mitochondrion in *Magnolia kwangsiensis*.

By assembling the raw sequencing data of *M. kwangsiensis*, we obtained the *M. kwangsiensis* chloroplast genome sequence, which is 159,837 bp long and has a circular structure, with NCBI accession number PQ790171. And 32 homologous fragments were detected between the *M. kwangsiensis* mitochondrial and chloroplast genomes. The complete homologous fragment length is 29,253 bp, with an average 914 bp length. The longest sequence is 4406 bp, while the shortest is only 31 bp. In terms of length distribution, there are 10 fragments longer than 1000 bp, 11 between 100 and 1000 bp, and 11 shorter than 100 bp. Meanwhile, 20 genes were completely transferred, including *rps12*, *rps7*, *trnV-GAC*, *psbF*, *petL*, *psbE*, *petG*, *trnP-UGG*, *trnW-CCA*, *ndhJ*, *trnD-GUC*, *trnE-UUC*, *trnY-GUA*, *petN*, *trnN-GUU*, *trnI-CAU*, *trnM-CAU*, *trnA-UGC*, *rps8*, and *rpl14*. Sixteen genes including *rrn16*, *ndhB*, *psbL*, *ndhK*, *psbD*, *psbC*, *psbJ*, *psbL*, *psbM*, *psaB*, *rrn16*, *trnT-GGU*, *rrn23*, *trnI-GAU*, *infA*, and *ycf2*, showed partial transfer (**Figure 6**; **Supplementary Table S9**).

### 3.7 Homologous sequence of Magnoliaceae mitochondrial genomes

**Figure 7:**
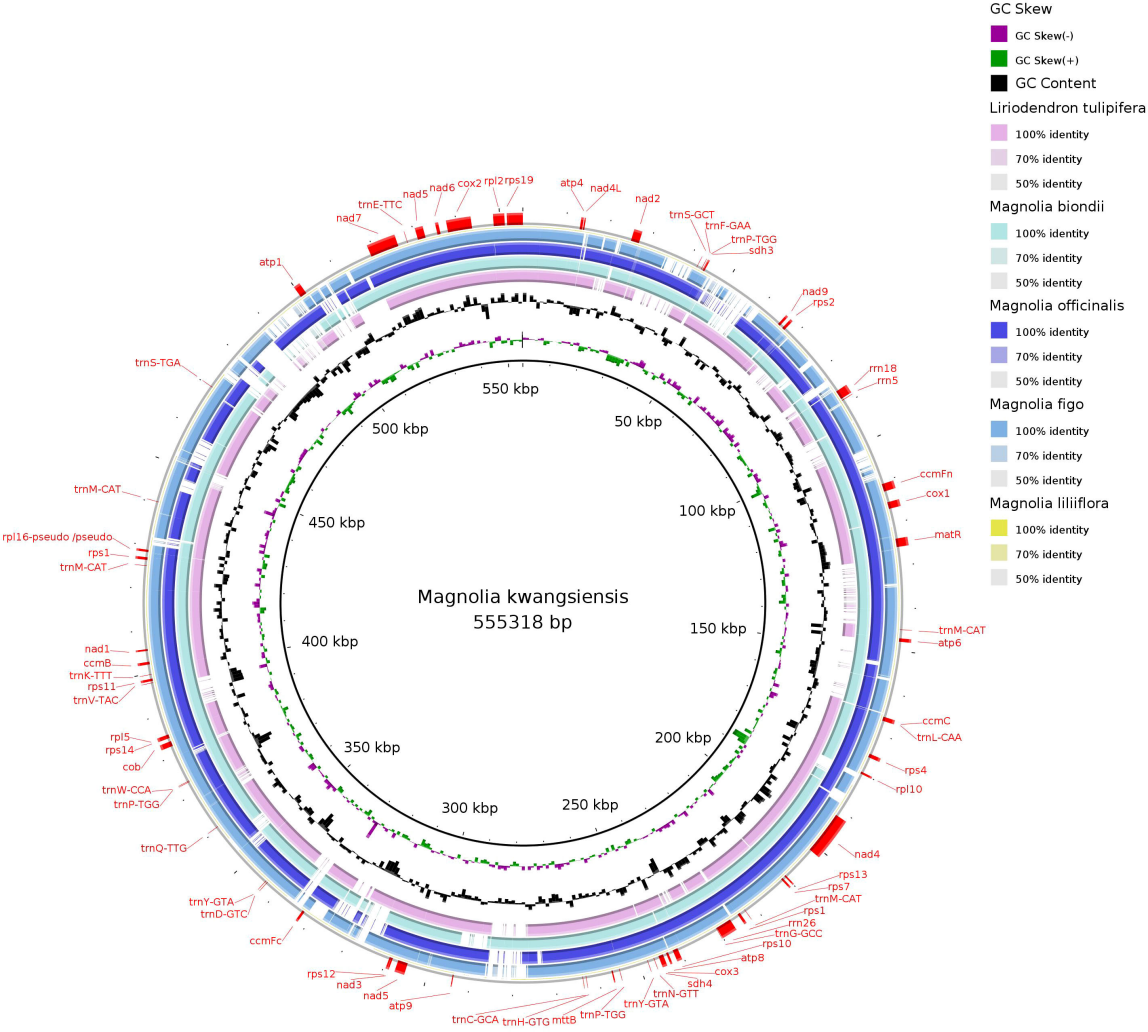
Mitochondrial genome sequence similarity comparison ring map of six Magnoliaceae species.

The *M. kwangsiensis* mitochondrial genome sequence was chosen as the reference sequence, and BRIG software (https://sourceforge.net/projects/brig/) was used to analyze the mitochondrial sequence similarity among six Magnoliaceae species. The results showed a circular plot with not very high overall continuity, indicating varying degrees of differences among the species (**Figure 7**). The difference degree among the species is significantly correlated with their phylogenetic relationships; species within the same genus formed a circular plot with high similarity. Compared to species from other genera, the species within the genus *Magnolia*, such as *M. liliiflora*, *M. officinalis*, and *M. officinalis*, formed a circular plot with high overall continuity, indicating a significantly higher similarity among them than with other species.

### 3.8 Phylogenetic relationship of the *M. kwangsiensis* mitochondrial genome

**Figure 8:**
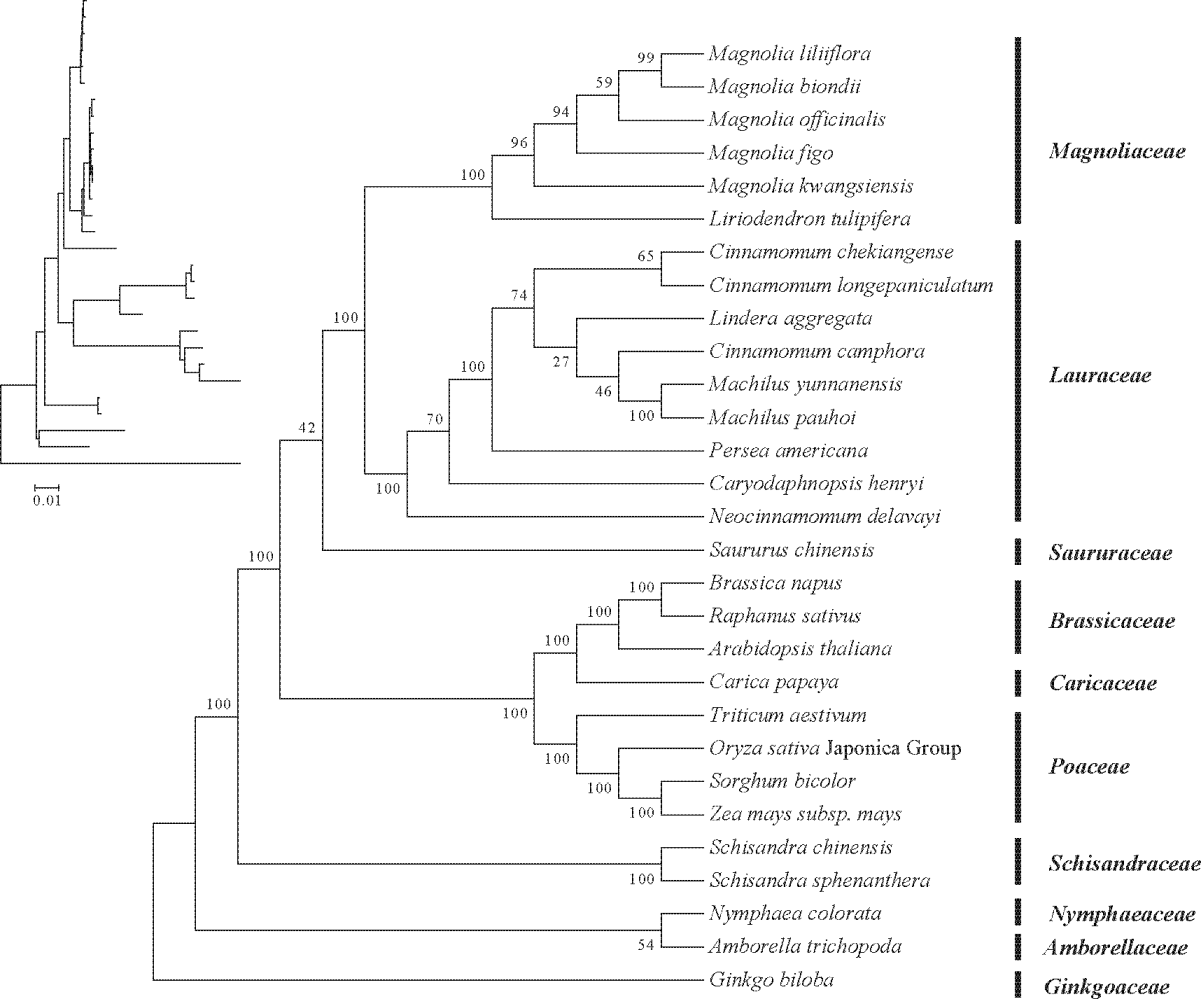
Phylogenetic tree of *Magnolia kwangsiensis* constructed using mitochondrial genomes of 29 representative plants.

Reconstructing phylogenetic trees using conserved mitochondrial PCGs aids in determining the molecular evolutionary relationship (Tang et al., 2023). Based on 28 conserved genes (*atp1*, *atp6*, *atp4*, *atp9*, *atp8*, *ccmB*, *ccmFc*, *ccmC*, *ccmFn*, *cox1*, *cob*, *cox3*, *cox2*, *mttB*, *matR*, *nad1*, *nad3*, *nad2*, *nad4*, *nad4L*, *nad6*, *nad5*, *nad9*, *nad7*, *rpl5*, *rps4*, *rps12*, and *rps7*) from 29 typical plant mitochondrial genomes, a phylogenetic analysis of *M. kwangsiensis* was performed. As seen in **Figure 8**, dicots from monocots and angiosperms from gymnosperms were well separated in this phylogenetic tree, while it also indicates that *M. kwangsiensis* is most closely related to *L. tulipifera* in terms of genetic distance. And the analysis results of the phylogenetic tree of mitochondrial genome are consistent with the classification results in chloroplast genome (**Supplementary Figure S4**).

### 3.9 RNA editing site

**Figure 9:**
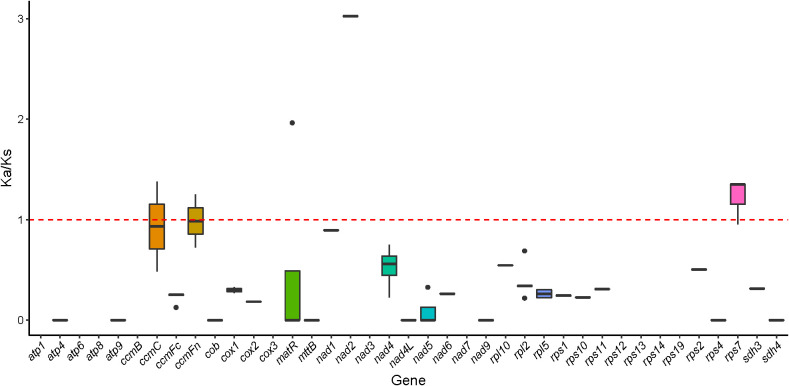
Prediction of RNA editing sites in *Magnolia kwangsiensis*.

In total, 878 RNA editing events were checked out from 39 PCGs (**Figure 9**; **Supplementary Table S9**), primarily manifested as C to T conversions (corresponding to C to U in RNA). The *nad4* gene exhibited the most number, with 68 editing sites discriminated, followed by *nad5* (55 sites). Additionally, *rps11* and *rps7* each had 3 RNA editing sites. Furtherly, the editing events were concentrated at the first and second base of its codon. This RNA editing event resulted in amino acid change, such as leucine (L) to phenylalanine (F), threonine (T) to isoleucine (I), serine (S) to phenylalanine (F), histidine (H) to tyrosine (Y), arginine (R) to cysteine (C), and arginine (R) to tryptophan (W). The number of hydrophilic converted to hydrophobic amino acids was 414, accounting for 47.15%; the number of hydrophobic converted to hydrophobic amino acids was 283, accounting for 32.23%; and the number of hydrophilic converted to hydrophilic amino acids was 97, accounting for 11.05% (**Supplementary Table S10**).

### 3.10 Selection pressure

**Figure 10:**
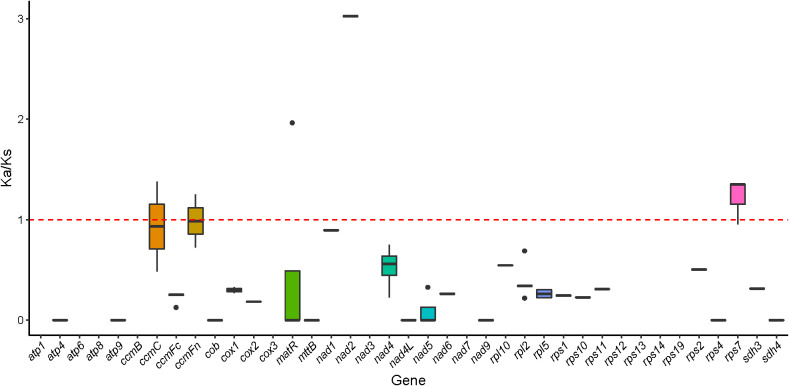
Box plot of Ka/Ks ratio for 39 PCGs of *Magnolia kwangsiensis* in Magnoliaceae family.

The selection pressure analysis of six Magnoliaceae plants found that the average Ka/Ks ratio of 39 homologous proteins was 0.385. Most genes related to respiration, such as *atp1*, *atp4*, *atp6*, *atp8*, *nad1*, *nad2*, *nad3*, *cox1*, *cox2*, *cox3*, etc., had Ka/Ks ratio less than 1, demonstrating a negative selection effect, suggesting that most PCGs in the *M. kwangsiensis* mitochondrial genome are highly conserved during evolution (**Supplementary Table S11**). Notably, the Ka/Ks ratio for *ccmC*, *ccmFn*, and *rps7* were > 1, indicating that these genes may be influenced by positive selection (**Figure 10**).

## 4 Discussion

### 4.1 Mitochondrial structure and composition

Compared with the compact and conservative animal mitochondrial genome, plant mitochondria have many unique characteristics, especially the large and complex genome arrangement. And the plant mitochondrial genome evolves slowly and has high conservativeness, making it an ideal tool for studying species evolution, providing information on phylogenetic relationship, molecular evolution, and species diagnostics (Wang et al., 2024a). With the advancement of genetic analysis methods, the genomes of organelles including mitochondria and chloroplasts, that exist in an endosymbiotic manner and have independent heritability, have gradually been elucidated (Wang et al., 2025).

In light of this, we are conducting research and conservation on *M. kwangsiensis* to better understand and utilize this unique plant resource, maximizing its value. Therefore, a mixed assembly strategy was employed, integrating second-generation and third-generation sequencing methods to analyze the characteristics of the *M. kwangsiensis* mitochondrial genome, concluding that this study validated that mitochondrial genomes can be assembled using reported genomic sequencing data. And the mitochondrial genome size is 555,318 bp, comprising a branched structure that includes a linear and a circular molecule. The structure of this mitochondrial genome is similar to that of *Thuja sutchuenensis*, both being a combination of linear and circular structures (Xia et al., 2023). Compared to previously reported species in Magnoliaceae, the mitochondrial genome size of *M. kwangsiensis* is relatively small, similar to *L. tulipifera* (MK340747.1). GC content is also often made to identify different species (Cheng et al., 2021). The GC content of *M. kwangsiensis* is 47.47%, which is comparable to other plants *Glycyrrhiza glabra* at 45.1% (Zhou et al., 2025), *Scutellaria tsinyunensis* at 45.26% (Li et al., 2021a), and *Angelica dahurica* at 45.06% (Li et al., 2024), with these mitochondrial genome sequences showing similar GC values but higher than that of the *Chlorella heliozoae* (KY629615.1) at 32.7% (Fan et al., 2017) in the family Chlorellaceae. These results suggest that the mitochondrial genomes of Magnoliaceae plants are relatively conservative in GC value but vary greatly in its genome size.

### 4.2 Gene composition and codon usage

In *M. kwangsiensis*, it was mainly composed of non-coding regions, similar to the *Neolamarckia cadamba* mitochondrial genome, where non-coding regions account for 88.61% of the entire genome (Wang et al., 2021). However, PCGs size only account for 5.78%, which may be an increase in sequence duplications during evolution. Most of PCGs use ATG as the start codon, and TAG, TGA, TAA as stop codons, with amino acid distributions similar to *Solanum commersonii* (Cho et al., 2022) and *Acer yangbiense* (Yang et al., 2019). These genes (*cox1*, *nad4L*, *nad1*, and *rps10*) all use ACG and ATG as start codons. Similar phenomena have also been observed in *Suaeda glauca* (Cheng et al., 2021) and *Salix suchowensis* (Ye et al., 2017), which may be related to RNA editing. In the *Acer truncatum* genome studies, it was indicated that *cox1* is associated with horizontal gene transfer (Ma et al., 2022). And in this study, only one *cox1* copy was detected, whereas two *cox1* copies were identified in the *A. truncatum* mitochondrial genome. The mitochondrial codon usage preference is a result of long-term adaptation to the survival environment, possibly related to natural selection factors, and serves as an important method of gene expression regulation (Cheng et al., 2019). The codon preference in the mitochondrial genome is significant for enhancing the gene expression levels in the mitochondria and promoting the plant genetic engineering transformation (Li et al., 2021b). And 31 codons with RSCU values > 1 were revealed, of which 28 end with A/T bases, showing a preference for AT usage, consistent with findings reported in *Luffa cylindrica* (Gong et al., 2024). Furthermore, the codon for leucine (Leu) is the most abundant with 1012 instances, while cysteine (Cys) has the least with 140 instances, which may be related to the proportion increase of hydrophobic amino acids during RNA editing (Yang et al., 2023a).

### 4.3 Ka/Ks value

The size of the Ka/Ks value is often used to assess the natural selection effects on gene selection and reveal potential genetic mechanisms (Tang et al., 2022; Fay and Wu, 2003). Most genes have Ka/Ks values <1, showing that these genes are subject to negative selection and that the highly conserved gene structure plays a crucial role in maintaining mitochondrial function. In contrast, the Ka/Ks values of *ccmFn*, *rps7*, and *ccmC* are >1, indicating that they have undergone positive selection, indicating rapid differentiation that may help the plant adapt better to the environment, similar to the effects observed in the *S. glauca* mitochondrial genome (Cheng et al., 2021). *CcmC* and *ccmFn* may be involved in the maturation process of mitochondrial cytochrome C by regulating the output and metabolism of heme (Robles and Quesada, 2021). *Rps7* is a component of the ribosomal subunit, which is important for the structure and function of ribosomes. It participates in the protein synthesis process within cells, playing a key role in cellular growth, proliferation, and survival (Adams et al., 2002). Overall, these three genes undergoing positive selection, *ccmFn*, *ccmC*, and *rps7* may have developed new functions in the adaptability of *M. kwangsiensis*.

### 4.4 Repeat sequence

The fluctuations in the plant mitochondrial genome size are mainly attributed to variations in intergenic regions, particularly the accumulation of diverse repetitive sequences and the exogenous sequence migration (Kim et al., 2023). Repetitive sequences are crucial for inter- or intra-molecular recombination and they had a key role in shaping the mitochondrial genome. A positive correlation has been found between the repetitive sequence content and the mitochondrial genome size, suggesting that the scale of scattered repeats may promote increases in their mitochondrial genome size. Repetitive sequences include tandem, single-strand, and dispersed repeats, which are widely present in plant genomes (Guo et al., 2017; Cole et al., 2018). The results show that the *M. kwangsiensis* genome contains abundant repetitive sequences, with a total of 211 SSR loci identified. Among all detected repetitive sequences, the monomer repeat number is the highest. The presence of A and T bases in the SSRs is the main reason for the high AT content (54.78%) in the *M. kwangsiensis* genome. Furthermore, this phenomenon has also been observed in *Momordica charantia* (Niu et al., 2023) and *Macadamia integrifolia* (Niu et al., 2022). Additionally, 39 tandem repeat sequences with a match rate greater than 72% were detected in the *M. kwangsiensis* mitochondrial genome. This also indicates that during the *M. kwangsiensis* evolution, inter-molecular recombination frequently originated from the mitochondrial genome (Bi et al., 2016). Repetitive sequences are a vital source of molecular markers for studying population genetics and genome evolution (Wynn and Christensen, 2019). The proportion of repetitive sequences in *Aeginetia indica* is 13.6%, with the longest repetitive sequence being ~16 kb, which has been assembled into linear and circular genomes in different studies (Zhong et al., 2022). In *M. kwangsiensis*, there are 1101 long segment repetitive sequences, comprising 466 forward repeats and 635 palindromic repeats, with the longest being 8865 bp. The existence of these long segment repetitive sequences may be related to its complex mitochondrial genome structure.

### 4.5 RNA editing

RNA editing refers to the phenomenon where, due to the deletion, insertion, or replacement of nucleotides in the mRNA molecules derived from gene transcription, the sequence of gene transcripts does not complement the gene template sequence, resulting in a different amino acid composition in the translated protein compared to the coding information in the gene sequence (Small et al., 2020). Changes in the bases can alter the entire codon, thus leading to amino acid and protein composition changes. RNA editing can stop translation by introducing stop codons, leading to premature termination of gene encoding and even loss of function (Hayes and Santibanez, 2020). And 377 RNA editing sites across 29 genes were reported in *Capsella bursa-pastoris* and 676 RNA editing sites across 35 genes were registered in *Clematis acerifolia* (Omelchenko et al., 2020; Liu et al., 2023). Meanwhile, 878 RNA editing sites were detected across 39 genes in this research, which is more than in *Capsella bursa-pastoris* and *Clematis acerifolia*. The most common type of codon transfer is TCA (S) => TTA (L), with 109 sites. RNA editing results in 8.08% of hydrophobic amino acids being converted to hydrophilicity and 47.15% of hydrophilic amino acids being converted to hydrophobicity. Similar phenomena have been observed in *S. glauca* (Cheng et al., 2021), *Capsella bursa-pastoris* (Omelchenko et al., 2020), and *Clematis acerifolia* (Liu et al., 2023), suggesting that the proportion decrease of hydrophilic amino acids is expected to enhance the overall protein structure stability.

Moreover, TCA (S) ≥ TTA (L) is the most abundant transfer type, with 78 instances in *C. bursa-pastoris* and 101 instances in *Clematis acerifolia*. Additionally, most editing sites in the *M. kwangsiensis* mitochondrial genome are C-T, consistent with the *Quercus acutissima* (Liu et al., 2022) and *Arabidopsis thaliana* mitochondrial genomes (Verhage, 2020). Furtherly, over than half of RNA editing occurs at the second position of codons in previous studies (Robles and Quesada, 2021). And in the *M. kwangsiensis* mitochondrial genome, 68.56% of RNA editing also occurs at the second codon base, similar to the genomes of *Q. acutissima* and *A. thaliana*. Furthermore, the encoded amino acids after RNA editing in the mitochondrial genome become stop codons (TAG, TGA, and TAA). In the *M. kwangsiensis* genome, 0.76% of amino acids become stop codons, leading to mRNA premature termination and gene function alterations. Additionally, the uneven predicted site distribution in the *M. kwangsiensis* mitochondrial genome PCGs suggests that post-transcriptional modifications may be vital for maintaining cytochrome c biosynthesis, complex I, and transport membrane protein functions (**Supplementary Table S10**).

### 4.6 DNA transfer

Sequencing analysis can yield information on DNA fragment transfers between different genomes (chloroplast, nucleus, and mitochondria) (Bergthorsson et al., 2003). And the largest transfer direction in angiosperms is from organelle to the nuclear genome, followed by transfers from the nuclear and chloroplast to mitochondrial genome (Smith and Keeling, 2015). In different higher plants, the transferred DNA size varies, ranging from 50 kb in *A. thaliana* to 1.1 Mb in *Oryza sativa* (Zhao et al., 2018). And 29,253 bp chloroplast DNA fragments were transferred to the *M. kwangsiensis* mitochondrial genome, accounting for 3.53%. The proportion of transferred fragments in the *M. kwangsiensis* genome is higher than that in *A. truncatum* (2.36%) (Ma et al., 2022) and *S. suchowensis* (2.8%) (Ye et al., 2017), but lower than that in *S. glauca* (5.18%) (Cheng et al., 2021). Furthermore, tRNAs are the most common cp genome fragments transferred to the angiosperm mitochondrial genome (Rice et al., 2013). In this research, 32 homologous fragments were transferred from the chloroplast to mitochondrial genome, comprising 20 annotated genes, of which 17 are tRNA genes. Therefore, the inference that chloroplast-derived sequences may not be the main reason for the mitochondrial genome expansion. Further annotation showed that all five tRNA genes are complete, indicating that they still have functionality in this mitochondrial genome. Therefore, sequence exchange was frequent between the *M. kwangsiensis* mitochondrial and chloroplast genomes. Further research is needed to investigate the relationship between the transferred fragments and the sterile genes in the mitochondrial genome of *M. kwangsiensis.*

### 4.7 Homology and phylogeny

Homologous region rearrangement has been widely utilized to explore the phylogenetic status, and homologous analysis provides an effective manner to interpret the genome arrangement (Zhang et al., 2024b). This study found low homology among Magnoliaceae plants, with irregular arrangements of these homologous sequences, indicating a significant amount of gene rearrangement in the comparative mitochondrial genomes. Plant mitochondrial genomes typically exhibit high variability due to a high frequency of homologous recombination (Yang et al., 2023b). However, the gene order and clusters remain conserved among closely related species during evolution (Xue et al., 2022). Using PCGs to construct a phylogenetic tree, we analyzed the evolutionary relationship between *M. kwangsiensis* and its representative species from other taxonomic groups. Phylogenetic tree analysis indicates that Magnoliaceae is positioned at the base of angiosperms and can be well distinguished from other monocots and dicots. This phylogenetic structure based on mitochondrial DNA reflects clear taxonomic relationships, and our results test the effectiveness of mitochondrial genome data in revealing taxonomic relationships in the family level.

## 5 Conclusions

This study describes and compares the complete *M. kwangsiensis* mitochondrial genome with the mitochondrial genomes of other reported Magnoliaceae species for the first time. We successfully elucidated the *M. kwangsiensis* mitochondrial genome with integrating PacBio and Illumina sequencing data, revealing the complex combination structure of the linear and circular mitochondrial genome. This mitochondrial genome exhibits a significant AT preference in codon usage and retains a considerable number of sequence fragments derived from the chloroplast genome. By comparing the *M. kwangsiensis* mitochondrial genome with several Magnoliaceae mitochondrial genomes in terms of RNA editing site prediction, repetitive sequence identification, selection pressure estimation, homologous sequence and structural inference, and phylogenetic analysis, significant genomic variations were found that contribute to our deepened understanding of higher plant mitochondrial genome diversity and evolution. In summary, the knowledge obtained from this mitochondrial genome research provides new insights into the Magnoliaceae genetics, laying a foundation for further genetic engineering and breeding improvements involving *M. kwangsiensis*.

## Data Availability

The datasets generated and analysed during the current study are available in the NCBI public database (https://www.ncbi.nlm.nih.gov/nuccore/PQ720776PQ822236/; https://www.ncbi.nlm.nih.gov/nuccore/PQ822237/; and https://www.ncbi.nlm.nih.gov/nuccore/PQ790171/), and the corresponding accession number was PQ822236, PQ822237 and PQ790171.
