# Supplementary figures and images for: The complex DNA molecular combination with a linear and circular structure in Magnolia kwangsiensis mitochondrial genome
Source: Front Plant Sci. 2025 May 29;16:1590173. doi: 10.3389/fpls.2025.1590173 (PMC12158950; doi:10.3389/fpls.2025.1590173)

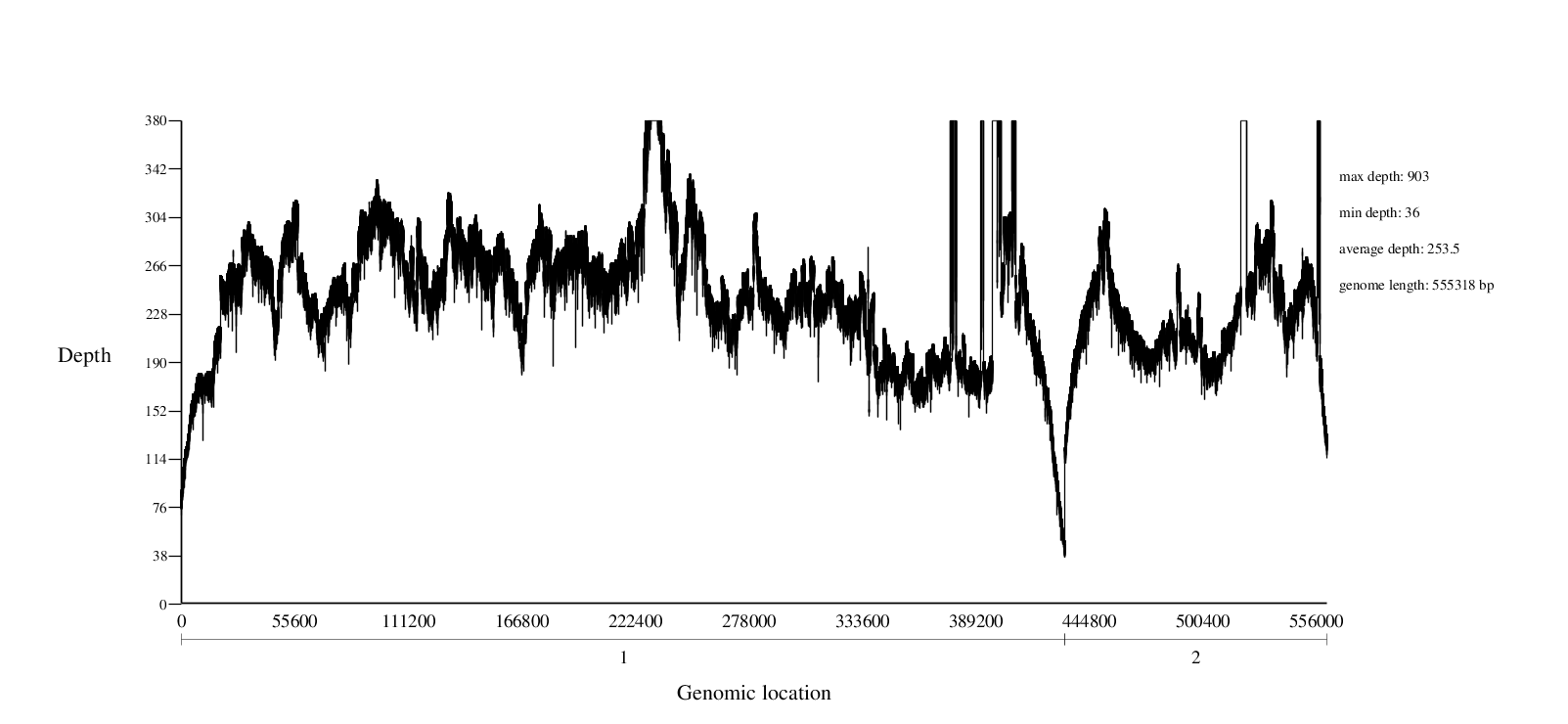

Supplement: Supplementary file 2 [file Image2.tif]

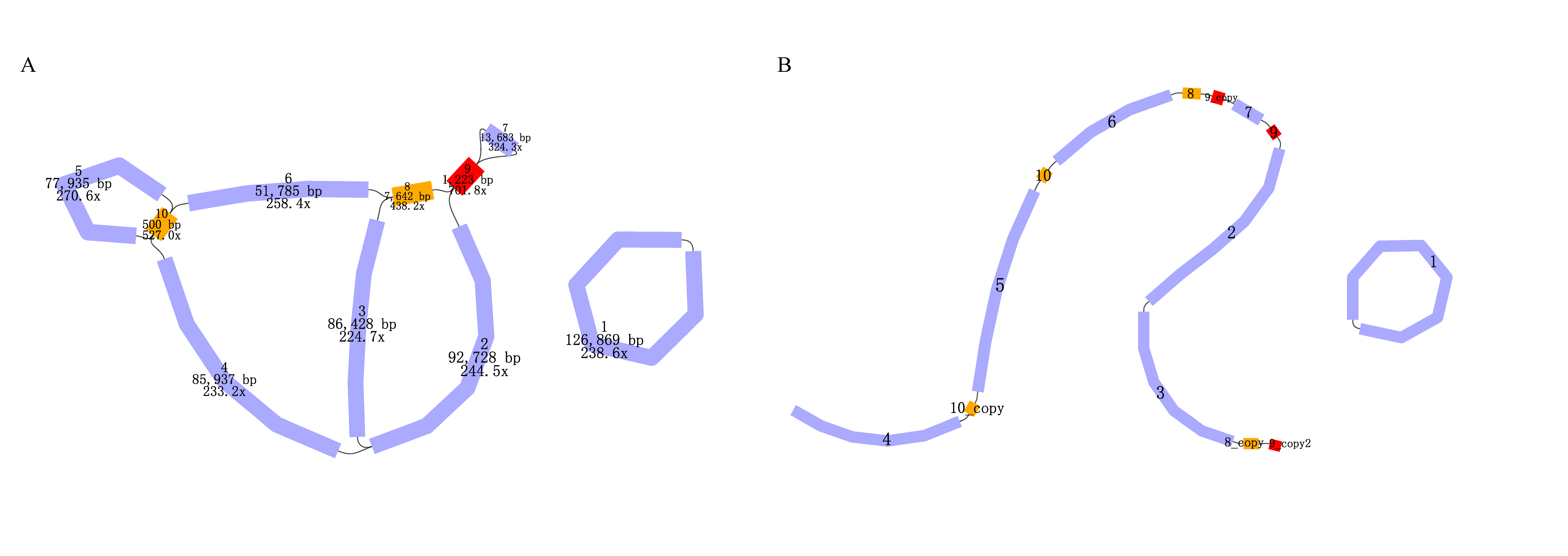

Supplement: Supplementary file 3 [file Image3.tif]
