# Supplementary material for: The complex DNA molecular combination with a linear and circular structure in Magnolia kwangsiensis mitochondrial genome
Source: Front Plant Sci. 2025 May 29;16:1590173. doi: 10.3389/fpls.2025.1590173 (PMC12158950; doi:10.3389/fpls.2025.1590173)
Supplement: Supplementary file 5 [file Table1.docx]

**Supplementary table 1. Gene composition in** ***Magnolia kwangsiensis* mitochondrial genome**

| **Group of genes** | **Gene name** |
| --- | --- |
| ATP synthase | *atp1,atp4, atp6, atp8, atp9* |
| Cytohrome c biogenesis | *ccmB, ccmC, ccmFc*, ccmFn* |
| Ubiquinol cytochrome c reductase | *cob* |
| Cytochrome c oxidase | *cox1, cox2**, cox3* |
| Maturases | *matR* |
| Transport membrane protein | *mttB* |
| NADH dehydrogenase | *nad1****, nad2****, nad3, nad4***, nad4L, nad5****, nad6, nad7****, nad9* |
| Ribosomal proteins (LSU) | *#rpl16, rpl10, rpl2*, rpl5* |
| Ribosomal proteins (SSU) | *#rps3, rps1(2), rps10*, rps11, rps12, rps13, rps14, rps19, rps2, rps4, rps7* |
| Succinate dehydrogenase | *sdh3, sdh4* |
| Ribosomal RNAs | *rrn18, rrn26, rrn5* |
| Transfer RNAs | *trnC-GCA, trnD-GTC, trnE-TTC, trnF-GAA, trnG-GCC, trnH-GTG, trnK-TTT, trnL-CAA, trnM-CAT(4), trnN-GTT, trnP-TGG(3), trnQ-TTG, trnS-GCT, trnS-TGA, trnV-TAC, trnW-CCA, trnY-GTA(2)* |

Notes: *:intron number; #Gene:Pseudo gene; Gene(2):Number of copies of multi-copy genes;

**Supplementary table 2. Gene characteristics in *Magnolia kwangsiensis* mitochondrial genome**

| **Group of genes** | **Gene name** | **Length** | **Start codon** | **Stop codon** | **Amino acid** |
| --- | --- | --- | --- | --- | --- |
| ATP synthase | *atp1* | 1530 | ATG | TGA | 510 |
|  | *atp4* | 582 | ATG | TAA | 194 |
|  | *atp6* | 795 | ATG | CAA(TAA) | 265 |
|  | *atp8* | 480 | ATG | TAA | 160 |
|  | *atp9* | 225 | ATG | CGA(TGA) | 75 |
| Cytohrome c biogenesis | *ccmB* | 621 | ATG | TGA | 207 |
|  | *ccmC* | 960 | ATG | TAA | 320 |
|  | *ccmFc* | 1329 | ATG | CGA(TGA) | 443 |
|  | *ccmFn* | 1806 | ATG | TAG | 602 |
| Ubichinol cytochrome c reductase | *cob* | 1182 | ATG | TGA | 394 |
| Cytochrome c oxidase | *cox1* | 1584 | ACG(ATG) | TAA | 528 |
|  | *cox2* | 765 | ATG | TAA | 255 |
|  | *cox3* | 798 | ATG | TGA | 266 |
| Maturases | *matR* | 1959 | ATG | TAG | 653 |
| Transport membrance protein | *mttB* | 291 | ATG | TGA | 97 |
| NADH dehydrogenase | *nad1* | 978 | ACG(ATG) | TAA | 326 |
|  | *nad2* | 1467 | ATG | TAA | 489 |
|  | *nad3* | 357 | ATG | TAA | 119 |
|  | *nad4* | 1488 | ATG | TGA | 496 |
|  | *nad4L* | 303 | ACG(ATG) | TAA | 101 |
|  | *nad5* | 2013 | ATG | TAA | 671 |
|  | *nad6* | 735 | ATG | TGA | 245 |
|  | *nad7* | 1185 | ATG | TAG | 395 |
|  | *nad9* | 573 | ATG | TAA | 191 |
| Ribosomal proteins (LSU) | *rpl10* | 480 | ATG | TAA | 160 |
|  | *rpl2* | 945 | ATG | not determined | 315 |
|  | *rpl5* | 561 | ATG | TAA | 187 |
| Ribosomal proteins (SSU) | *rps1* | 606 | ATG | TAA | 202 |
|  | *rps1* | 606 | ATG | TAA | 202 |
|  | *rps10* | 360 | ACG(ATG) | TGA | 120 |
|  | *rps11* | 552 | ATG | TGA | 184 |
|  | *rps12* | 378 | ATG | TGA | 126 |
|  | *rps13* | 351 | ATG | TGA | 117 |
|  | *rps14* | 303 | ATG | TAG | 101 |
|  | *rps19* | 282 | ATG | TAA | 94 |
|  | *rps2* | 657 | ATG | TAA | 219 |
|  | *rps4* | 843 | ATG | TAA | 281 |
|  | *rps7* | 450 | ATG | TAA | 150 |
| Succinate dehydrogenase | *sdh3* | 330 | ATG | TAA | 110 |
|  | *sdh4* | 384 | ATG | CGA(TGA) | 128 |
| Ribosomal RNAs | *rrn18* | 2086 |  |  |  |
|  | *rrn26* | 3669 |  |  |  |
|  | *rrn5* | 121 |  |  |  |
| Transfer RNAs | *trnC-GCA* | 71 |  |  |  |
|  | *trnD-GTC* | 74 |  |  |  |
|  | *trnE-TTC* | 72 |  |  |  |
|  | *trnF-GAA* | 74 |  |  |  |
|  | *trnG-GCC* | 72 |  |  |  |
|  | *trnH-GTG* | 74 |  |  |  |
|  | *trnK-TTT* | 73 |  |  |  |
|  | *trnL-CAA* | 74 |  |  |  |
|  | *trnM-CAT* | 74 |  |  |  |
|  | *trnM-CAT* | 73 |  |  |  |
|  | *trnM-CAT* | 73 |  |  |  |
|  | *trnM-CAT* | 74 |  |  |  |
|  | *trnN-GTT* | 72 |  |  |  |
|  | *trnP-TGG* | 75 |  |  |  |
|  | *trnP-TGG* | 75 |  |  |  |
|  | *trnP-TGG* | 74 |  |  |  |
|  | *trnQ-TTG* | 72 |  |  |  |
|  | *trnS-GCT* | 88 |  |  |  |
|  | *trnS-TGA* | 87 |  |  |  |
|  | *trnV-TAC* | 73 |  |  |  |
|  | *trnW-CCA* | 74 |  |  |  |
|  | *trnY-GTA* | 83 |  |  |  |

**Supplementary table 3. Codon and RSCU analysis in *Magnolia kwangsiensis* mitochondrial genome**

| **AminoAcid** | **Symbol** | **Codon** | **No.** | **RSCU** |
| --- | --- | --- | --- | --- |
| * | Ter | UAA | 20 | 1.5789 |
| * | Ter | UAG | 4 | 0.3158 |
| * | Ter | UGA | 14 | 1.1053 |
| A | Ala | GCA | 171 | 1.0015 |
| A | Ala | GCC | 155 | 0.9078 |
| A | Ala | GCG | 83 | 0.4861 |
| A | Ala | GCU | 274 | 1.6047 |
| C | Cys | UGC | 63 | 0.9 |
| C | Cys | UGU | 77 | 1.1 |
| D | Asp | GAC | 96 | 0.563 |
| D | Asp | GAU | 245 | 1.437 |
| E | Glu | GAA | 289 | 1.3536 |
| E | Glu | GAG | 138 | 0.6464 |
| F | Phe | UUC | 288 | 0.9381 |
| F | Phe | UUU | 326 | 1.0619 |
| G | Gly | GGA | 262 | 1.4637 |
| G | Gly | GGC | 92 | 0.514 |
| G | Gly | GGG | 132 | 0.7374 |
| G | Gly | GGU | 230 | 1.2849 |
| H | His | CAC | 69 | 0.4911 |
| H | His | CAU | 212 | 1.5089 |
| I | Ile | AUA | 191 | 0.7244 |
| I | Ile | AUC | 246 | 0.933 |
| I | Ile | AUU | 354 | 1.3426 |
| K | Lys | AAA | 254 | 1.2182 |
| K | Lys | AAG | 163 | 0.7818 |
| L | Leu | CUA | 144 | 0.8538 |
| L | Leu | CUC | 117 | 0.6937 |
| L | Leu | CUG | 98 | 0.581 |
| L | Leu | CUU | 228 | 1.3518 |
| L | Leu | UUA | 221 | 1.3103 |
| L | Leu | UUG | 204 | 1.2095 |
| M | Met | AUG | 296 | 1 |
| N | Asn | AAC | 112 | 0.6707 |
| N | Asn | AAU | 222 | 1.3293 |
| P | Pro | CCA | 193 | 1.154 |
| P | Pro | CCC | 141 | 0.843 |
| P | Pro | CCG | 109 | 0.6517 |
| P | Pro | CCU | 226 | 1.3513 |
| Q | Gln | CAA | 225 | 1.4754 |
| Q | Gln | CAG | 80 | 0.5246 |
| R | Arg | AGA | 168 | 1.3567 |
| R | Arg | AGG | 89 | 0.7187 |
| R | Arg | CGA | 162 | 1.3082 |
| R | Arg | CGC | 73 | 0.5895 |
| R | Arg | CGG | 97 | 0.7833 |
| R | Arg | CGU | 154 | 1.2436 |
| S | Ser | AGC | 102 | 0.5834 |
| S | Ser | AGU | 163 | 0.9323 |
| S | Ser | UCA | 203 | 1.1611 |
| S | Ser | UCC | 180 | 1.0296 |
| S | Ser | UCG | 164 | 0.938 |
| S | Ser | UCU | 237 | 1.3556 |
| T | Thr | ACA | 130 | 0.9524 |
| T | Thr | ACC | 145 | 1.0623 |
| T | Thr | ACG | 80 | 0.5861 |
| T | Thr | ACU | 191 | 1.3993 |
| V | Val | GUA | 197 | 1.1976 |
| V | Val | GUC | 121 | 0.7356 |
| V | Val | GUG | 152 | 0.924 |
| V | Val | GUU | 188 | 1.1429 |
| W | Trp | UGG | 145 | 1 |
| Y | Tyr | UAC | 68 | 0.4674 |
| Y | Tyr | UAU | 223 | 1.5326 |

**Supplementary table 4. Pi value analysis in Poaceae**

| #No. | Region | Pi | Total Number of mutations | Region length |
| --- | --- | --- | --- | --- |
| 1 | atp1 | 0.00022 | 1 | 1536 |
| 2 | atp4 | 0.00057 | 1 | 582 |
| 3 | atp6 | 0.00231 | 3 | 891 |
| 4 | atp8 | 0.00208 | 3 | 480 |
| 5 | atp9 | 0.04385 | 29 | 278 |
| 6 | ccmB | 0.00161 | 3 | 621 |
| 7 | ccmC | 0.00472 | 9 | 960 |
| 8 | ccmFc | 0.0013 | 3 | 1917 |
| 9 | ccmFn | 0.00122 | 6 | 1806 |
| 10 | cob | 0.00085 | 3 | 1182 |
| 11 | cox1 | 0.00084 | 4 | 1584 |
| 12 | cox2 | 0.00132 | 3 | 765 |
| 13 | cox3 | 0.00042 | 1 | 798 |
| 14 | matR | 0.00321 | 18 | 1959 |
| 15 | mttB | 0.00229 | 2 | 768 |
| 16 | nad1 | 0.05312 | 153 | 996 |
| 17 | nad2 | 0.00091 | 4 | 1467 |
| 18 | nad3 | 0 | 0 | 357 |
| 19 | nad4 | 0.00278 | 10 | 1488 |
| 20 | nad4L | 0.00176 | 1 | 303 |
| 21 | nad5 | 0.00023 | 1 | 2580 |
| 22 | nad6 | 0.0271 | 57 | 757 |
| 23 | nad7 | 0.00073 | 2 | 1185 |
| 24 | nad9 | 0.00151 | 2 | 573 |
| 25 | rpl10 | 0.00071 | 1 | 490 |
| 26 | rpl2 | 0.00423 | 12 | 1686 |
| 27 | rpl5 | 0.00547 | 8 | 561 |
| 28 | rps1 | 0.00473 | 6 | 703 |
| 29 | rps10 | 0.00796 | 8 | 420 |
| 30 | rps11 | 0.00444 | 5 | 552 |
| 31 | rps12 | 0 | 0 | 378 |
| 32 | rps13 | 0.00095 | 1 | 351 |
| 33 | rps14 | 0.0011 | 1 | 303 |
| 34 | rps19 | 0.00355 | 3 | 288 |
| 35 | rps2 | 0.00409 | 7 | 667 |
| 36 | rps4 | 0.00142 | 3 | 1071 |
| 37 | rps7 | 0.00904 | 8 | 450 |
| 38 | rrn18 | 0.00049 | 2 | 2087 |
| 39 | rrn26 | 0.00237 | 13 | 3715 |
| 40 | rrn5 | 0 | 0 | 121 |
| 41 | sdh3 | 0.00303 | 3 | 330 |
| 42 | sdh4 | 0.00174 | 2 | 447 |

**Supplementary table 5. SSR type in *Magnolia kwangsiensis* mitochondrial genome**

| ID | SSR nr. | SSR type | SSR | size | start | end |
| --- | --- | --- | --- | --- | --- | --- |
| chr1 | 1 | p2 | (TC)5 | 10 | 8448 | 8457 |
| chr1 | 2 | p1 | (T)15 | 15 | 10623 | 10637 |
| chr1 | 3 | p1 | (A)11 | 11 | 15727 | 15737 |
| chr1 | 4 | p2 | (TA)5 | 10 | 18925 | 18934 |
| chr1 | 5 | p3 | (AGT)4 | 12 | 20146 | 20157 |
| chr1 | 6 | p4 | (AGGA)3 | 12 | 20628 | 20639 |
| chr1 | 7 | p5 | (AGAAT)3 | 15 | 24564 | 24578 |
| chr1 | 8 | p2 | (TA)5 | 10 | 25577 | 25586 |
| chr1 | 9 | p4 | (CTTT)3 | 12 | 29144 | 29155 |
| chr1 | 10 | p3 | (AGA)4 | 12 | 30792 | 30803 |
| chr1 | 11 | p2 | (TA)5 | 10 | 34711 | 34720 |
| chr1 | 12 | p2 | (AT)5 | 10 | 36955 | 36964 |
| chr1 | 13 | p1 | (T)10 | 10 | 39309 | 39318 |
| chr1 | 14 | p4 | (GCTT)3 | 12 | 41641 | 41652 |
| chr1 | 15 | p5 | (GTAAT)3 | 15 | 41840 | 41854 |
| chr1 | 16 | p1 | (G)10 | 10 | 43719 | 43728 |
| chr1 | 17 | p4 | (CAAC)3 | 12 | 51235 | 51246 |
| chr1 | 18 | p2 | (CT)5 | 10 | 52355 | 52364 |
| chr1 | 19 | p1 | (T)10 | 10 | 52975 | 52984 |
| chr1 | 20 | p2 | (GA)5 | 10 | 62037 | 62046 |
| chr1 | 21 | p4 | (CCAT)3 | 12 | 62104 | 62115 |
| chr1 | 22 | p2 | (TC)7 | 14 | 64694 | 64707 |
| chr1 | 23 | p1 | (T)10 | 10 | 67479 | 67488 |
| chr1 | 24 | p4 | (AAGC)3 | 12 | 69055 | 69066 |
| chr1 | 25 | p4 | (CCGC)3 | 12 | 69693 | 69704 |
| chr1 | 26 | p4 | (TGGG)3 | 12 | 70805 | 70816 |
| chr1 | 27 | p1 | (T)10 | 10 | 71529 | 71538 |
| chr1 | 28 | p2 | (TC)5 | 10 | 74049 | 74058 |
| chr1 | 29 | p4 | (GGCG)3 | 12 | 88693 | 88704 |
| chr1 | 30 | p3 | (GTT)4 | 12 | 97513 | 97524 |
| chr1 | 31 | p1 | (T)11 | 11 | 103426 | 103436 |
| chr1 | 32 | p2 | (TC)6 | 12 | 106581 | 106592 |
| chr1 | 33 | p4 | (CAAA)3 | 12 | 109244 | 109255 |
| chr1 | 34 | p5 | (CTATA)4 | 20 | 112538 | 112557 |
| chr1 | 35 | p4 | (GGAA)3 | 12 | 113470 | 113481 |
| chr1 | 36 | p3 | (TGT)4 | 12 | 117663 | 117674 |
| chr1 | 37 | p1 | (T)10 | 10 | 121077 | 121086 |
| chr1 | 38 | p2 | (CT)5 | 10 | 121250 | 121259 |
| chr1 | 39 | p5 | (CTAGT)3 | 15 | 125677 | 125691 |
| chr1 | 40 | p4 | (AATG)3 | 12 | 126066 | 126077 |
| chr1 | 41 | p4 | (GCTT)3 | 12 | 126183 | 126194 |
| chr1 | 42 | p1 | (T)12 | 12 | 135841 | 135852 |
| chr1 | 43 | p2 | (CT)5 | 10 | 138318 | 138327 |
| chr1 | 44 | p4 | (AGCA)3 | 12 | 140225 | 140236 |
| chr1 | 45 | p1 | (T)11 | 11 | 141043 | 141053 |
| chr1 | 46 | p1 | (A)10 | 10 | 143006 | 143015 |
| chr1 | 47 | p4 | (GAAA)3 | 12 | 144151 | 144162 |
| chr1 | 48 | p3 | (CGC)4 | 12 | 144940 | 144951 |
| chr1 | 49 | p1 | (A)11 | 11 | 145990 | 146000 |
| chr1 | 50 | p4 | (TTAT)3 | 12 | 147858 | 147869 |
| chr1 | 51 | p2 | (AT)5 | 10 | 153800 | 153809 |
| chr1 | 52 | p3 | (AGA)4 | 12 | 155463 | 155474 |
| chr1 | 53 | p4 | (AGTA)4 | 16 | 160833 | 160848 |
| chr1 | 54 | p3 | (GGA)4 | 12 | 165466 | 165477 |
| chr1 | 55 | p2 | (CT)5 | 10 | 167619 | 167628 |
| chr1 | 56 | p6 | (CTATTT)3 | 18 | 168164 | 168181 |
| chr1 | 57 | p4 | (CTTG)3 | 12 | 168258 | 168269 |
| chr1 | 58 | p5 | (GTGAG)3 | 15 | 168644 | 168658 |
| chr1 | 59 | p5 | (GTAGT)3 | 15 | 168663 | 168677 |
| chr1 | 60 | p2 | (GA)5 | 10 | 169366 | 169375 |
| chr1 | 61 | p4 | (AGCC)3 | 12 | 171321 | 171332 |
| chr1 | 62 | p4 | (AAAT)3 | 12 | 171892 | 171903 |
| chr1 | 63 | p2 | (AT)5 | 10 | 175633 | 175642 |
| chr1 | 64 | p1 | (T)10 | 10 | 176291 | 176300 |
| chr1 | 65 | p3 | (TTA)6 | 18 | 178465 | 178482 |
| chr1 | 66 | p1 | (A)11 | 11 | 180240 | 180250 |
| chr1 | 67 | p2 | (TA)6 | 12 | 180284 | 180295 |
| chr1 | 68 | p2 | (TA)6 | 12 | 180300 | 180311 |
| chr1 | 69 | p4 | (GATA)3 | 12 | 181417 | 181428 |
| chr1 | 70 | p4 | (TAGA)3 | 12 | 181464 | 181475 |
| chr1 | 71 | p3 | (TGA)5 | 15 | 182566 | 182580 |
| chr1 | 72 | p2 | (TA)5 | 10 | 183661 | 183670 |
| chr1 | 73 | p1 | (A)10 | 10 | 190312 | 190321 |
| chr1 | 74 | p5 | (TTTCA)3 | 15 | 190566 | 190580 |
| chr1 | 75 | p1 | (G)12 | 12 | 191182 | 191193 |
| chr1 | 76 | p1 | (T)11 | 11 | 192041 | 192051 |
| chr1 | 77 | p5 | (GTCCT)3 | 15 | 192554 | 192568 |
| chr1 | 78 | p2 | (AG)5 | 10 | 192777 | 192786 |
| chr1 | 79 | p3 | (GCA)4 | 12 | 193983 | 193994 |
| chr1 | 80 | p3 | (TGT)4 | 12 | 194447 | 194458 |
| chr1 | 81 | p4 | (TAGC)3 | 12 | 197071 | 197082 |
| chr1 | 82 | p1 | (A)10 | 10 | 198605 | 198614 |
| chr1 | 83 | p1 | (G)10 | 10 | 200081 | 200090 |
| chr1 | 84 | p4 | (TTGC)3 | 12 | 202171 | 202182 |
| chr1 | 85 | p5 | (GAAGG)3 | 15 | 203125 | 203139 |
| chr1 | 86 | p4 | (GTTG)3 | 12 | 211624 | 211635 |
| chr1 | 87 | p1 | (A)13 | 13 | 221803 | 221815 |
| chr1 | 88 | p4 | (GAAT)3 | 12 | 222885 | 222896 |
| chr1 | 89 | p2 | (TC)7 | 14 | 223602 | 223615 |
| chr1 | 90 | p3 | (GAA)4 | 12 | 223899 | 223910 |
| chr1 | 91 | p1 | (A)10 | 10 | 237812 | 237821 |
| chr1 | 92 | p4 | (AACT)3 | 12 | 238029 | 238040 |
| chr1 | 93 | p4 | (CAAC)3 | 12 | 242778 | 242789 |
| chr1 | 94 | p4 | (AGCA)3 | 12 | 245433 | 245444 |
| chr1 | 95 | p4 | (GGAT)3 | 12 | 246774 | 246785 |
| chr1 | 96 | p1 | (C)10 | 10 | 248420 | 248429 |
| chr1 | 97 | p1 | (A)11 | 11 | 249231 | 249241 |
| chr1 | 98 | p2 | (CT)6 | 12 | 250074 | 250085 |
| chr1 | 99 | p1 | (A)12 | 12 | 250214 | 250225 |
| chr1 | 100 | p2 | (AT)5 | 10 | 252558 | 252567 |
| chr1 | 101 | p2 | (CT)6 | 12 | 254082 | 254093 |
| chr1 | 102 | p1 | (A)11 | 11 | 257808 | 257818 |
| chr1 | 103 | p2 | (TA)5 | 10 | 259424 | 259433 |
| chr1 | 104 | p4 | (ATCT)3 | 12 | 259746 | 259757 |
| chr1 | 105 | p3 | (GCT)4 | 12 | 261436 | 261447 |
| chr1 | 106 | p1 | (A)11 | 11 | 262236 | 262246 |
| chr1 | 107 | p1 | (A)10 | 10 | 265206 | 265215 |
| chr1 | 108 | p5 | (AAGTC)3 | 15 | 271861 | 271875 |
| chr1 | 109 | p2 | (AG)5 | 10 | 276722 | 276731 |
| chr1 | 110 | p5 | (TCTTT)3 | 15 | 276885 | 276899 |
| chr1 | 111 | p4 | (AAGA)3 | 12 | 277177 | 277188 |
| chr1 | 112 | p4 | (AAGC)3 | 12 | 282715 | 282726 |
| chr1 | 113 | p4 | (CTTA)3 | 12 | 289999 | 290010 |
| chr1 | 114 | p5 | (GAAAT)3 | 15 | 291383 | 291397 |
| chr1 | 115 | p4 | (ATTC)3 | 12 | 292303 | 292314 |
| chr1 | 116 | p4 | (AACC)3 | 12 | 295669 | 295680 |
| chr1 | 117 | p1 | (G)13 | 13 | 301352 | 301364 |
| chr1 | 118 | p4 | (GCCG)3 | 12 | 307978 | 307989 |
| chr1 | 119 | p1 | (C)10 | 10 | 308188 | 308197 |
| chr1 | 120 | p1 | (T)16 | 16 | 308612 | 308627 |
| chr1 | 121 | p4 | (TTGA)3 | 12 | 311212 | 311223 |
| chr1 | 122 | p2 | (AT)5 | 10 | 316194 | 316203 |
| chr1 | 123 | p2 | (TA)5 | 10 | 316803 | 316812 |
| chr1 | 124 | p1 | (T)10 | 10 | 319729 | 319738 |
| chr1 | 125 | p4 | (ATAA)3 | 12 | 319802 | 319813 |
| chr1 | 126 | p1 | (A)13 | 13 | 329259 | 329271 |
| chr1 | 127 | p1 | (C)10 | 10 | 329909 | 329918 |
| chr1 | 128 | p2 | (TA)5 | 10 | 334895 | 334904 |
| chr1 | 129 | p4 | (ACTT)3 | 12 | 337538 | 337549 |
| chr1 | 130 | p4 | (TTTC)3 | 12 | 339107 | 339118 |
| chr1 | 131 | p1 | (T)11 | 11 | 340058 | 340068 |
| chr1 | 132 | p1 | (A)14 | 14 | 340880 | 340893 |
| chr1 | 133 | p1 | (A)11 | 11 | 342034 | 342044 |
| chr1 | 134 | p1 | (T)13 | 13 | 347856 | 347868 |
| chr1 | 135 | p1 | (A)16 | 16 | 348518 | 348533 |
| chr1 | 136 | p2 | (AG)6 | 12 | 348615 | 348626 |
| chr1 | 137 | p3 | (CAG)4 | 12 | 355758 | 355769 |
| chr1 | 138 | p5 | (ATTCT)4 | 20 | 358126 | 358145 |
| chr1 | 139 | p4 | (CCTT)3 | 12 | 358165 | 358176 |
| chr1 | 140 | p1 | (T)10 | 10 | 358641 | 358650 |
| chr1 | 141 | p5 | (CCCCT)3 | 15 | 359374 | 359388 |
| chr1 | 142 | p4 | (AGAA)4 | 16 | 361097 | 361112 |
| chr1 | 143 | p4 | (ATGA)3 | 12 | 361282 | 361293 |
| chr1 | 144 | p1 | (G)10 | 10 | 361938 | 361947 |
| chr1 | 145 | p1 | (G)10 | 10 | 364888 | 364897 |
| chr1 | 146 | p4 | (AAGG)3 | 12 | 369700 | 369711 |
| chr1 | 147 | p1 | (A)10 | 10 | 373459 | 373468 |
| chr1 | 148 | p1 | (T)14 | 14 | 373883 | 373896 |
| chr1 | 149 | p2 | (AC)5 | 10 | 376960 | 376969 |
| chr1 | 150 | p3 | (TAC)5 | 15 | 378528 | 378542 |
| chr1 | 151 | p4 | (GCGA)3 | 12 | 381905 | 381916 |
| chr1 | 152 | p1 | (C)10 | 10 | 383501 | 383510 |
| chr1 | 153 | p1 | (A)10 | 10 | 383683 | 383692 |
| chr1 | 154 | p2 | (GA)6 | 12 | 385899 | 385910 |
| chr1 | 155 | p4 | (ATGC)4 | 16 | 386388 | 386403 |
| chr1 | 156 | p1 | (C)12 | 12 | 390227 | 390238 |
| chr1 | 157 | p4 | (CTAT)3 | 12 | 391228 | 391239 |
| chr1 | 158 | p4 | (AAGG)3 | 12 | 404537 | 404548 |
| chr1 | 159 | p5 | (TATAG)5 | 25 | 406262 | 406286 |
| chr1 | 160 | p2 | (CA)6 | 12 | 417018 | 417029 |
| chr1 | 161 | p6 | (CTCAAC)3 | 18 | 417462 | 417479 |
| chr1 | 162 | p2 | (GA)5 | 10 | 417602 | 417611 |
| chr1 | 163 | p4 | (ACTA)3 | 12 | 417611 | 417622 |
| chr1 | 164 | p1 | (A)13 | 13 | 424730 | 424742 |
| chr1 | 165 | p4 | (GAAT)3 | 12 | 425812 | 425823 |
| chr1 | 166 | p2 | (TC)7 | 14 | 426529 | 426542 |
| chr1 | 167 | p3 | (GAA)4 | 12 | 426826 | 426837 |
| chr2 | 1 | p4 | (ATCC)3 | 12 | 1024 | 1035 |
| chr2 | 2 | p4 | (CAAG)3 | 12 | 5320 | 5331 |
| chr2 | 3 | p2 | (GA)5 | 10 | 7557 | 7566 |
| chr2 | 4 | p4 | (TTCC)3 | 12 | 11283 | 11294 |
| chr2 | 5 | p4 | (AACC)3 | 12 | 13735 | 13746 |
| chr2 | 6 | p2 | (AT)7 | 14 | 15375 | 15388 |
| chr2 | 7 | p1 | (C)12 | 12 | 15777 | 15788 |
| chr2 | 8 | p4 | (CCTT)3 | 12 | 17631 | 17642 |
| chr2 | 9 | p4 | (AGAA)3 | 12 | 18424 | 18435 |
| chr2 | 10 | p4 | (AAGC)3 | 12 | 19287 | 19298 |
| chr2 | 11 | p2 | (GA)6 | 12 | 34127 | 34138 |
| chr2 | 12 | p1 | (A)10 | 10 | 36816 | 36825 |
| chr2 | 13 | p5 | (CTTTG)3 | 15 | 41413 | 41427 |
| chr2 | 14 | p4 | (TAAG)3 | 12 | 41433 | 41444 |
| chr2 | 15 | p3 | (CTA)4 | 12 | 42389 | 42400 |
| chr2 | 16 | p4 | (AACG)3 | 12 | 47289 | 47300 |
| chr2 | 17 | p3 | (GAT)4 | 12 | 49066 | 49077 |
| chr2 | 18 | p4 | (ATAG)3 | 12 | 50890 | 50901 |
| chr2 | 19 | p4 | (TTCT)3 | 12 | 52962 | 52973 |
| chr2 | 20 | p3 | (TTC)4 | 12 | 53525 | 53536 |
| chr2 | 21 | p3 | (CAA)4 | 12 | 59839 | 59850 |
| chr2 | 22 | p1 | (G)12 | 12 | 61050 | 61061 |
| chr2 | 23 | p3 | (ACC)4 | 12 | 61339 | 61350 |
| chr2 | 24 | p2 | (AG)5 | 10 | 65035 | 65044 |
| chr2 | 25 | p4 | (TTCT)3 | 12 | 69511 | 69522 |
| chr2 | 26 | p3 | (ATT)4 | 12 | 70787 | 70798 |
| chr2 | 27 | p1 | (T)10 | 10 | 73063 | 73072 |
| chr2 | 28 | p1 | (T)13 | 13 | 86122 | 86134 |
| chr2 | 29 | p4 | (ATAA)4 | 16 | 91401 | 91416 |
| chr2 | 30 | p4 | (CAGT)3 | 12 | 97371 | 97382 |
| chr2 | 31 | p4 | (ATCG)3 | 12 | 98872 | 98883 |
| chr2 | 32 | p4 | (AAAG)3 | 12 | 99915 | 99926 |
| chr2 | 33 | p4 | (TTAA)4 | 16 | 99996 | 100011 |
| chr2 | 34 | p1 | (T)11 | 11 | 100590 | 100600 |
| chr2 | 35 | p1 | (G)11 | 11 | 100735 | 100745 |
| chr2 | 36 | p1 | (T)10 | 10 | 101449 | 101458 |
| chr2 | 37 | p2 | (TA)6 | 12 | 103311 | 103322 |
| chr2 | 38 | p4 | (GAGC)3 | 12 | 109198 | 109209 |
| chr2 | 39 | p4 | (GCTA)3 | 12 | 110052 | 110063 |
| chr2 | 40 | p4 | (GGCC)3 | 12 | 112483 | 112494 |
| chr2 | 41 | p1 | (A)11 | 11 | 115686 | 115696 |
| chr2 | 42 | p4 | (AATA)3 | 12 | 116166 | 116177 |
| chr2 | 43 | p1 | (A)10 | 10 | 116193 | 116202 |
| chr2 | 44 | p4 | (CATT)3 | 12 | 125580 | 125591 |

Note: p1: one-base repat; p2: two-base repeat; p3: three-base repeat; p4: four-base repeat; p5: five-base repeat; p6: six-base repeat.

**Supplementary table 6. Dispersed repeat sequences** **in *Magnolia kwangsiensis* mitochondrial genome**

| **#Chr1** | **Chr2** | **type** | **alignment length** | **similarity** | **start1** | **end1** | **start2** | **end2** | **evalue** |
| --- | --- | --- | --- | --- | --- | --- | --- | --- | --- |
| chr1 | chr1 | F | 8865 | 100 | 419585 | 428449 | 216658 | 225522 | 0 |
| chr1 | chr1 | P | 1242 | 99.839 | 239206 | 240446 | 224281 | 225522 | 0 |
| chr1 | chr1 | P | 1242 | 99.839 | 427208 | 428449 | 239206 | 240446 | 0 |
| chr1 | chr1 | P | 840 | 96.19 | 320003 | 320827 | 45387 | 46219 | 0 |
| chr1 | chr1 | F | 658 | 100 | 241686 | 242343 | 126256 | 126913 | 0 |
| chr1 | chr1 | F | 615 | 100 | 325126 | 325740 | 82259 | 82873 | 0 |
| chr1 | chr1 | F | 664 | 97.289 | 67565 | 68228 | 27844 | 28507 | 0 |
| chr1 | chr1 | F | 518 | 99.035 | 164355 | 164872 | 85921 | 86437 | 0 |
| chr1 | chr1 | P | 511 | 86.693 | 90578 | 91069 | 84552 | 85041 | 3.14E-149 |
| chr1 | chr1 | F | 284 | 100 | 294083 | 294366 | 44827 | 45110 | 1.46E-147 |
| chr1 | chr1 | P | 270 | 98.148 | 45106 | 45373 | 13835 | 14104 | 6.94E-131 |
| chr1 | chr1 | P | 254 | 100 | 13851 | 14104 | 45106 | 45359 | 6.94E-131 |
| chr1 | chr1 | P | 247 | 100 | 239438 | 239684 | 28506 | 28752 | 5.40E-127 |
| chr1 | chr1 | F | 247 | 100 | 225044 | 225290 | 28506 | 28752 | 5.40E-127 |
| chr1 | chr1 | F | 247 | 100 | 427971 | 428217 | 28506 | 28752 | 5.40E-127 |
| chr1 | chr1 | F | 556 | 81.655 | 238092 | 238641 | 91227 | 91757 | 9.10E-120 |
| chr1 | chr1 | F | 182 | 99.451 | 368223 | 368404 | 22288 | 22469 | 3.42E-89 |
| chr1 | chr1 | F | 183 | 99.454 | 391797 | 391979 | 365595 | 365776 | 3.42E-89 |
| chr1 | chr1 | P | 183 | 97.268 | 319781 | 319963 | 147708 | 147890 | 4.45E-83 |
| chr1 | chr1 | P | 147 | 98.639 | 242728 | 242874 | 211539 | 211685 | 4.55E-68 |
| chr1 | chr1 | P | 141 | 100 | 211545 | 211685 | 242728 | 242868 | 4.55E-68 |
| chr1 | chr1 | F | 126 | 100 | 305037 | 305162 | 255046 | 255171 | 9.91E-60 |
| chr1 | chr1 | P | 120 | 100 | 146935 | 147054 | 66059 | 66178 | 2.15E-56 |
| chr1 | chr1 | P | 114 | 100 | 106734 | 106847 | 68895 | 69008 | 4.64E-53 |
| chr1 | chr1 | F | 112 | 100 | 254884 | 254995 | 43734 | 43845 | 6.01E-52 |
| chr1 | chr1 | P | 104 | 100 | 302891 | 302994 | 134051 | 134154 | 1.68E-47 |
| chr1 | chr1 | P | 108 | 98.148 | 26272 | 26379 | 22370 | 22477 | 2.18E-46 |
| chr1 | chr1 | P | 105 | 99.048 | 39179 | 39283 | 25927 | 26031 | 2.18E-46 |
| chr1 | chr1 | P | 102 | 100 | 25930 | 26031 | 39179 | 39280 | 2.18E-46 |
| chr1 | chr1 | P | 100 | 100 | 300162 | 300261 | 101293 | 101392 | 2.81E-45 |
| chr1 | chr1 | P | 114 | 93.86 | 368305 | 368418 | 26266 | 26379 | 2.19E-41 |
| chr1 | chr1 | P | 111 | 94.595 | 26269 | 26379 | 368305 | 368415 | 2.19E-41 |
| chr1 | chr1 | P | 92 | 100 | 115313 | 115404 | 45353 | 45444 | 7.88E-41 |
| chr1 | chr1 | P | 119 | 92.437 | 211544 | 211661 | 51209 | 51326 | 2.83E-40 |
| chr1 | chr1 | F | 118 | 92.373 | 242752 | 242868 | 51209 | 51325 | 1.02E-39 |
| chr1 | chr1 | F | 111 | 93.694 | 247457 | 247566 | 201880 | 201989 | 3.67E-39 |
| chr1 | chr1 | F | 118 | 91.525 | 351600 | 351715 | 40275 | 40391 | 1.71E-37 |
| chr1 | chr1 | P | 88 | 98.864 | 313549 | 313636 | 26317 | 26404 | 6.14E-37 |
| chr1 | chr1 | P | 92 | 97.826 | 337769 | 337860 | 223016 | 223106 | 6.14E-37 |
| chr1 | chr1 | P | 89 | 98.876 | 425946 | 426033 | 337769 | 337857 | 6.14E-37 |
| chr1 | chr1 | P | 89 | 98.876 | 223019 | 223106 | 337769 | 337857 | 6.14E-37 |
| chr1 | chr1 | P | 92 | 97.826 | 337769 | 337860 | 425943 | 426033 | 6.14E-37 |
| chr1 | chr1 | P | 96 | 95.833 | 242634 | 242729 | 116639 | 116734 | 2.21E-36 |
| chr1 | chr1 | F | 87 | 97.701 | 209376 | 209462 | 122742 | 122828 | 1.03E-34 |
| chr1 | chr1 | P | 85 | 98.824 | 337781 | 337864 | 196874 | 196958 | 1.03E-34 |
| chr1 | chr1 | P | 81 | 100 | 259168 | 259248 | 206830 | 206910 | 1.03E-34 |
| chr1 | chr1 | F | 85 | 98.824 | 406053 | 406137 | 337783 | 337866 | 1.03E-34 |
| chr1 | chr1 | P | 82 | 98.78 | 384315 | 384396 | 165495 | 165576 | 1.33E-33 |
| chr1 | chr1 | P | 87 | 96.552 | 406053 | 406138 | 196871 | 196956 | 1.72E-32 |
| chr1 | chr1 | P | 84 | 97.619 | 196874 | 196956 | 406053 | 406135 | 1.72E-32 |
| chr1 | chr1 | P | 76 | 100 | 226442 | 226517 | 171545 | 171620 | 6.18E-32 |
| chr1 | chr1 | F | 94 | 92.553 | 123088 | 123181 | 41494 | 41587 | 2.88E-30 |
| chr1 | chr1 | F | 98 | 91.837 | 223016 | 223110 | 196878 | 196974 | 1.03E-29 |
| chr1 | chr1 | F | 98 | 91.837 | 425943 | 426037 | 196878 | 196974 | 1.03E-29 |
| chr1 | chr1 | P | 86 | 94.186 | 265004 | 265088 | 231249 | 231334 | 1.34E-28 |
| chr1 | chr1 | P | 83 | 95.181 | 231252 | 231334 | 265004 | 265085 | 1.34E-28 |
| chr1 | chr1 | P | 79 | 96.203 | 406053 | 406131 | 223016 | 223092 | 4.81E-28 |
| chr1 | chr1 | P | 76 | 97.368 | 425946 | 426019 | 406053 | 406128 | 4.81E-28 |
| chr1 | chr1 | P | 76 | 97.368 | 223019 | 223092 | 406053 | 406128 | 4.81E-28 |
| chr1 | chr1 | P | 79 | 96.203 | 406053 | 406131 | 425943 | 426019 | 4.81E-28 |
| chr1 | chr1 | F | 141 | 84.397 | 113767 | 113907 | 85316 | 85443 | 1.73E-27 |
| chr1 | chr1 | F | 80 | 95 | 350661 | 350740 | 188615 | 188694 | 1.73E-27 |
| chr1 | chr1 | P | 71 | 98.592 | 360925 | 360995 | 231250 | 231320 | 1.73E-27 |
| chr1 | chr1 | F | 68 | 100 | 360925 | 360992 | 265017 | 265084 | 1.73E-27 |
| chr1 | chr1 | P | 68 | 100 | 231253 | 231320 | 360925 | 360992 | 1.73E-27 |
| chr1 | chr1 | P | 76 | 96.053 | 258683 | 258758 | 222065 | 222140 | 6.22E-27 |
| chr1 | chr1 | P | 98 | 89.796 | 352628 | 352725 | 235446 | 235542 | 6.22E-27 |
| chr1 | chr1 | P | 76 | 96.053 | 424992 | 425067 | 258683 | 258758 | 6.22E-27 |
| chr1 | chr1 | F | 75 | 96 | 357005 | 357079 | 22438 | 22512 | 2.24E-26 |
| chr1 | chr1 | F | 66 | 100 | 321132 | 321197 | 230093 | 230158 | 2.24E-26 |
| chr1 | chr1 | F | 66 | 100 | 247160 | 247225 | 243787 | 243852 | 2.24E-26 |
| chr1 | chr1 | P | 67 | 98.507 | 114108 | 114174 | 41582 | 41648 | 2.90E-25 |
| chr1 | chr1 | P | 70 | 97.143 | 313551 | 313620 | 69095 | 69164 | 2.90E-25 |
| chr1 | chr1 | P | 64 | 100 | 276280 | 276343 | 126089 | 126152 | 2.90E-25 |
| chr1 | chr1 | P | 64 | 100 | 313551 | 313614 | 245456 | 245519 | 2.90E-25 |
| chr1 | chr1 | F | 63 | 100 | 313574 | 313636 | 22370 | 22432 | 1.04E-24 |
| chr1 | chr1 | F | 98 | 88.776 | 304313 | 304408 | 41475 | 41570 | 1.04E-24 |
| chr1 | chr1 | F | 63 | 100 | 368305 | 368367 | 313574 | 313636 | 1.04E-24 |
| chr1 | chr1 | F | 68 | 97.059 | 69095 | 69162 | 26333 | 26400 | 3.75E-24 |
| chr1 | chr1 | F | 62 | 100 | 245456 | 245517 | 26339 | 26400 | 3.75E-24 |
| chr1 | chr1 | F | 68 | 97.059 | 245456 | 245523 | 69101 | 69168 | 3.75E-24 |
| chr1 | chr1 | F | 74 | 94.595 | 415773 | 415846 | 118941 | 119014 | 3.75E-24 |
| chr1 | chr1 | F | 68 | 97.059 | 368222 | 368289 | 126087 | 126154 | 3.75E-24 |
| chr1 | chr1 | F | 75 | 94.667 | 118941 | 119013 | 415773 | 415847 | 3.75E-24 |
| chr1 | chr1 | P | 64 | 98.438 | 372348 | 372411 | 3761 | 3824 | 1.35E-23 |
| chr1 | chr1 | F | 67 | 97.015 | 126088 | 126154 | 22288 | 22354 | 1.35E-23 |
| chr1 | chr1 | F | 63 | 98.413 | 210144 | 210206 | 28750 | 28812 | 4.85E-23 |
| chr1 | chr1 | P | 76 | 93.421 | 247222 | 247295 | 126087 | 126162 | 4.85E-23 |
| chr1 | chr1 | P | 59 | 100 | 276280 | 276338 | 22294 | 22352 | 1.74E-22 |
| chr1 | chr1 | P | 62 | 98.387 | 350679 | 350740 | 41475 | 41536 | 1.74E-22 |
| chr1 | chr1 | P | 101 | 86.139 | 297394 | 297494 | 47784 | 47884 | 1.74E-22 |
| chr1 | chr1 | F | 65 | 96.923 | 126098 | 126162 | 69004 | 69068 | 1.74E-22 |
| chr1 | chr1 | P | 65 | 96.923 | 304310 | 304374 | 188633 | 188697 | 1.74E-22 |
| chr1 | chr1 | P | 75 | 93.333 | 304192 | 304266 | 223207 | 223280 | 1.74E-22 |
| chr1 | chr1 | P | 59 | 100 | 368229 | 368287 | 276280 | 276338 | 1.74E-22 |
| chr1 | chr1 | P | 98 | 86.735 | 47787 | 47884 | 297394 | 297491 | 1.74E-22 |
| chr1 | chr1 | P | 75 | 93.333 | 426134 | 426207 | 304192 | 304266 | 1.74E-22 |
| chr1 | chr1 | P | 77 | 92.208 | 350656 | 350731 | 41573 | 41648 | 6.27E-22 |
| chr1 | chr1 | F | 67 | 95.522 | 350656 | 350722 | 114108 | 114174 | 6.27E-22 |
| chr1 | chr1 | F | 58 | 100 | 320770 | 320827 | 115313 | 115370 | 6.27E-22 |
| chr1 | chr1 | F | 72 | 93.056 | 346975 | 347046 | 3762 | 3833 | 2.25E-21 |
| chr1 | chr1 | P | 57 | 100 | 227872 | 227928 | 87634 | 87690 | 2.25E-21 |
| chr1 | chr1 | F | 73 | 93.151 | 206343 | 206413 | 87789 | 87861 | 2.25E-21 |
| chr1 | chr1 | F | 81 | 91.358 | 243802 | 243880 | 123087 | 123163 | 2.25E-21 |
| chr1 | chr1 | F | 64 | 96.875 | 223154 | 223217 | 144599 | 144661 | 2.25E-21 |
| chr1 | chr1 | F | 64 | 96.875 | 426081 | 426144 | 144599 | 144661 | 2.25E-21 |
| chr1 | chr1 | P | 96 | 87.5 | 295675 | 295765 | 226534 | 226629 | 2.25E-21 |
| chr1 | chr1 | F | 67 | 95.522 | 337567 | 337630 | 26317 | 26383 | 8.11E-21 |
| chr1 | chr1 | P | 66 | 95.455 | 188621 | 188685 | 41573 | 41637 | 8.11E-21 |
| chr1 | chr1 | F | 133 | 81.203 | 137221 | 137353 | 89820 | 89949 | 8.11E-21 |
| chr1 | chr1 | F | 62 | 96.774 | 188615 | 188676 | 114113 | 114174 | 8.11E-21 |
| chr1 | chr1 | P | 69 | 94.203 | 170160 | 170228 | 117370 | 117437 | 8.11E-21 |
| chr1 | chr1 | F | 79 | 91.139 | 304332 | 304408 | 123088 | 123164 | 8.11E-21 |
| chr1 | chr1 | P | 72 | 93.056 | 117370 | 117440 | 170157 | 170228 | 8.11E-21 |
| chr1 | chr1 | P | 72 | 93.056 | 41573 | 41643 | 188615 | 188685 | 8.11E-21 |
| chr1 | chr1 | P | 65 | 95.385 | 243722 | 243786 | 201896 | 201960 | 8.11E-21 |
| chr1 | chr1 | P | 56 | 100 | 350667 | 350722 | 205941 | 205996 | 8.11E-21 |
| chr1 | chr1 | P | 67 | 95.522 | 337567 | 337630 | 313570 | 313636 | 8.11E-21 |
| chr1 | chr1 | P | 70 | 94.286 | 313570 | 313639 | 337564 | 337630 | 8.11E-21 |
| chr1 | chr1 | P | 68 | 94.118 | 372347 | 372414 | 368523 | 368590 | 8.11E-21 |
| chr1 | chr1 | F | 64 | 95.312 | 368526 | 368589 | 3761 | 3824 | 2.92E-20 |
| chr1 | chr1 | P | 71 | 92.958 | 247216 | 247284 | 69004 | 69074 | 2.92E-20 |
| chr1 | chr1 | P | 55 | 100 | 201953 | 202007 | 75892 | 75946 | 2.92E-20 |
| chr1 | chr1 | P | 65 | 95.385 | 205933 | 205996 | 114119 | 114183 | 2.92E-20 |
| chr1 | chr1 | P | 61 | 96.721 | 205941 | 206001 | 188616 | 188676 | 2.92E-20 |
| chr1 | chr1 | P | 87 | 88.506 | 337528 | 337613 | 205958 | 206042 | 2.92E-20 |
| chr1 | chr1 | P | 55 | 100 | 368212 | 368266 | 263991 | 264045 | 2.92E-20 |
| chr1 | chr1 | P | 54 | 100 | 406073 | 406126 | 206121 | 206174 | 1.05E-19 |
| chr1 | chr1 | P | 54 | 100 | 337802 | 337855 | 206121 | 206174 | 1.05E-19 |
| chr1 | chr1 | P | 97 | 86.598 | 356909 | 357000 | 247218 | 247312 | 1.05E-19 |
| chr1 | chr1 | P | 81 | 90.123 | 337567 | 337644 | 22356 | 22432 | 3.77E-19 |
| chr1 | chr1 | P | 62 | 95.161 | 188633 | 188694 | 41475 | 41536 | 3.77E-19 |
| chr1 | chr1 | P | 75 | 90.667 | 368215 | 368289 | 247230 | 247302 | 3.77E-19 |
| chr1 | chr1 | P | 62 | 95.161 | 350679 | 350740 | 304313 | 304374 | 3.77E-19 |
| chr1 | chr1 | P | 81 | 90.123 | 368291 | 368367 | 337567 | 337644 | 3.77E-19 |
| chr1 | chr1 | F | 53 | 100 | 375552 | 375604 | 346909 | 346961 | 3.77E-19 |
| chr1 | chr1 | P | 63 | 95.238 | 372349 | 372410 | 346974 | 347036 | 3.77E-19 |
| chr1 | chr1 | F | 55 | 98.182 | 69004 | 69058 | 22298 | 22352 | 1.36E-18 |
| chr1 | chr1 | F | 86 | 88.372 | 243792 | 243874 | 41483 | 41563 | 1.36E-18 |
| chr1 | chr1 | P | 55 | 98.182 | 276280 | 276334 | 69004 | 69058 | 1.36E-18 |
| chr1 | chr1 | F | 55 | 98.182 | 368233 | 368287 | 69004 | 69058 | 1.36E-18 |
| chr1 | chr1 | F | 52 | 100 | 263951 | 264002 | 106844 | 106895 | 1.36E-18 |
| chr1 | chr1 | F | 75 | 89.333 | 66070 | 66144 | 15156 | 15230 | 4.88E-18 |
| chr1 | chr1 | P | 75 | 89.333 | 146969 | 147043 | 15156 | 15230 | 4.88E-18 |
| chr1 | chr1 | P | 54 | 98.148 | 205932 | 205985 | 68904 | 68957 | 4.88E-18 |
| chr1 | chr1 | F | 51 | 100 | 205932 | 205982 | 106785 | 106835 | 4.88E-18 |
| chr1 | chr1 | F | 55 | 98.182 | 206121 | 206174 | 196883 | 196937 | 4.88E-18 |
| chr1 | chr1 | P | 51 | 100 | 68907 | 68957 | 205932 | 205982 | 4.88E-18 |
| chr1 | chr1 | P | 56 | 96.429 | 46019 | 46074 | 14478 | 14533 | 1.76E-17 |
| chr1 | chr1 | F | 93 | 84.946 | 29502 | 29593 | 29225 | 29316 | 1.76E-17 |
| chr1 | chr1 | F | 54 | 98.148 | 223021 | 223073 | 206121 | 206174 | 1.76E-17 |
| chr1 | chr1 | F | 54 | 98.148 | 425948 | 426000 | 206121 | 206174 | 1.76E-17 |
| chr1 | chr1 | F | 97 | 85.567 | 304321 | 304411 | 243792 | 243884 | 1.76E-17 |
| chr1 | chr1 | P | 73 | 90.411 | 263248 | 263319 | 258725 | 258795 | 1.76E-17 |
| chr1 | chr1 | F | 49 | 100 | 207380 | 207428 | 26021 | 26069 | 6.31E-17 |
| chr1 | chr1 | P | 94 | 84.043 | 135235 | 135328 | 90122 | 90215 | 6.31E-17 |
| chr1 | chr1 | P | 91 | 84.615 | 90125 | 90215 | 135235 | 135325 | 6.31E-17 |
| chr1 | chr1 | P | 58 | 94.828 | 247480 | 247537 | 243722 | 243779 | 6.31E-17 |
| chr1 | chr1 | P | 72 | 90.278 | 36990 | 37061 | 20224 | 20289 | 2.27E-16 |
| chr1 | chr1 | F | 55 | 96.364 | 245495 | 245548 | 123177 | 123230 | 2.27E-16 |
| chr1 | chr1 | F | 63 | 92.063 | 356981 | 357043 | 140222 | 140284 | 2.27E-16 |
| chr1 | chr1 | P | 48 | 100 | 141202 | 141249 | 141202 | 141249 | 2.27E-16 |
| chr1 | chr1 | F | 64 | 92.188 | 276280 | 276343 | 247232 | 247293 | 2.27E-16 |
| chr1 | chr1 | P | 48 | 100 | 313542 | 313589 | 304371 | 304418 | 2.27E-16 |
| chr1 | chr1 | P | 60 | 93.333 | 415724 | 415783 | 356977 | 357036 | 2.27E-16 |
| chr1 | chr1 | P | 66 | 90.909 | 356977 | 357042 | 415718 | 415783 | 2.27E-16 |
| chr1 | chr1 | F | 56 | 94.643 | 320134 | 320189 | 14478 | 14533 | 8.17E-16 |
| chr1 | chr1 | P | 47 | 100 | 30504 | 30550 | 26272 | 26318 | 8.17E-16 |
| chr1 | chr1 | P | 54 | 96.296 | 26272 | 26323 | 30497 | 30550 | 8.17E-16 |
| chr1 | chr1 | F | 56 | 94.643 | 205941 | 205996 | 41582 | 41637 | 8.17E-16 |
| chr1 | chr1 | P | 71 | 88.732 | 297953 | 298023 | 47251 | 47321 | 8.17E-16 |
| chr1 | chr1 | F | 47 | 100 | 201847 | 201893 | 114010 | 114056 | 8.17E-16 |
| chr1 | chr1 | P | 63 | 92.063 | 123069 | 123130 | 114131 | 114192 | 8.17E-16 |
| chr1 | chr1 | F | 47 | 100 | 243909 | 243955 | 120898 | 120944 | 8.17E-16 |
| chr1 | chr1 | P | 50 | 98 | 226507 | 226556 | 138837 | 138886 | 8.17E-16 |
| chr1 | chr1 | P | 47 | 100 | 426134 | 426180 | 221967 | 222013 | 8.17E-16 |
| chr1 | chr1 | P | 47 | 100 | 223207 | 223253 | 221967 | 222013 | 8.17E-16 |
| chr1 | chr1 | P | 47 | 100 | 424894 | 424940 | 223207 | 223253 | 8.17E-16 |
| chr1 | chr1 | F | 75 | 88 | 368527 | 368600 | 346975 | 347049 | 8.17E-16 |
| chr1 | chr1 | P | 47 | 100 | 426134 | 426180 | 424894 | 424940 | 8.17E-16 |
| chr1 | chr1 | P | 52 | 96.154 | 258980 | 259031 | 64909 | 64960 | 2.94E-15 |
| chr1 | chr1 | F | 46 | 100 | 229553 | 229598 | 68886 | 68931 | 2.94E-15 |
| chr1 | chr1 | F | 74 | 89.189 | 356930 | 356996 | 126089 | 126162 | 2.94E-15 |
| chr1 | chr1 | F | 49 | 97.959 | 304220 | 304268 | 221967 | 222015 | 2.94E-15 |
| chr1 | chr1 | P | 66 | 90.909 | 304448 | 304512 | 243694 | 243756 | 2.94E-15 |
| chr1 | chr1 | P | 92 | 84.783 | 368329 | 368419 | 247150 | 247234 | 2.94E-15 |
| chr1 | chr1 | P | 49 | 97.959 | 64912 | 64960 | 258980 | 259028 | 2.94E-15 |
| chr1 | chr1 | F | 49 | 97.959 | 424894 | 424942 | 304220 | 304268 | 2.94E-15 |
| chr1 | chr1 | P | 67 | 89.552 | 247230 | 247294 | 22288 | 22354 | 1.06E-14 |
| chr1 | chr1 | F | 74 | 87.838 | 209437 | 209510 | 122834 | 122904 | 1.06E-14 |
| chr1 | chr1 | F | 77 | 87.013 | 247105 | 247179 | 229474 | 229548 | 1.06E-14 |
| chr1 | chr1 | P | 61 | 91.803 | 22294 | 22354 | 247230 | 247288 | 1.06E-14 |
| chr1 | chr1 | P | 45 | 100 | 406467 | 406511 | 399419 | 399463 | 1.06E-14 |
| chr1 | chr1 | P | 44 | 100 | 263991 | 264034 | 22288 | 22331 | 3.80E-14 |
| chr1 | chr1 | F | 59 | 91.525 | 356986 | 357044 | 30492 | 30550 | 3.80E-14 |
| chr1 | chr1 | F | 50 | 96 | 337811 | 337860 | 263465 | 263514 | 3.80E-14 |
| chr1 | chr1 | F | 50 | 96 | 406082 | 406131 | 263465 | 263514 | 3.80E-14 |
| chr1 | chr1 | P | 101 | 83.168 | 258903 | 258995 | 22284 | 22379 | 1.37E-13 |
| chr1 | chr1 | P | 83 | 85.542 | 247159 | 247234 | 22394 | 22475 | 1.37E-13 |
| chr1 | chr1 | P | 49 | 95.918 | 356998 | 357046 | 26270 | 26318 | 1.37E-13 |
| chr1 | chr1 | F | 49 | 95.918 | 41565 | 41613 | 26273 | 26321 | 1.37E-13 |
| chr1 | chr1 | F | 108 | 81.481 | 29700 | 29805 | 29406 | 29506 | 1.37E-13 |
| chr1 | chr1 | F | 50 | 96 | 113895 | 113944 | 35075 | 35123 | 1.37E-13 |
| chr1 | chr1 | F | 74 | 86.486 | 222034 | 222107 | 118929 | 119001 | 1.37E-13 |
| chr1 | chr1 | F | 74 | 86.486 | 424961 | 425034 | 118929 | 119001 | 1.37E-13 |
| chr1 | chr1 | P | 43 | 100 | 188633 | 188675 | 123088 | 123130 | 1.37E-13 |
| chr1 | chr1 | F | 57 | 92.982 | 267771 | 267827 | 205915 | 205968 | 1.37E-13 |
| chr1 | chr1 | P | 98 | 83.673 | 368222 | 368314 | 258903 | 258992 | 1.37E-13 |
| chr1 | chr1 | F | 46 | 97.826 | 337641 | 337686 | 304314 | 304359 | 1.37E-13 |
| chr1 | chr1 | P | 94 | 82.979 | 350659 | 350747 | 313544 | 313636 | 1.37E-13 |
| chr1 | chr1 | P | 69 | 86.957 | 270786 | 270854 | 43357 | 43425 | 4.91E-13 |
| chr1 | chr1 | F | 45 | 97.778 | 350678 | 350722 | 68904 | 68948 | 4.91E-13 |
| chr1 | chr1 | F | 42 | 100 | 415676 | 415717 | 75894 | 75935 | 4.91E-13 |
| chr1 | chr1 | P | 42 | 100 | 350681 | 350722 | 106794 | 106835 | 4.91E-13 |
| chr1 | chr1 | P | 42 | 100 | 415676 | 415717 | 201964 | 202005 | 4.91E-13 |
| chr1 | chr1 | P | 45 | 97.778 | 263465 | 263509 | 206121 | 206165 | 4.91E-13 |
| chr1 | chr1 | F | 54 | 92.593 | 310363 | 310416 | 221567 | 221620 | 4.91E-13 |
| chr1 | chr1 | P | 45 | 97.778 | 247552 | 247596 | 243692 | 243736 | 4.91E-13 |
| chr1 | chr1 | P | 42 | 100 | 243695 | 243736 | 247552 | 247593 | 4.91E-13 |
| chr1 | chr1 | P | 147 | 76.871 | 298254 | 298397 | 267839 | 267983 | 4.91E-13 |
| chr1 | chr1 | F | 54 | 92.593 | 424494 | 424547 | 310363 | 310416 | 4.91E-13 |
| chr1 | chr1 | P | 45 | 97.778 | 106794 | 106838 | 350678 | 350722 | 4.91E-13 |
| chr1 | chr1 | P | 47 | 95.745 | 69095 | 69141 | 22370 | 22416 | 1.77E-12 |
| chr1 | chr1 | P | 41 | 100 | 245456 | 245496 | 22370 | 22410 | 1.77E-12 |
| chr1 | chr1 | F | 54 | 92.593 | 30497 | 30550 | 22426 | 22477 | 1.77E-12 |
| chr1 | chr1 | P | 60 | 90 | 164644 | 164701 | 27755 | 27814 | 1.77E-12 |
| chr1 | chr1 | P | 60 | 90 | 86209 | 86266 | 27755 | 27814 | 1.77E-12 |
| chr1 | chr1 | P | 53 | 92.453 | 41558 | 41610 | 30504 | 30556 | 1.77E-12 |
| chr1 | chr1 | P | 129 | 79.07 | 291320 | 291445 | 40378 | 40495 | 1.77E-12 |
| chr1 | chr1 | F | 54 | 92.593 | 41573 | 41625 | 41484 | 41536 | 1.77E-12 |
| chr1 | chr1 | F | 44 | 97.727 | 205941 | 205984 | 41493 | 41536 | 1.77E-12 |
| chr1 | chr1 | F | 41 | 100 | 356960 | 357000 | 41583 | 41623 | 1.77E-12 |
| chr1 | chr1 | P | 52 | 94.231 | 123155 | 123205 | 43644 | 43693 | 1.77E-12 |
| chr1 | chr1 | F | 54 | 92.593 | 114130 | 114183 | 68904 | 68956 | 1.77E-12 |
| chr1 | chr1 | F | 69 | 88.406 | 356939 | 357000 | 69004 | 69072 | 1.77E-12 |
| chr1 | chr1 | P | 53 | 92.453 | 188616 | 188668 | 69039 | 69091 | 1.77E-12 |
| chr1 | chr1 | P | 47 | 95.745 | 368305 | 368351 | 69095 | 69141 | 1.77E-12 |
| chr1 | chr1 | P | 50 | 94 | 27765 | 27814 | 86209 | 86258 | 1.77E-12 |
| chr1 | chr1 | P | 47 | 95.745 | 134254 | 134300 | 100172 | 100218 | 1.77E-12 |
| chr1 | chr1 | P | 51 | 94.118 | 114133 | 114183 | 106786 | 106835 | 1.77E-12 |
| chr1 | chr1 | P | 54 | 92.593 | 106786 | 106838 | 114130 | 114183 | 1.77E-12 |
| chr1 | chr1 | F | 51 | 94.118 | 356951 | 357000 | 123078 | 123128 | 1.77E-12 |
| chr1 | chr1 | F | 58 | 91.379 | 137965 | 138020 | 136480 | 136535 | 1.77E-12 |
| chr1 | chr1 | P | 50 | 94 | 27765 | 27814 | 164644 | 164693 | 1.77E-12 |
| chr1 | chr1 | F | 41 | 100 | 192788 | 192828 | 171746 | 171786 | 1.77E-12 |
| chr1 | chr1 | P | 51 | 94.118 | 263465 | 263514 | 196878 | 196928 | 1.77E-12 |
| chr1 | chr1 | P | 41 | 100 | 368305 | 368345 | 245456 | 245496 | 1.77E-12 |
| chr1 | chr1 | F | 44 | 97.727 | 337545 | 337588 | 304291 | 304334 | 1.77E-12 |
| chr1 | chr1 | P | 57 | 91.228 | 356988 | 357043 | 337641 | 337696 | 1.77E-12 |
| chr1 | chr1 | F | 54 | 92.593 | 368363 | 368415 | 350691 | 350743 | 1.77E-12 |
| chr1 | chr1 | P | 47 | 95.745 | 243787 | 243832 | 22428 | 22474 | 6.36E-12 |
| chr1 | chr1 | P | 56 | 91.071 | 229522 | 229576 | 26352 | 26406 | 6.36E-12 |
| chr1 | chr1 | F | 63 | 88.889 | 247159 | 247221 | 41566 | 41625 | 6.36E-12 |
| chr1 | chr1 | F | 43 | 97.674 | 123088 | 123130 | 41583 | 41625 | 6.36E-12 |
| chr1 | chr1 | F | 40 | 100 | 126086 | 126125 | 68851 | 68890 | 6.36E-12 |
| chr1 | chr1 | P | 137 | 76.642 | 300024 | 300159 | 74538 | 74673 | 6.36E-12 |
| chr1 | chr1 | F | 71 | 87.324 | 170166 | 170230 | 101227 | 101296 | 6.36E-12 |
| chr1 | chr1 | P | 69 | 86.957 | 117366 | 117431 | 101227 | 101295 | 6.36E-12 |
| chr1 | chr1 | F | 62 | 88.71 | 125915 | 125976 | 122593 | 122652 | 6.36E-12 |
| chr1 | chr1 | F | 40 | 100 | 415791 | 415830 | 222065 | 222104 | 6.36E-12 |
| chr1 | chr1 | P | 46 | 95.652 | 415717 | 415762 | 368366 | 368411 | 6.36E-12 |
| chr1 | chr1 | F | 40 | 100 | 424992 | 425031 | 415791 | 415830 | 6.36E-12 |
| chr1 | chr1 | F | 99 | 80.808 | 350649 | 350742 | 26307 | 26404 | 2.29E-11 |
| chr1 | chr1 | P | 42 | 97.619 | 355684 | 355725 | 27812 | 27853 | 2.29E-11 |
| chr1 | chr1 | F | 39 | 100 | 34974 | 35012 | 34934 | 34972 | 2.29E-11 |
| chr1 | chr1 | F | 42 | 97.619 | 106794 | 106835 | 41493 | 41534 | 2.29E-11 |
| chr1 | chr1 | P | 42 | 97.619 | 68907 | 68948 | 41493 | 41534 | 2.29E-11 |
| chr1 | chr1 | P | 96 | 81.25 | 337569 | 337660 | 41549 | 41643 | 2.29E-11 |
| chr1 | chr1 | P | 42 | 97.619 | 140247 | 140288 | 41561 | 41602 | 2.29E-11 |
| chr1 | chr1 | F | 62 | 88.71 | 243787 | 243848 | 41567 | 41625 | 2.29E-11 |
| chr1 | chr1 | F | 48 | 93.75 | 69039 | 69086 | 41590 | 41637 | 2.29E-11 |
| chr1 | chr1 | F | 39 | 100 | 304371 | 304409 | 69126 | 69164 | 2.29E-11 |
| chr1 | chr1 | P | 63 | 88.889 | 337690 | 337748 | 114055 | 114115 | 2.29E-11 |
| chr1 | chr1 | F | 46 | 95.652 | 337569 | 337613 | 114113 | 114157 | 2.29E-11 |
| chr1 | chr1 | P | 45 | 95.556 | 263991 | 264035 | 126087 | 126131 | 2.29E-11 |
| chr1 | chr1 | P | 39 | 100 | 368222 | 368260 | 176590 | 176628 | 2.29E-11 |
| chr1 | chr1 | F | 39 | 100 | 263997 | 264035 | 176590 | 176628 | 2.29E-11 |
| chr1 | chr1 | F | 42 | 97.619 | 415735 | 415776 | 202142 | 202183 | 2.29E-11 |
| chr1 | chr1 | P | 46 | 95.652 | 263465 | 263510 | 223020 | 223064 | 2.29E-11 |
| chr1 | chr1 | P | 73 | 86.301 | 350679 | 350747 | 243779 | 243848 | 2.29E-11 |
| chr1 | chr1 | F | 39 | 100 | 304371 | 304409 | 245481 | 245519 | 2.29E-11 |
| chr1 | chr1 | P | 46 | 95.652 | 425947 | 425991 | 263465 | 263510 | 2.29E-11 |
| chr1 | chr1 | P | 109 | 78.899 | 292317 | 292424 | 292317 | 292424 | 2.29E-11 |
| chr1 | chr1 | P | 86 | 82.558 | 41559 | 41643 | 337569 | 337652 | 2.29E-11 |
| chr1 | chr1 | P | 45 | 95.556 | 27812 | 27856 | 355681 | 355725 | 2.29E-11 |
| chr1 | chr1 | F | 45 | 95.556 | 405270 | 405314 | 405226 | 405270 | 2.29E-11 |
| chr1 | chr1 | P | 38 | 100 | 176590 | 176627 | 22288 | 22325 | 8.22E-11 |
| chr1 | chr1 | F | 60 | 88.333 | 188645 | 188703 | 22428 | 22486 | 8.22E-11 |
| chr1 | chr1 | F | 51 | 92.157 | 350691 | 350740 | 22428 | 22477 | 8.22E-11 |
| chr1 | chr1 | F | 87 | 82.759 | 247155 | 247234 | 26270 | 26355 | 8.22E-11 |
| chr1 | chr1 | P | 51 | 92.157 | 188645 | 188694 | 26272 | 26321 | 8.22E-11 |
| chr1 | chr1 | F | 65 | 87.692 | 114097 | 114157 | 26304 | 26366 | 8.22E-11 |
| chr1 | chr1 | F | 41 | 97.561 | 304371 | 304411 | 26364 | 26404 | 8.22E-11 |
| chr1 | chr1 | F | 68 | 86.765 | 247159 | 247225 | 41477 | 41540 | 8.22E-11 |
| chr1 | chr1 | F | 79 | 83.544 | 337611 | 337686 | 41533 | 41610 | 8.22E-11 |
| chr1 | chr1 | F | 54 | 90.741 | 304322 | 304374 | 41573 | 41625 | 8.22E-11 |
| chr1 | chr1 | P | 44 | 95.455 | 426207 | 426250 | 42088 | 42131 | 8.22E-11 |
| chr1 | chr1 | P | 44 | 95.455 | 223280 | 223323 | 42088 | 42131 | 8.22E-11 |
| chr1 | chr1 | P | 38 | 100 | 69140 | 69177 | 43635 | 43672 | 8.22E-11 |
| chr1 | chr1 | F | 69 | 85.507 | 202079 | 202146 | 64909 | 64976 | 8.22E-11 |
| chr1 | chr1 | F | 38 | 100 | 368373 | 368410 | 106874 | 106911 | 8.22E-11 |
| chr1 | chr1 | P | 44 | 95.455 | 304331 | 304374 | 114131 | 114174 | 8.22E-11 |
| chr1 | chr1 | P | 41 | 97.561 | 356960 | 357000 | 114133 | 114173 | 8.22E-11 |
| chr1 | chr1 | F | 66 | 86.364 | 234888 | 234952 | 117951 | 118015 | 8.22E-11 |
| chr1 | chr1 | P | 44 | 95.455 | 258716 | 258759 | 118958 | 119001 | 8.22E-11 |
| chr1 | chr1 | F | 51 | 92.157 | 258870 | 258919 | 123144 | 123193 | 8.22E-11 |
| chr1 | chr1 | P | 89 | 82.022 | 235787 | 235871 | 126707 | 126794 | 8.22E-11 |
| chr1 | chr1 | F | 45 | 95.556 | 247651 | 247695 | 166635 | 166678 | 8.22E-11 |
| chr1 | chr1 | P | 41 | 97.561 | 356960 | 357000 | 188635 | 188675 | 8.22E-11 |
| chr1 | chr1 | P | 86 | 82.558 | 242140 | 242224 | 235787 | 235868 | 8.22E-11 |
| chr1 | chr1 | P | 86 | 82.558 | 126710 | 126794 | 235787 | 235868 | 8.22E-11 |
| chr1 | chr1 | P | 89 | 82.022 | 235787 | 235871 | 242137 | 242224 | 8.22E-11 |
| chr1 | chr1 | P | 42 | 97.619 | 368363 | 368404 | 243792 | 243832 | 8.22E-11 |
| chr1 | chr1 | P | 68 | 86.765 | 350679 | 350742 | 247155 | 247221 | 8.22E-11 |
| chr1 | chr1 | F | 68 | 86.765 | 304315 | 304378 | 247159 | 247225 | 8.22E-11 |
| chr1 | chr1 | P | 48 | 93.75 | 356935 | 356980 | 263981 | 264028 | 8.22E-11 |
| chr1 | chr1 | F | 38 | 100 | 276301 | 276338 | 263991 | 264028 | 8.22E-11 |
| chr1 | chr1 | F | 47 | 93.617 | 267606 | 267652 | 267533 | 267579 | 8.22E-11 |
| chr1 | chr1 | P | 37 | 100 | 117369 | 117405 | 8686 | 8722 | 2.96E-10 |
| chr1 | chr1 | P | 49 | 91.837 | 41565 | 41613 | 22428 | 22476 | 2.96E-10 |
| chr1 | chr1 | P | 37 | 100 | 141196 | 141232 | 26370 | 26406 | 2.96E-10 |
| chr1 | chr1 | P | 37 | 100 | 276307 | 276343 | 68854 | 68890 | 2.96E-10 |
| chr1 | chr1 | F | 41 | 97.561 | 337735 | 337775 | 68884 | 68923 | 2.96E-10 |
| chr1 | chr1 | F | 51 | 92.157 | 337583 | 337630 | 69095 | 69145 | 2.96E-10 |
| chr1 | chr1 | P | 37 | 100 | 247530 | 247566 | 75910 | 75946 | 2.96E-10 |
| chr1 | chr1 | P | 37 | 100 | 229562 | 229598 | 106811 | 106847 | 2.96E-10 |
| chr1 | chr1 | P | 50 | 92 | 426022 | 426071 | 118992 | 119040 | 2.96E-10 |
| chr1 | chr1 | P | 50 | 92 | 223095 | 223144 | 118992 | 119040 | 2.96E-10 |
| chr1 | chr1 | F | 51 | 92.157 | 247175 | 247225 | 123087 | 123134 | 2.96E-10 |
| chr1 | chr1 | P | 43 | 95.349 | 350679 | 350721 | 123088 | 123130 | 2.96E-10 |
| chr1 | chr1 | F | 43 | 95.349 | 205942 | 205984 | 123088 | 123130 | 2.96E-10 |
| chr1 | chr1 | P | 43 | 95.349 | 357041 | 357083 | 123228 | 123270 | 2.96E-10 |
| chr1 | chr1 | P | 43 | 95.349 | 176590 | 176632 | 126083 | 126125 | 2.96E-10 |
| chr1 | chr1 | P | 62 | 87.097 | 337637 | 337697 | 140228 | 140288 | 2.96E-10 |
| chr1 | chr1 | P | 40 | 97.5 | 350424 | 350463 | 144644 | 144683 | 2.96E-10 |
| chr1 | chr1 | P | 46 | 93.478 | 337641 | 337686 | 188648 | 188693 | 2.96E-10 |
| chr1 | chr1 | F | 82 | 82.927 | 356922 | 357000 | 205904 | 205982 | 2.96E-10 |
| chr1 | chr1 | F | 41 | 97.561 | 263232 | 263271 | 222092 | 222132 | 2.96E-10 |
| chr1 | chr1 | P | 79 | 83.544 | 245442 | 245517 | 229528 | 229603 | 2.96E-10 |
| chr1 | chr1 | P | 73 | 84.932 | 229534 | 229603 | 245442 | 245511 | 2.96E-10 |
| chr1 | chr1 | P | 46 | 93.478 | 357005 | 357050 | 247151 | 247196 | 2.96E-10 |
| chr1 | chr1 | P | 41 | 97.561 | 263232 | 263271 | 258691 | 258731 | 2.96E-10 |
| chr1 | chr1 | P | 40 | 97.5 | 415790 | 415829 | 258720 | 258759 | 2.96E-10 |
| chr1 | chr1 | F | 41 | 97.561 | 425019 | 425059 | 263232 | 263271 | 2.96E-10 |
| chr1 | chr1 | P | 223 | 73.094 | 326678 | 326896 | 299156 | 299371 | 2.96E-10 |
| chr1 | chr1 | P | 259 | 72.201 | 299156 | 299407 | 326642 | 326896 | 2.96E-10 |
| chr1 | chr1 | P | 37 | 100 | 123234 | 123270 | 357041 | 357077 | 2.96E-10 |
| chr1 | chr1 | F | 43 | 95.349 | 337715 | 337757 | 12999 | 13039 | 1.06E-09 |
| chr1 | chr1 | P | 43 | 95.349 | 350657 | 350698 | 22393 | 22434 | 1.06E-09 |
| chr1 | chr1 | P | 39 | 97.436 | 415724 | 415762 | 22431 | 22469 | 1.06E-09 |
| chr1 | chr1 | P | 39 | 97.436 | 247159 | 247197 | 30510 | 30548 | 1.06E-09 |
| chr1 | chr1 | P | 49 | 91.837 | 368363 | 368410 | 41477 | 41524 | 1.06E-09 |
| chr1 | chr1 | P | 80 | 83.75 | 229484 | 229557 | 65022 | 65097 | 1.06E-09 |
| chr1 | chr1 | F | 45 | 93.333 | 188632 | 188676 | 68904 | 68948 | 1.06E-09 |
| chr1 | chr1 | F | 54 | 88.889 | 205949 | 206002 | 69039 | 69092 | 1.06E-09 |
| chr1 | chr1 | P | 48 | 91.667 | 114119 | 114166 | 69039 | 69086 | 1.06E-09 |
| chr1 | chr1 | P | 36 | 100 | 286899 | 286934 | 88868 | 88903 | 1.06E-09 |
| chr1 | chr1 | P | 42 | 95.238 | 188635 | 188676 | 106794 | 106835 | 1.06E-09 |
| chr1 | chr1 | P | 42 | 95.238 | 344656 | 344697 | 113886 | 113927 | 1.06E-09 |
| chr1 | chr1 | P | 45 | 93.333 | 106794 | 106838 | 188632 | 188676 | 1.06E-09 |
| chr1 | chr1 | F | 36 | 100 | 398063 | 398098 | 196872 | 196907 | 1.06E-09 |
| chr1 | chr1 | P | 70 | 85.714 | 65031 | 65097 | 229484 | 229548 | 1.06E-09 |
| chr1 | chr1 | F | 42 | 95.238 | 344679 | 344720 | 230187 | 230228 | 1.06E-09 |
| chr1 | chr1 | P | 39 | 97.436 | 290556 | 290594 | 249597 | 249635 | 1.06E-09 |
| chr1 | chr1 | P | 89 | 82.022 | 350610 | 350695 | 258855 | 258937 | 1.06E-09 |
| chr1 | chr1 | P | 60 | 88.333 | 356935 | 356988 | 258926 | 258985 | 1.06E-09 |
| chr1 | chr1 | P | 64 | 87.5 | 356930 | 356986 | 276280 | 276343 | 1.06E-09 |
| chr1 | chr1 | P | 39 | 97.436 | 350701 | 350739 | 337641 | 337679 | 1.06E-09 |
| chr1 | chr1 | P | 110 | 80 | 258855 | 258955 | 350592 | 350695 | 1.06E-09 |
| chr1 | chr1 | P | 43 | 95.349 | 368328 | 368369 | 350657 | 350698 | 1.06E-09 |
| chr1 | chr1 | P | 46 | 93.478 | 415718 | 415762 | 350694 | 350738 | 1.06E-09 |
| chr1 | chr1 | P | 45 | 93.333 | 22431 | 22475 | 415718 | 415762 | 1.06E-09 |
| chr1 | chr1 | F | 70 | 84.286 | 358782 | 358850 | 2781 | 2848 | 3.83E-09 |
| chr1 | chr1 | P | 65 | 86.154 | 114097 | 114157 | 22383 | 22445 | 3.83E-09 |
| chr1 | chr1 | P | 51 | 90.196 | 41475 | 41524 | 22428 | 22477 | 3.83E-09 |
| chr1 | chr1 | F | 38 | 97.368 | 140247 | 140284 | 22439 | 22476 | 3.83E-09 |
| chr1 | chr1 | P | 54 | 88.889 | 350691 | 350743 | 26269 | 26321 | 3.83E-09 |
| chr1 | chr1 | P | 38 | 97.368 | 140247 | 140284 | 26273 | 26310 | 3.83E-09 |
| chr1 | chr1 | F | 38 | 97.368 | 243787 | 243824 | 26275 | 26312 | 3.83E-09 |
| chr1 | chr1 | F | 56 | 87.5 | 140229 | 140284 | 30494 | 30549 | 3.83E-09 |
| chr1 | chr1 | F | 48 | 91.667 | 188648 | 188694 | 30504 | 30550 | 3.83E-09 |
| chr1 | chr1 | P | 38 | 97.368 | 243787 | 243824 | 30510 | 30547 | 3.83E-09 |
| chr1 | chr1 | P | 44 | 93.182 | 114131 | 114174 | 41493 | 41536 | 3.83E-09 |
| chr1 | chr1 | F | 41 | 95.122 | 356960 | 357000 | 41494 | 41534 | 3.83E-09 |
| chr1 | chr1 | F | 35 | 100 | 258855 | 258889 | 41609 | 41643 | 3.83E-09 |
| chr1 | chr1 | P | 51 | 90.196 | 123298 | 123347 | 42079 | 42129 | 3.83E-09 |
| chr1 | chr1 | P | 55 | 89.091 | 415685 | 415739 | 43668 | 43718 | 3.83E-09 |
| chr1 | chr1 | F | 98 | 78.571 | 100450 | 100547 | 50701 | 50798 | 3.83E-09 |
| chr1 | chr1 | F | 35 | 100 | 258903 | 258937 | 65029 | 65063 | 3.83E-09 |
| chr1 | chr1 | P | 41 | 95.122 | 176589 | 176629 | 68851 | 68891 | 3.83E-09 |
| chr1 | chr1 | P | 41 | 95.122 | 123088 | 123128 | 68907 | 68947 | 3.83E-09 |
| chr1 | chr1 | F | 35 | 100 | 141219 | 141253 | 69132 | 69166 | 3.83E-09 |
| chr1 | chr1 | F | 41 | 95.122 | 123088 | 123128 | 106795 | 106835 | 3.83E-09 |
| chr1 | chr1 | F | 45 | 93.333 | 188655 | 188698 | 106874 | 106917 | 3.83E-09 |
| chr1 | chr1 | P | 65 | 86.154 | 368318 | 368380 | 114097 | 114157 | 3.83E-09 |
| chr1 | chr1 | P | 35 | 100 | 258855 | 258889 | 114113 | 114147 | 3.83E-09 |
| chr1 | chr1 | F | 57 | 87.719 | 193124 | 193179 | 126322 | 126378 | 3.83E-09 |
| chr1 | chr1 | F | 35 | 100 | 245487 | 245521 | 141219 | 141253 | 3.83E-09 |
| chr1 | chr1 | P | 58 | 87.931 | 68851 | 68905 | 176573 | 176629 | 3.83E-09 |
| chr1 | chr1 | F | 57 | 87.719 | 241752 | 241808 | 193124 | 193179 | 3.83E-09 |
| chr1 | chr1 | F | 44 | 93.182 | 304331 | 304374 | 205941 | 205984 | 3.83E-09 |
| chr1 | chr1 | F | 46 | 93.478 | 243803 | 243848 | 205942 | 205984 | 3.83E-09 |
| chr1 | chr1 | F | 46 | 93.478 | 247176 | 247221 | 205942 | 205984 | 3.83E-09 |
| chr1 | chr1 | F | 65 | 86.154 | 313542 | 313601 | 229513 | 229576 | 3.83E-09 |
| chr1 | chr1 | F | 39 | 97.436 | 337737 | 337775 | 229553 | 229590 | 3.83E-09 |
| chr1 | chr1 | F | 41 | 95.122 | 264030 | 264070 | 263888 | 263928 | 3.83E-09 |
| chr1 | chr1 | P | 35 | 100 | 365561 | 365595 | 304283 | 304317 | 3.83E-09 |
| chr1 | chr1 | P | 51 | 90.196 | 356998 | 357047 | 304310 | 304359 | 3.83E-09 |
| chr1 | chr1 | F | 83 | 81.928 | 350567 | 350649 | 313504 | 313580 | 3.83E-09 |
| chr1 | chr1 | F | 48 | 91.667 | 350659 | 350705 | 337567 | 337613 | 3.83E-09 |
| chr1 | chr1 | F | 60 | 86.667 | 415714 | 415772 | 337638 | 337696 | 3.83E-09 |
| chr1 | chr1 | P | 48 | 91.667 | 304313 | 304359 | 356998 | 357044 | 3.83E-09 |
| chr1 | chr1 | P | 64 | 85.938 | 43668 | 43727 | 415676 | 415739 | 3.83E-09 |
| chr1 | chr1 | F | 50 | 90 | 124275 | 124324 | 659 | 707 | 1.38E-08 |
| chr1 | chr1 | P | 34 | 100 | 362840 | 362873 | 3616 | 3649 | 1.38E-08 |
| chr1 | chr1 | F | 43 | 93.023 | 106874 | 106916 | 22438 | 22480 | 1.38E-08 |
| chr1 | chr1 | F | 77 | 81.818 | 29380 | 29455 | 29215 | 29290 | 1.38E-08 |
| chr1 | chr1 | P | 34 | 100 | 344656 | 344689 | 35074 | 35107 | 1.38E-08 |
| chr1 | chr1 | P | 46 | 91.304 | 356998 | 357043 | 41565 | 41610 | 1.38E-08 |
| chr1 | chr1 | P | 38 | 97.368 | 235876 | 235913 | 45030 | 45066 | 1.38E-08 |
| chr1 | chr1 | P | 47 | 91.489 | 202024 | 202069 | 64967 | 65013 | 1.38E-08 |
| chr1 | chr1 | P | 34 | 100 | 263991 | 264024 | 69004 | 69037 | 1.38E-08 |
| chr1 | chr1 | P | 44 | 93.182 | 304310 | 304352 | 106874 | 106916 | 1.38E-08 |
| chr1 | chr1 | F | 43 | 93.023 | 190983 | 191025 | 116703 | 116745 | 1.38E-08 |
| chr1 | chr1 | P | 40 | 95 | 247212 | 247251 | 123095 | 123134 | 1.38E-08 |
| chr1 | chr1 | P | 55 | 87.273 | 415725 | 415779 | 140222 | 140276 | 1.38E-08 |
| chr1 | chr1 | F | 37 | 97.297 | 359128 | 359164 | 185538 | 185574 | 1.38E-08 |
| chr1 | chr1 | P | 58 | 87.931 | 247165 | 247221 | 188633 | 188686 | 1.38E-08 |
| chr1 | chr1 | P | 58 | 87.931 | 243792 | 243848 | 188633 | 188686 | 1.38E-08 |
| chr1 | chr1 | F | 34 | 100 | 255444 | 255477 | 190152 | 190185 | 1.38E-08 |
| chr1 | chr1 | F | 34 | 100 | 304448 | 304481 | 201926 | 201959 | 1.38E-08 |
| chr1 | chr1 | P | 51 | 90.196 | 258993 | 259042 | 202069 | 202117 | 1.38E-08 |
| chr1 | chr1 | P | 38 | 97.368 | 358831 | 358867 | 206393 | 206430 | 1.38E-08 |
| chr1 | chr1 | P | 34 | 100 | 263323 | 263356 | 229484 | 229517 | 1.38E-08 |
| chr1 | chr1 | P | 38 | 97.368 | 294286 | 294322 | 235876 | 235913 | 1.38E-08 |
| chr1 | chr1 | P | 37 | 97.297 | 357005 | 357041 | 243787 | 243823 | 1.38E-08 |
| chr1 | chr1 | F | 45 | 93.333 | 337589 | 337630 | 245456 | 245500 | 1.38E-08 |
| chr1 | chr1 | P | 64 | 85.938 | 188633 | 188692 | 247159 | 247221 | 1.38E-08 |
| chr1 | chr1 | F | 34 | 100 | 304448 | 304481 | 247503 | 247536 | 1.38E-08 |
| chr1 | chr1 | P | 37 | 97.297 | 365551 | 365587 | 258833 | 258869 | 1.38E-08 |
| chr1 | chr1 | P | 60 | 86.667 | 202072 | 202129 | 258981 | 259039 | 1.38E-08 |
| chr1 | chr1 | P | 40 | 95 | 229484 | 229523 | 263317 | 263356 | 1.38E-08 |
| chr1 | chr1 | P | 34 | 100 | 398065 | 398098 | 337831 | 337864 | 1.38E-08 |
| chr1 | chr1 | P | 34 | 100 | 399226 | 399259 | 398061 | 398094 | 1.38E-08 |
| chr1 | chr1 | P | 37 | 97.297 | 337831 | 337867 | 398062 | 398098 | 1.38E-08 |
| chr1 | chr1 | P | 34 | 100 | 406102 | 406135 | 398065 | 398098 | 1.38E-08 |
| chr1 | chr1 | P | 52 | 88.462 | 140225 | 140276 | 415725 | 415776 | 1.38E-08 |
| chr1 | chr1 | F | 33 | 100 | 249629 | 249661 | 3790 | 3822 | 4.95E-08 |
| chr1 | chr1 | F | 37 | 97.297 | 170192 | 170228 | 8686 | 8721 | 4.95E-08 |
| chr1 | chr1 | P | 39 | 94.872 | 255966 | 256004 | 14368 | 14406 | 4.95E-08 |
| chr1 | chr1 | F | 61 | 86.885 | 356935 | 356988 | 22294 | 22354 | 4.95E-08 |
| chr1 | chr1 | P | 43 | 93.023 | 41607 | 41648 | 26314 | 26355 | 4.95E-08 |
| chr1 | chr1 | P | 51 | 88.235 | 415724 | 415774 | 30492 | 30542 | 4.95E-08 |
| chr1 | chr1 | F | 56 | 87.5 | 44976 | 45030 | 30505 | 30557 | 4.95E-08 |
| chr1 | chr1 | F | 56 | 87.5 | 294232 | 294286 | 30505 | 30557 | 4.95E-08 |
| chr1 | chr1 | F | 39 | 94.872 | 337641 | 337679 | 41476 | 41514 | 4.95E-08 |
| chr1 | chr1 | F | 46 | 91.304 | 415718 | 415762 | 41477 | 41521 | 4.95E-08 |
| chr1 | chr1 | F | 42 | 92.857 | 106794 | 106835 | 41582 | 41623 | 4.95E-08 |
| chr1 | chr1 | P | 42 | 92.857 | 68907 | 68948 | 41582 | 41623 | 4.95E-08 |
| chr1 | chr1 | P | 36 | 97.222 | 247216 | 247251 | 41590 | 41625 | 4.95E-08 |
| chr1 | chr1 | F | 39 | 94.872 | 88698 | 88736 | 43439 | 43477 | 4.95E-08 |
| chr1 | chr1 | P | 43 | 93.023 | 313541 | 313582 | 65022 | 65064 | 4.95E-08 |
| chr1 | chr1 | P | 39 | 94.872 | 263997 | 264035 | 68852 | 68890 | 4.95E-08 |
| chr1 | chr1 | F | 39 | 94.872 | 368222 | 368260 | 68852 | 68890 | 4.95E-08 |
| chr1 | chr1 | P | 45 | 91.111 | 41582 | 41626 | 68904 | 68948 | 4.95E-08 |
| chr1 | chr1 | P | 42 | 92.857 | 356960 | 357001 | 68906 | 68947 | 4.95E-08 |
| chr1 | chr1 | P | 42 | 92.857 | 304331 | 304372 | 68907 | 68948 | 4.95E-08 |
| chr1 | chr1 | P | 44 | 93.182 | 247176 | 247219 | 68907 | 68947 | 4.95E-08 |
| chr1 | chr1 | P | 44 | 93.182 | 243803 | 243846 | 68907 | 68947 | 4.95E-08 |
| chr1 | chr1 | F | 42 | 92.857 | 304331 | 304372 | 106794 | 106835 | 4.95E-08 |
| chr1 | chr1 | F | 42 | 92.857 | 356960 | 357001 | 106795 | 106836 | 4.95E-08 |
| chr1 | chr1 | F | 44 | 93.182 | 243803 | 243846 | 106795 | 106835 | 4.95E-08 |
| chr1 | chr1 | F | 44 | 93.182 | 247176 | 247219 | 106795 | 106835 | 4.95E-08 |
| chr1 | chr1 | F | 39 | 94.872 | 202125 | 202163 | 123071 | 123109 | 4.95E-08 |
| chr1 | chr1 | P | 33 | 100 | 391713 | 391745 | 123236 | 123268 | 4.95E-08 |
| chr1 | chr1 | P | 33 | 100 | 313551 | 313583 | 141219 | 141251 | 4.95E-08 |
| chr1 | chr1 | F | 33 | 100 | 304377 | 304409 | 141219 | 141251 | 4.95E-08 |
| chr1 | chr1 | P | 42 | 92.857 | 385172 | 385213 | 164877 | 164918 | 4.95E-08 |
| chr1 | chr1 | P | 92 | 80.435 | 180253 | 180339 | 180253 | 180339 | 4.95E-08 |
| chr1 | chr1 | F | 46 | 91.304 | 337569 | 337613 | 188615 | 188659 | 4.95E-08 |
| chr1 | chr1 | F | 49 | 89.796 | 368363 | 368410 | 188645 | 188692 | 4.95E-08 |
| chr1 | chr1 | P | 33 | 100 | 337739 | 337771 | 201881 | 201913 | 4.95E-08 |
| chr1 | chr1 | F | 58 | 86.207 | 350623 | 350680 | 202044 | 202100 | 4.95E-08 |
| chr1 | chr1 | P | 40 | 95 | 264091 | 264128 | 206105 | 206144 | 4.95E-08 |
| chr1 | chr1 | F | 45 | 91.111 | 415717 | 415761 | 247158 | 247202 | 4.95E-08 |
| chr1 | chr1 | P | 72 | 83.333 | 247183 | 247251 | 247183 | 247251 | 4.95E-08 |
| chr1 | chr1 | F | 52 | 88.462 | 263991 | 264042 | 247253 | 247302 | 4.95E-08 |
| chr1 | chr1 | P | 33 | 100 | 372350 | 372382 | 249629 | 249661 | 4.95E-08 |
| chr1 | chr1 | F | 33 | 100 | 368555 | 368587 | 249629 | 249661 | 4.95E-08 |
| chr1 | chr1 | F | 42 | 92.857 | 367610 | 367651 | 310373 | 310414 | 4.95E-08 |
| chr1 | chr1 | F | 61 | 86.885 | 368229 | 368289 | 356935 | 356988 | 4.95E-08 |
| chr1 | chr1 | F | 33 | 100 | 391713 | 391745 | 357043 | 357075 | 4.95E-08 |
| chr1 | chr1 | P | 57 | 85.965 | 30492 | 30548 | 415718 | 415774 | 4.95E-08 |
| chr1 | chr1 | P | 38 | 94.737 | 405045 | 405082 | 1928 | 1965 | 1.78E-07 |
| chr1 | chr1 | F | 38 | 94.737 | 68853 | 68890 | 22288 | 22325 | 1.78E-07 |
| chr1 | chr1 | F | 42 | 92.857 | 41607 | 41647 | 22394 | 22434 | 1.78E-07 |
| chr1 | chr1 | P | 54 | 87.037 | 304310 | 304362 | 22428 | 22480 | 1.78E-07 |
| chr1 | chr1 | F | 32 | 100 | 68917 | 68948 | 22428 | 22459 | 1.78E-07 |
| chr1 | chr1 | P | 32 | 100 | 205941 | 205972 | 22428 | 22459 | 1.78E-07 |
| chr1 | chr1 | P | 32 | 100 | 106794 | 106825 | 22428 | 22459 | 1.78E-07 |
| chr1 | chr1 | F | 51 | 88.235 | 304313 | 304362 | 26272 | 26321 | 1.78E-07 |
| chr1 | chr1 | P | 49 | 89.796 | 294232 | 294280 | 26272 | 26317 | 1.78E-07 |
| chr1 | chr1 | P | 49 | 89.796 | 44976 | 45024 | 26272 | 26317 | 1.78E-07 |
| chr1 | chr1 | P | 32 | 100 | 114143 | 114174 | 26290 | 26321 | 1.78E-07 |
| chr1 | chr1 | P | 32 | 100 | 68878 | 68909 | 26352 | 26383 | 1.78E-07 |
| chr1 | chr1 | F | 42 | 92.857 | 368329 | 368369 | 41607 | 41647 | 1.78E-07 |
| chr1 | chr1 | P | 39 | 94.872 | 258881 | 258919 | 43656 | 43693 | 1.78E-07 |
| chr1 | chr1 | F | 42 | 92.857 | 304378 | 304418 | 65022 | 65063 | 1.78E-07 |
| chr1 | chr1 | P | 32 | 100 | 245469 | 245500 | 68878 | 68909 | 1.78E-07 |
| chr1 | chr1 | F | 32 | 100 | 313570 | 313601 | 68878 | 68909 | 1.78E-07 |
| chr1 | chr1 | F | 41 | 92.683 | 202006 | 202046 | 68951 | 68991 | 1.78E-07 |
| chr1 | chr1 | P | 62 | 83.871 | 69056 | 69117 | 69056 | 69117 | 1.78E-07 |
| chr1 | chr1 | P | 41 | 92.683 | 202006 | 202046 | 106751 | 106791 | 1.78E-07 |
| chr1 | chr1 | P | 38 | 94.737 | 415718 | 415755 | 106874 | 106911 | 1.78E-07 |
| chr1 | chr1 | P | 32 | 100 | 247165 | 247196 | 106874 | 106905 | 1.78E-07 |
| chr1 | chr1 | P | 32 | 100 | 243792 | 243823 | 106874 | 106905 | 1.78E-07 |
| chr1 | chr1 | P | 46 | 91.304 | 247176 | 247221 | 114131 | 114173 | 1.78E-07 |
| chr1 | chr1 | P | 46 | 91.304 | 243803 | 243848 | 114131 | 114173 | 1.78E-07 |
| chr1 | chr1 | P | 49 | 89.796 | 264035 | 264083 | 114147 | 114192 | 1.78E-07 |
| chr1 | chr1 | F | 32 | 100 | 206173 | 206204 | 119011 | 119042 | 1.78E-07 |
| chr1 | chr1 | P | 32 | 100 | 385476 | 385507 | 139439 | 139470 | 1.78E-07 |
| chr1 | chr1 | P | 35 | 97.143 | 304377 | 304411 | 141198 | 141232 | 1.78E-07 |
| chr1 | chr1 | F | 35 | 97.143 | 313549 | 313583 | 141198 | 141232 | 1.78E-07 |
| chr1 | chr1 | F | 32 | 100 | 276307 | 276338 | 176590 | 176621 | 1.78E-07 |
| chr1 | chr1 | F | 57 | 85.965 | 356998 | 357053 | 188648 | 188703 | 1.78E-07 |
| chr1 | chr1 | P | 32 | 100 | 399226 | 399257 | 196872 | 196903 | 1.78E-07 |
| chr1 | chr1 | F | 38 | 94.737 | 415724 | 415761 | 243792 | 243829 | 1.78E-07 |
| chr1 | chr1 | P | 38 | 94.737 | 106874 | 106911 | 247159 | 247196 | 1.78E-07 |
| chr1 | chr1 | F | 32 | 100 | 350568 | 350599 | 264030 | 264061 | 1.78E-07 |
| chr1 | chr1 | P | 42 | 92.857 | 114152 | 114192 | 264035 | 264076 | 1.78E-07 |
| chr1 | chr1 | F | 41 | 92.683 | 356960 | 357000 | 304332 | 304372 | 1.78E-07 |
| chr1 | chr1 | F | 44 | 90.909 | 331647 | 331690 | 331557 | 331600 | 1.78E-07 |
| chr1 | chr1 | P | 41 | 92.683 | 356960 | 357000 | 350681 | 350721 | 1.78E-07 |
| chr1 | chr1 | P | 35 | 97.143 | 1931 | 1965 | 405045 | 405079 | 1.78E-07 |
| chr1 | chr1 | P | 32 | 100 | 414203 | 414234 | 408009 | 408040 | 1.78E-07 |
| chr1 | chr1 | P | 37 | 94.595 | 144572 | 144608 | 10013 | 10049 | 6.40E-07 |
| chr1 | chr1 | P | 31 | 100 | 114055 | 114085 | 13000 | 13030 | 6.40E-07 |
| chr1 | chr1 | P | 31 | 100 | 365501 | 365531 | 22289 | 22319 | 6.40E-07 |
| chr1 | chr1 | P | 40 | 92.5 | 106874 | 106913 | 26272 | 26311 | 6.40E-07 |
| chr1 | chr1 | P | 50 | 88 | 258906 | 258955 | 26291 | 26338 | 6.40E-07 |
| chr1 | chr1 | F | 31 | 100 | 123088 | 123118 | 26291 | 26321 | 6.40E-07 |
| chr1 | chr1 | F | 31 | 100 | 141219 | 141249 | 26370 | 26400 | 6.40E-07 |
| chr1 | chr1 | F | 40 | 92.5 | 106874 | 106913 | 30511 | 30550 | 6.40E-07 |
| chr1 | chr1 | P | 38 | 94.737 | 34658 | 34694 | 34658 | 34694 | 6.40E-07 |
| chr1 | chr1 | F | 34 | 97.059 | 230072 | 230105 | 37548 | 37581 | 6.40E-07 |
| chr1 | chr1 | F | 65 | 83.077 | 351542 | 351605 | 40198 | 40262 | 6.40E-07 |
| chr1 | chr1 | P | 40 | 92.5 | 247212 | 247251 | 41501 | 41540 | 6.40E-07 |
| chr1 | chr1 | P | 38 | 94.737 | 229528 | 229564 | 41533 | 41569 | 6.40E-07 |
| chr1 | chr1 | P | 48 | 89.583 | 294232 | 294279 | 41565 | 41609 | 6.40E-07 |
| chr1 | chr1 | P | 48 | 89.583 | 44976 | 45023 | 41565 | 41609 | 6.40E-07 |
| chr1 | chr1 | F | 38 | 94.737 | 313598 | 313634 | 41607 | 41643 | 6.40E-07 |
| chr1 | chr1 | P | 38 | 94.737 | 144857 | 144893 | 68600 | 68637 | 6.40E-07 |
| chr1 | chr1 | P | 38 | 94.737 | 247260 | 247295 | 68852 | 68889 | 6.40E-07 |
| chr1 | chr1 | F | 31 | 100 | 368363 | 368393 | 68917 | 68947 | 6.40E-07 |
| chr1 | chr1 | P | 31 | 100 | 141202 | 141232 | 69132 | 69162 | 6.40E-07 |
| chr1 | chr1 | F | 56 | 85.714 | 98226 | 98280 | 93897 | 93951 | 6.40E-07 |
| chr1 | chr1 | P | 31 | 100 | 368363 | 368393 | 106795 | 106825 | 6.40E-07 |
| chr1 | chr1 | F | 31 | 100 | 276341 | 276371 | 106848 | 106878 | 6.40E-07 |
| chr1 | chr1 | F | 41 | 92.683 | 350701 | 350740 | 106874 | 106913 | 6.40E-07 |
| chr1 | chr1 | F | 40 | 92.5 | 357005 | 357044 | 106874 | 106913 | 6.40E-07 |
| chr1 | chr1 | P | 49 | 89.796 | 313587 | 313634 | 114113 | 114157 | 6.40E-07 |
| chr1 | chr1 | P | 43 | 90.698 | 202142 | 202184 | 140224 | 140266 | 6.40E-07 |
| chr1 | chr1 | P | 37 | 94.595 | 247159 | 247195 | 140247 | 140283 | 6.40E-07 |
| chr1 | chr1 | P | 31 | 100 | 245487 | 245517 | 141202 | 141232 | 6.40E-07 |
| chr1 | chr1 | F | 31 | 100 | 365501 | 365531 | 176596 | 176626 | 6.40E-07 |
| chr1 | chr1 | P | 34 | 97.059 | 337699 | 337732 | 202007 | 202040 | 6.40E-07 |
| chr1 | chr1 | P | 31 | 100 | 368363 | 368393 | 205942 | 205972 | 6.40E-07 |
| chr1 | chr1 | P | 40 | 92.5 | 291532 | 291571 | 230119 | 230158 | 6.40E-07 |
| chr1 | chr1 | F | 43 | 90.698 | 365454 | 365496 | 247260 | 247302 | 6.40E-07 |
| chr1 | chr1 | F | 31 | 100 | 276341 | 276371 | 263955 | 263985 | 6.40E-07 |
| chr1 | chr1 | F | 31 | 100 | 365501 | 365531 | 264003 | 264033 | 6.40E-07 |
| chr1 | chr1 | P | 40 | 92.5 | 321158 | 321197 | 291532 | 291571 | 6.40E-07 |
| chr1 | chr1 | F | 46 | 89.13 | 368373 | 368418 | 357005 | 357050 | 6.40E-07 |
| chr1 | chr1 | P | 31 | 100 | 368224 | 368254 | 365501 | 365531 | 6.40E-07 |
| chr1 | chr1 | F | 42 | 90.476 | 122505 | 122546 | 2841 | 2882 | 2.30E-06 |
| chr1 | chr1 | F | 30 | 100 | 370015 | 370044 | 3940 | 3969 | 2.30E-06 |
| chr1 | chr1 | P | 56 | 85.714 | 19950 | 20003 | 19947 | 20000 | 2.30E-06 |
| chr1 | chr1 | F | 37 | 94.595 | 258855 | 258890 | 22396 | 22431 | 2.30E-06 |
| chr1 | chr1 | F | 39 | 92.308 | 415718 | 415756 | 26274 | 26312 | 2.30E-06 |
| chr1 | chr1 | P | 37 | 94.595 | 258855 | 258890 | 26318 | 26353 | 2.30E-06 |
| chr1 | chr1 | F | 46 | 89.13 | 368361 | 368404 | 30497 | 30542 | 2.30E-06 |
| chr1 | chr1 | P | 40 | 92.5 | 357040 | 357078 | 65059 | 65098 | 2.30E-06 |
| chr1 | chr1 | F | 37 | 94.595 | 356930 | 356964 | 68854 | 68890 | 2.30E-06 |
| chr1 | chr1 | P | 48 | 87.5 | 350667 | 350714 | 69039 | 69086 | 2.30E-06 |
| chr1 | chr1 | P | 30 | 100 | 247192 | 247221 | 69099 | 69128 | 2.30E-06 |
| chr1 | chr1 | P | 30 | 100 | 243819 | 243848 | 69099 | 69128 | 2.30E-06 |
| chr1 | chr1 | F | 36 | 94.444 | 247216 | 247251 | 114131 | 114166 | 2.30E-06 |
| chr1 | chr1 | P | 30 | 100 | 286143 | 286172 | 121945 | 121974 | 2.30E-06 |
| chr1 | chr1 | P | 56 | 85.714 | 229475 | 229526 | 123224 | 123279 | 2.30E-06 |
| chr1 | chr1 | P | 36 | 94.444 | 243787 | 243822 | 140247 | 140282 | 2.30E-06 |
| chr1 | chr1 | F | 36 | 94.444 | 247216 | 247251 | 188633 | 188668 | 2.30E-06 |
| chr1 | chr1 | P | 46 | 89.13 | 415718 | 415762 | 188648 | 188692 | 2.30E-06 |
| chr1 | chr1 | P | 30 | 100 | 249559 | 249588 | 195705 | 195734 | 2.30E-06 |
| chr1 | chr1 | P | 42 | 90.476 | 356984 | 357025 | 202142 | 202183 | 2.30E-06 |
| chr1 | chr1 | F | 39 | 92.308 | 337659 | 337697 | 202142 | 202180 | 2.30E-06 |
| chr1 | chr1 | P | 36 | 94.444 | 205893 | 205928 | 205893 | 205928 | 2.30E-06 |
| chr1 | chr1 | F | 42 | 90.476 | 367610 | 367651 | 221577 | 221618 | 2.30E-06 |
| chr1 | chr1 | P | 34 | 97.059 | 339173 | 339205 | 223836 | 223869 | 2.30E-06 |
| chr1 | chr1 | P | 30 | 100 | 229619 | 229648 | 228126 | 228155 | 2.30E-06 |
| chr1 | chr1 | P | 54 | 87.037 | 337609 | 337662 | 229602 | 229649 | 2.30E-06 |
| chr1 | chr1 | P | 33 | 96.97 | 228126 | 228158 | 229616 | 229648 | 2.30E-06 |
| chr1 | chr1 | P | 43 | 90.698 | 263317 | 263359 | 247113 | 247154 | 2.30E-06 |
| chr1 | chr1 | F | 34 | 97.059 | 357051 | 357083 | 247120 | 247153 | 2.30E-06 |
| chr1 | chr1 | P | 37 | 94.595 | 337568 | 337603 | 258855 | 258890 | 2.30E-06 |
| chr1 | chr1 | F | 37 | 94.595 | 313600 | 313635 | 258855 | 258890 | 2.30E-06 |
| chr1 | chr1 | F | 37 | 94.595 | 368331 | 368366 | 258855 | 258890 | 2.30E-06 |
| chr1 | chr1 | F | 60 | 85 | 263981 | 264035 | 258934 | 258992 | 2.30E-06 |
| chr1 | chr1 | P | 30 | 100 | 339621 | 339650 | 293796 | 293825 | 2.30E-06 |
| chr1 | chr1 | P | 49 | 87.755 | 368363 | 368410 | 304315 | 304362 | 2.30E-06 |
| chr1 | chr1 | P | 40 | 92.5 | 350610 | 350649 | 304380 | 304418 | 2.30E-06 |
| chr1 | chr1 | F | 56 | 85.714 | 313214 | 313268 | 311750 | 311803 | 2.30E-06 |
| chr1 | chr1 | P | 80 | 81.25 | 380426 | 380495 | 337692 | 337771 | 2.30E-06 |
| chr1 | chr1 | F | 30 | 100 | 399226 | 399255 | 337835 | 337864 | 2.30E-06 |
| chr1 | chr1 | P | 34 | 97.059 | 426763 | 426796 | 339173 | 339205 | 2.30E-06 |
| chr1 | chr1 | P | 30 | 100 | 415691 | 415720 | 346923 | 346952 | 2.30E-06 |
| chr1 | chr1 | F | 43 | 90.698 | 357005 | 357046 | 350701 | 350742 | 2.30E-06 |
| chr1 | chr1 | P | 43 | 90.698 | 365453 | 365493 | 356922 | 356964 | 2.30E-06 |
| chr1 | chr1 | F | 42 | 90.476 | 424504 | 424545 | 367610 | 367651 | 2.30E-06 |
| chr1 | chr1 | P | 30 | 100 | 415691 | 415720 | 375566 | 375595 | 2.30E-06 |
| chr1 | chr1 | F | 30 | 100 | 393130 | 393159 | 391072 | 391101 | 2.30E-06 |
| chr1 | chr1 | F | 30 | 100 | 406106 | 406135 | 399226 | 399255 | 2.30E-06 |
| chr1 | chr1 | P | 40 | 92.5 | 188654 | 188692 | 415718 | 415756 | 2.30E-06 |
| chr1 | chr1 | F | 32 | 96.875 | 2080 | 2111 | 2007 | 2038 | 8.28E-06 |
| chr1 | chr1 | P | 33 | 96.97 | 254053 | 254085 | 2929 | 2960 | 8.28E-06 |
| chr1 | chr1 | F | 46 | 89.13 | 202070 | 202112 | 22343 | 22388 | 8.28E-06 |
| chr1 | chr1 | P | 53 | 86.792 | 350591 | 350641 | 22411 | 22459 | 8.28E-06 |
| chr1 | chr1 | F | 46 | 89.13 | 44981 | 45026 | 22437 | 22479 | 8.28E-06 |
| chr1 | chr1 | F | 46 | 89.13 | 294237 | 294282 | 22437 | 22479 | 8.28E-06 |
| chr1 | chr1 | F | 51 | 86.275 | 41475 | 41524 | 26272 | 26321 | 8.28E-06 |
| chr1 | chr1 | P | 42 | 90.476 | 350719 | 350760 | 26299 | 26338 | 8.28E-06 |
| chr1 | chr1 | F | 32 | 96.875 | 313541 | 313572 | 26307 | 26338 | 8.28E-06 |
| chr1 | chr1 | P | 48 | 87.5 | 304313 | 304359 | 30504 | 30550 | 8.28E-06 |
| chr1 | chr1 | F | 48 | 87.5 | 350694 | 350740 | 30504 | 30550 | 8.28E-06 |
| chr1 | chr1 | F | 29 | 100 | 114146 | 114174 | 30504 | 30532 | 8.28E-06 |
| chr1 | chr1 | P | 29 | 100 | 376224 | 376252 | 37413 | 37441 | 8.28E-06 |
| chr1 | chr1 | P | 41 | 90.244 | 368363 | 368403 | 41573 | 41613 | 8.28E-06 |
| chr1 | chr1 | P | 29 | 100 | 176572 | 176600 | 41683 | 41711 | 8.28E-06 |
| chr1 | chr1 | P | 29 | 100 | 245495 | 245523 | 43644 | 43672 | 8.28E-06 |
| chr1 | chr1 | P | 42 | 90.476 | 229584 | 229623 | 64910 | 64951 | 8.28E-06 |
| chr1 | chr1 | F | 39 | 92.308 | 259007 | 259044 | 65029 | 65066 | 8.28E-06 |
| chr1 | chr1 | P | 33 | 96.97 | 201881 | 201913 | 68888 | 68919 | 8.28E-06 |
| chr1 | chr1 | P | 29 | 100 | 415734 | 415762 | 68920 | 68948 | 8.28E-06 |
| chr1 | chr1 | F | 56 | 85.714 | 123080 | 123130 | 69019 | 69074 | 8.28E-06 |
| chr1 | chr1 | P | 41 | 90.244 | 375555 | 375595 | 75909 | 75949 | 8.28E-06 |
| chr1 | chr1 | P | 41 | 90.244 | 346912 | 346952 | 75909 | 75949 | 8.28E-06 |
| chr1 | chr1 | P | 32 | 96.875 | 176572 | 176603 | 86505 | 86536 | 8.28E-06 |
| chr1 | chr1 | F | 68 | 80.882 | 137981 | 138048 | 91038 | 91105 | 8.28E-06 |
| chr1 | chr1 | F | 29 | 100 | 415734 | 415762 | 106794 | 106822 | 8.28E-06 |
| chr1 | chr1 | F | 38 | 92.105 | 195706 | 195743 | 122502 | 122539 | 8.28E-06 |
| chr1 | chr1 | F | 38 | 92.105 | 412755 | 412792 | 134773 | 134810 | 8.28E-06 |
| chr1 | chr1 | F | 45 | 88.889 | 295825 | 295869 | 138888 | 138930 | 8.28E-06 |
| chr1 | chr1 | F | 95 | 78.947 | 145274 | 145361 | 145129 | 145219 | 8.28E-06 |
| chr1 | chr1 | P | 32 | 96.875 | 41683 | 41714 | 176569 | 176600 | 8.28E-06 |
| chr1 | chr1 | P | 35 | 94.286 | 258855 | 258889 | 188615 | 188649 | 8.28E-06 |
| chr1 | chr1 | P | 33 | 96.97 | 229555 | 229586 | 201881 | 201913 | 8.28E-06 |
| chr1 | chr1 | F | 46 | 89.13 | 368278 | 368323 | 202070 | 202112 | 8.28E-06 |
| chr1 | chr1 | P | 29 | 100 | 263981 | 264009 | 202134 | 202162 | 8.28E-06 |
| chr1 | chr1 | P | 29 | 100 | 368366 | 368394 | 202141 | 202169 | 8.28E-06 |
| chr1 | chr1 | F | 32 | 96.875 | 380462 | 380493 | 205880 | 205911 | 8.28E-06 |
| chr1 | chr1 | F | 29 | 100 | 415734 | 415762 | 205941 | 205969 | 8.28E-06 |
| chr1 | chr1 | F | 29 | 100 | 258855 | 258883 | 205968 | 205996 | 8.28E-06 |
| chr1 | chr1 | P | 39 | 92.308 | 337608 | 337645 | 228081 | 228119 | 8.28E-06 |
| chr1 | chr1 | F | 38 | 92.105 | 337586 | 337623 | 229571 | 229608 | 8.28E-06 |
| chr1 | chr1 | P | 35 | 94.286 | 263323 | 263357 | 247363 | 247397 | 8.28E-06 |
| chr1 | chr1 | F | 29 | 100 | 337543 | 337571 | 258831 | 258859 | 8.28E-06 |
| chr1 | chr1 | P | 29 | 100 | 188621 | 188649 | 258855 | 258883 | 8.28E-06 |
| chr1 | chr1 | F | 29 | 100 | 350568 | 350596 | 263888 | 263916 | 8.28E-06 |
| chr1 | chr1 | F | 36 | 94.444 | 267789 | 267823 | 264044 | 264078 | 8.28E-06 |
| chr1 | chr1 | F | 39 | 92.308 | 415718 | 415755 | 304315 | 304352 | 8.28E-06 |
| chr1 | chr1 | P | 48 | 87.5 | 228081 | 228128 | 337599 | 337645 | 8.28E-06 |
| chr1 | chr1 | P | 80 | 81.25 | 22411 | 22480 | 350564 | 350641 | 8.28E-06 |
| chr1 | chr1 | P | 29 | 100 | 26310 | 26338 | 350719 | 350747 | 8.28E-06 |
| chr1 | chr2 | P | 647 | 99.536 | 68149 | 68795 | 119657 | 120303 | 0 |
| chr1 | chr2 | F | 359 | 99.164 | 320823 | 321181 | 114763 | 115121 | 0 |
| chr1 | chr2 | P | 267 | 100 | 249569 | 249835 | 94603 | 94869 | 4.12E-138 |
| chr1 | chr2 | F | 266 | 100 | 113882 | 114147 | 102598 | 102863 | 1.48E-137 |
| chr1 | chr2 | F | 202 | 99.01 | 368222 | 368423 | 12618 | 12819 | 1.21E-98 |
| chr1 | chr2 | P | 193 | 99.482 | 209374 | 209566 | 110184 | 110376 | 2.62E-95 |
| chr1 | chr2 | F | 186 | 98.925 | 22284 | 22469 | 12615 | 12800 | 9.50E-90 |
| chr1 | chr2 | P | 170 | 100 | 258720 | 258889 | 102829 | 102998 | 3.44E-84 |
| chr1 | chr2 | P | 226 | 90.708 | 195939 | 196160 | 121614 | 121828 | 7.50E-76 |
| chr1 | chr2 | P | 103 | 100 | 66177 | 66279 | 45212 | 45314 | 6.05E-47 |
| chr1 | chr2 | P | 154 | 88.961 | 205902 | 206047 | 109664 | 109815 | 3.64E-44 |
| chr1 | chr2 | P | 110 | 94.545 | 26270 | 26379 | 12701 | 12810 | 7.88E-41 |
| chr1 | chr2 | P | 143 | 88.112 | 258903 | 259037 | 12574 | 12710 | 6.14E-37 |
| chr1 | chr2 | P | 123 | 89.431 | 350659 | 350781 | 113517 | 113635 | 2.85E-35 |
| chr1 | chr2 | P | 87 | 97.701 | 122742 | 122828 | 110288 | 110374 | 1.03E-34 |
| chr1 | chr2 | F | 89 | 96.629 | 263951 | 264039 | 1671 | 1759 | 3.69E-34 |
| chr1 | chr2 | F | 78 | 100 | 188615 | 188692 | 19152 | 19229 | 4.78E-33 |
| chr1 | chr2 | F | 77 | 100 | 258720 | 258796 | 1495 | 1571 | 1.72E-32 |
| chr1 | chr2 | P | 82 | 97.561 | 179512 | 179593 | 74029 | 74110 | 6.18E-32 |
| chr1 | chr2 | F | 85 | 95.294 | 118958 | 119040 | 102959 | 103043 | 1.03E-29 |
| chr1 | chr2 | P | 92 | 93.478 | 118958 | 119047 | 113219 | 113309 | 1.03E-29 |
| chr1 | chr2 | P | 72 | 100 | 188621 | 188692 | 113556 | 113627 | 1.03E-29 |
| chr1 | chr2 | P | 110 | 89.091 | 263214 | 263319 | 1500 | 1607 | 3.72E-29 |
| chr1 | chr2 | P | 92 | 92.391 | 28428 | 28519 | 120212 | 120303 | 3.72E-29 |
| chr1 | chr2 | F | 80 | 95 | 350659 | 350738 | 19150 | 19229 | 1.73E-27 |
| chr1 | chr2 | F | 73 | 97.26 | 126088 | 126160 | 19226 | 19298 | 6.22E-27 |
| chr1 | chr2 | F | 106 | 88.679 | 243694 | 243794 | 113394 | 113498 | 6.22E-27 |
| chr1 | chr2 | P | 65 | 100 | 304448 | 304512 | 113394 | 113458 | 8.05E-26 |
| chr1 | chr2 | F | 70 | 97.143 | 350489 | 350558 | 34414 | 34483 | 2.90E-25 |
| chr1 | chr2 | F | 69 | 97.101 | 126086 | 126154 | 12617 | 12685 | 1.04E-24 |
| chr1 | chr2 | F | 63 | 100 | 313574 | 313636 | 12701 | 12763 | 1.04E-24 |
| chr1 | chr2 | F | 63 | 100 | 69004 | 69066 | 19236 | 19298 | 1.04E-24 |
| chr1 | chr2 | P | 73 | 95.89 | 206335 | 206406 | 94860 | 94932 | 1.04E-24 |
| chr1 | chr2 | F | 69 | 97.101 | 337545 | 337613 | 109685 | 109753 | 1.04E-24 |
| chr1 | chr2 | F | 94 | 89.362 | 41553 | 41643 | 113541 | 113633 | 3.75E-24 |
| chr1 | chr2 | P | 62 | 100 | 114113 | 114174 | 113572 | 113633 | 3.75E-24 |
| chr1 | chr2 | F | 67 | 97.015 | 205911 | 205977 | 1616 | 1682 | 1.35E-23 |
| chr1 | chr2 | P | 64 | 98.438 | 276280 | 276343 | 19227 | 19290 | 1.35E-23 |
| chr1 | chr2 | F | 89 | 89.888 | 368216 | 368301 | 19219 | 19307 | 4.85E-23 |
| chr1 | chr2 | F | 83 | 91.566 | 22287 | 22366 | 19225 | 19307 | 4.85E-23 |
| chr1 | chr2 | F | 66 | 96.97 | 45227 | 45292 | 69533 | 69598 | 4.85E-23 |
| chr1 | chr2 | P | 66 | 96.97 | 13918 | 13983 | 69533 | 69598 | 4.85E-23 |
| chr1 | chr2 | P | 59 | 100 | 276280 | 276338 | 12625 | 12683 | 1.74E-22 |
| chr1 | chr2 | P | 59 | 100 | 87799 | 87857 | 94857 | 94915 | 1.74E-22 |
| chr1 | chr2 | F | 62 | 98.387 | 305657 | 305718 | 124101 | 124162 | 1.74E-22 |
| chr1 | chr2 | P | 135 | 82.963 | 174414 | 174538 | 13372 | 13500 | 6.27E-22 |
| chr1 | chr2 | P | 80 | 91.25 | 247224 | 247301 | 19219 | 19298 | 6.27E-22 |
| chr1 | chr2 | F | 65 | 96.923 | 350659 | 350722 | 109707 | 109770 | 6.27E-22 |
| chr1 | chr2 | P | 70 | 94.286 | 205941 | 206009 | 19144 | 19213 | 2.25E-21 |
| chr1 | chr2 | P | 72 | 93.056 | 41573 | 41643 | 19152 | 19222 | 8.11E-21 |
| chr1 | chr2 | F | 62 | 96.774 | 114113 | 114174 | 19152 | 19213 | 8.11E-21 |
| chr1 | chr2 | P | 59 | 98.305 | 201901 | 201959 | 113425 | 113483 | 8.11E-21 |
| chr1 | chr2 | F | 72 | 93.056 | 114113 | 114183 | 109709 | 109778 | 2.92E-20 |
| chr1 | chr2 | P | 60 | 96.667 | 304315 | 304374 | 19170 | 19229 | 1.05E-19 |
| chr1 | chr2 | F | 73 | 91.781 | 238793 | 238864 | 30098 | 30169 | 1.05E-19 |
| chr1 | chr2 | P | 57 | 98.246 | 247480 | 247536 | 113425 | 113481 | 1.05E-19 |
| chr1 | chr2 | F | 60 | 96.667 | 304315 | 304374 | 113556 | 113615 | 1.05E-19 |
| chr1 | chr2 | P | 81 | 90.123 | 337567 | 337644 | 12687 | 12763 | 3.77E-19 |
| chr1 | chr2 | P | 53 | 100 | 424889 | 424941 | 109850 | 109902 | 3.77E-19 |
| chr1 | chr2 | P | 53 | 100 | 221962 | 222014 | 109850 | 109902 | 3.77E-19 |
| chr1 | chr2 | F | 52 | 100 | 106844 | 106895 | 1671 | 1722 | 1.36E-18 |
| chr1 | chr2 | F | 55 | 98.182 | 69004 | 69058 | 12629 | 12683 | 1.36E-18 |
| chr1 | chr2 | P | 52 | 100 | 46246 | 46297 | 106878 | 106929 | 1.36E-18 |
| chr1 | chr2 | F | 67 | 92.537 | 205941 | 206007 | 113572 | 113638 | 1.36E-18 |
| chr1 | chr2 | P | 60 | 95 | 41477 | 41536 | 19170 | 19229 | 4.88E-18 |
| chr1 | chr2 | F | 123 | 82.114 | 300162 | 300273 | 42471 | 42593 | 4.88E-18 |
| chr1 | chr2 | F | 54 | 98.148 | 304281 | 304334 | 109675 | 109728 | 4.88E-18 |
| chr1 | chr2 | F | 54 | 98.148 | 68904 | 68957 | 109726 | 109779 | 4.88E-18 |
| chr1 | chr2 | P | 54 | 98.148 | 106785 | 106838 | 109726 | 109779 | 4.88E-18 |
| chr1 | chr2 | F | 60 | 95 | 41477 | 41536 | 113556 | 113615 | 4.88E-18 |
| chr1 | chr2 | F | 74 | 89.189 | 345183 | 345256 | 32778 | 32851 | 1.76E-17 |
| chr1 | chr2 | P | 81 | 87.654 | 413865 | 413944 | 33751 | 33831 | 1.76E-17 |
| chr1 | chr2 | P | 50 | 100 | 101347 | 101396 | 42467 | 42516 | 1.76E-17 |
| chr1 | chr2 | F | 73 | 90.411 | 263248 | 263319 | 102923 | 102993 | 1.76E-17 |
| chr1 | chr2 | F | 50 | 100 | 258720 | 258769 | 113270 | 113319 | 1.76E-17 |
| chr1 | chr2 | P | 120 | 82.5 | 68852 | 68957 | 1637 | 1755 | 6.31E-17 |
| chr1 | chr2 | P | 92 | 85.87 | 247150 | 247234 | 12725 | 12815 | 6.31E-17 |
| chr1 | chr2 | F | 58 | 94.828 | 415790 | 415847 | 102959 | 103016 | 6.31E-17 |
| chr1 | chr2 | P | 58 | 94.828 | 415790 | 415847 | 113252 | 113309 | 6.31E-17 |
| chr1 | chr2 | F | 136 | 79.412 | 89816 | 89949 | 30253 | 30382 | 2.27E-16 |
| chr1 | chr2 | P | 99 | 83.838 | 350700 | 350798 | 109750 | 109843 | 2.27E-16 |
| chr1 | chr2 | F | 47 | 100 | 201847 | 201893 | 102726 | 102772 | 8.17E-16 |
| chr1 | chr2 | P | 63 | 92.063 | 41582 | 41643 | 109709 | 109770 | 8.17E-16 |
| chr1 | chr2 | F | 47 | 100 | 223207 | 223253 | 109851 | 109897 | 8.17E-16 |
| chr1 | chr2 | F | 47 | 100 | 426134 | 426180 | 109851 | 109897 | 8.17E-16 |
| chr1 | chr2 | F | 52 | 96.154 | 64909 | 64960 | 12579 | 12630 | 2.94E-15 |
| chr1 | chr2 | P | 68 | 89.706 | 247230 | 247295 | 12618 | 12685 | 2.94E-15 |
| chr1 | chr2 | P | 211 | 74.408 | 228566 | 228774 | 80855 | 81061 | 2.94E-15 |
| chr1 | chr2 | F | 89 | 85.393 | 206349 | 206430 | 122065 | 122153 | 2.94E-15 |
| chr1 | chr2 | P | 45 | 100 | 126087 | 126131 | 1711 | 1755 | 1.06E-14 |
| chr1 | chr2 | P | 45 | 100 | 263991 | 264035 | 12618 | 12662 | 1.06E-14 |
| chr1 | chr2 | P | 64 | 90.625 | 247151 | 247213 | 12804 | 12867 | 1.06E-14 |
| chr1 | chr2 | F | 80 | 87.5 | 356922 | 356994 | 19221 | 19298 | 1.06E-14 |
| chr1 | chr2 | F | 51 | 96.078 | 13892 | 13942 | 108623 | 108673 | 1.06E-14 |
| chr1 | chr2 | P | 51 | 96.078 | 45268 | 45318 | 108623 | 108673 | 1.06E-14 |
| chr1 | chr2 | P | 48 | 97.917 | 304220 | 304267 | 109850 | 109897 | 1.06E-14 |
| chr1 | chr2 | P | 77 | 87.013 | 122834 | 122907 | 110237 | 110313 | 1.06E-14 |
| chr1 | chr2 | P | 48 | 97.917 | 258745 | 258792 | 113314 | 113361 | 1.06E-14 |
| chr1 | chr2 | F | 63 | 90.476 | 188615 | 188676 | 109709 | 109770 | 3.80E-14 |
| chr1 | chr2 | P | 44 | 100 | 188649 | 188692 | 109805 | 109848 | 3.80E-14 |
| chr1 | chr2 | P | 92 | 83.696 | 337564 | 337652 | 113549 | 113638 | 3.80E-14 |
| chr1 | chr2 | F | 50 | 96 | 230093 | 230142 | 115072 | 115121 | 3.80E-14 |
| chr1 | chr2 | F | 57 | 92.982 | 267771 | 267827 | 1620 | 1673 | 1.37E-13 |
| chr1 | chr2 | F | 46 | 97.826 | 106785 | 106830 | 1637 | 1682 | 1.37E-13 |
| chr1 | chr2 | P | 53 | 94.34 | 356930 | 356980 | 1701 | 1753 | 1.37E-13 |
| chr1 | chr2 | F | 43 | 100 | 276301 | 276343 | 1711 | 1753 | 1.37E-13 |
| chr1 | chr2 | P | 49 | 95.918 | 243792 | 243840 | 12804 | 12852 | 1.37E-13 |
| chr1 | chr2 | F | 102 | 82.353 | 42910 | 43007 | 15222 | 15318 | 1.37E-13 |
| chr1 | chr2 | P | 43 | 100 | 123088 | 123130 | 19170 | 19212 | 1.37E-13 |
| chr1 | chr2 | F | 136 | 77.941 | 89594 | 89726 | 29433 | 29566 | 1.37E-13 |
| chr1 | chr2 | P | 52 | 94.231 | 116585 | 116636 | 62506 | 62557 | 1.37E-13 |
| chr1 | chr2 | P | 99 | 81.818 | 229240 | 229337 | 80301 | 80397 | 1.37E-13 |
| chr1 | chr2 | F | 50 | 96 | 35075 | 35123 | 102611 | 102660 | 1.37E-13 |
| chr1 | chr2 | F | 43 | 100 | 123088 | 123130 | 113573 | 113615 | 1.37E-13 |
| chr1 | chr2 | P | 54 | 92.593 | 415790 | 415842 | 1481 | 1534 | 1.77E-12 |
| chr1 | chr2 | P | 47 | 95.745 | 69095 | 69141 | 12701 | 12747 | 1.77E-12 |
| chr1 | chr2 | P | 41 | 100 | 245456 | 245496 | 12701 | 12741 | 1.77E-12 |
| chr1 | chr2 | F | 48 | 95.833 | 368372 | 368418 | 12820 | 12867 | 1.77E-12 |
| chr1 | chr2 | P | 53 | 92.453 | 69039 | 69091 | 19153 | 19205 | 1.77E-12 |
| chr1 | chr2 | P | 44 | 97.727 | 41493 | 41536 | 109727 | 109770 | 1.77E-12 |
| chr1 | chr2 | P | 45 | 97.778 | 358824 | 358867 | 122116 | 122160 | 1.77E-12 |
| chr1 | chr2 | P | 54 | 92.593 | 118958 | 119008 | 1481 | 1534 | 6.36E-12 |
| chr1 | chr2 | P | 49 | 93.878 | 368218 | 368266 | 1711 | 1759 | 6.36E-12 |
| chr1 | chr2 | P | 40 | 100 | 176590 | 176629 | 12617 | 12656 | 6.36E-12 |
| chr1 | chr2 | F | 53 | 92.453 | 350691 | 350742 | 12759 | 12810 | 6.36E-12 |
| chr1 | chr2 | P | 46 | 95.652 | 415717 | 415762 | 12762 | 12807 | 6.36E-12 |
| chr1 | chr2 | F | 57 | 91.228 | 188639 | 188694 | 12806 | 12860 | 6.36E-12 |
| chr1 | chr2 | F | 40 | 100 | 106874 | 106913 | 12821 | 12860 | 6.36E-12 |
| chr1 | chr2 | P | 93 | 82.796 | 263991 | 264083 | 19186 | 19269 | 6.36E-12 |
| chr1 | chr2 | P | 40 | 100 | 41609 | 41648 | 102824 | 102863 | 6.36E-12 |
| chr1 | chr2 | P | 43 | 97.674 | 313597 | 313639 | 109704 | 109746 | 6.36E-12 |
| chr1 | chr2 | F | 40 | 100 | 26317 | 26356 | 109707 | 109746 | 6.36E-12 |
| chr1 | chr2 | P | 40 | 100 | 368328 | 368367 | 109707 | 109746 | 6.36E-12 |
| chr1 | chr2 | P | 40 | 100 | 22393 | 22432 | 109707 | 109746 | 6.36E-12 |
| chr1 | chr2 | P | 45 | 95.556 | 424992 | 425036 | 1489 | 1533 | 2.29E-11 |
| chr1 | chr2 | P | 45 | 95.556 | 222065 | 222109 | 1489 | 1533 | 2.29E-11 |
| chr1 | chr2 | P | 52 | 92.308 | 26270 | 26321 | 12812 | 12862 | 2.29E-11 |
| chr1 | chr2 | F | 42 | 97.619 | 302990 | 303031 | 42225 | 42266 | 2.29E-11 |
| chr1 | chr2 | P | 63 | 88.889 | 337690 | 337748 | 102771 | 102831 | 2.29E-11 |
| chr1 | chr2 | F | 45 | 95.556 | 222065 | 222109 | 102960 | 103004 | 2.29E-11 |
| chr1 | chr2 | F | 45 | 95.556 | 424992 | 425036 | 102960 | 103004 | 2.29E-11 |
| chr1 | chr2 | P | 45 | 95.556 | 424992 | 425036 | 113264 | 113308 | 2.29E-11 |
| chr1 | chr2 | P | 45 | 95.556 | 222065 | 222109 | 113264 | 113308 | 2.29E-11 |
| chr1 | chr2 | P | 38 | 100 | 22294 | 22331 | 1711 | 1748 | 8.22E-11 |
| chr1 | chr2 | P | 42 | 97.619 | 243792 | 243832 | 12759 | 12800 | 8.22E-11 |
| chr1 | chr2 | F | 38 | 100 | 106874 | 106911 | 12769 | 12806 | 8.22E-11 |
| chr1 | chr2 | F | 95 | 81.053 | 337562 | 337652 | 19144 | 19236 | 8.22E-11 |
| chr1 | chr2 | P | 41 | 97.561 | 356960 | 357000 | 19172 | 19212 | 8.22E-11 |
| chr1 | chr2 | P | 53 | 90.566 | 337634 | 337686 | 19185 | 19237 | 8.22E-11 |
| chr1 | chr2 | F | 38 | 100 | 68853 | 68890 | 19226 | 19263 | 8.22E-11 |
| chr1 | chr2 | P | 42 | 97.619 | 93691 | 93731 | 61015 | 61056 | 8.22E-11 |
| chr1 | chr2 | F | 47 | 93.617 | 344658 | 344704 | 109130 | 109176 | 8.22E-11 |
| chr1 | chr2 | F | 38 | 100 | 304315 | 304352 | 109805 | 109842 | 8.22E-11 |
| chr1 | chr2 | P | 69 | 86.957 | 126191 | 126258 | 113496 | 113558 | 8.22E-11 |
| chr1 | chr2 | P | 65 | 87.692 | 64973 | 65034 | 113515 | 113577 | 8.22E-11 |
| chr1 | chr2 | F | 53 | 90.566 | 337634 | 337686 | 113548 | 113600 | 8.22E-11 |
| chr1 | chr2 | P | 86 | 82.558 | 26317 | 26401 | 113555 | 113635 | 8.22E-11 |
| chr1 | chr2 | F | 41 | 97.561 | 356960 | 357000 | 113573 | 113613 | 8.22E-11 |
| chr1 | chr2 | F | 37 | 100 | 41493 | 41529 | 1646 | 1682 | 2.96E-10 |
| chr1 | chr2 | F | 46 | 93.478 | 30505 | 30550 | 12815 | 12860 | 2.96E-10 |
| chr1 | chr2 | P | 85 | 82.353 | 365462 | 365544 | 19172 | 19252 | 2.96E-10 |
| chr1 | chr2 | F | 290 | 71.724 | 344725 | 345008 | 32251 | 32533 | 2.96E-10 |
| chr1 | chr2 | F | 40 | 97.5 | 350656 | 350695 | 102824 | 102863 | 2.96E-10 |
| chr1 | chr2 | P | 43 | 95.349 | 123088 | 123130 | 109727 | 109769 | 2.96E-10 |
| chr1 | chr2 | P | 64 | 87.5 | 64973 | 65033 | 109765 | 109826 | 2.96E-10 |
| chr1 | chr2 | F | 46 | 93.478 | 337634 | 337679 | 109797 | 109842 | 2.96E-10 |
| chr1 | chr2 | F | 85 | 82.353 | 313553 | 313636 | 113556 | 113635 | 2.96E-10 |
| chr1 | chr2 | P | 43 | 95.349 | 350657 | 350698 | 12724 | 12765 | 1.06E-09 |
| chr1 | chr2 | P | 49 | 91.837 | 41477 | 41524 | 12759 | 12806 | 1.06E-09 |
| chr1 | chr2 | F | 60 | 88.333 | 350687 | 350742 | 12804 | 12862 | 1.06E-09 |
| chr1 | chr2 | F | 45 | 93.333 | 68904 | 68948 | 19169 | 19213 | 1.06E-09 |
| chr1 | chr2 | P | 45 | 93.333 | 106794 | 106838 | 19169 | 19213 | 1.06E-09 |
| chr1 | chr2 | P | 43 | 95.349 | 26280 | 26321 | 19182 | 19223 | 1.06E-09 |
| chr1 | chr2 | P | 64 | 85.938 | 64506 | 64568 | 37171 | 37233 | 1.06E-09 |
| chr1 | chr2 | F | 39 | 97.436 | 290556 | 290594 | 94803 | 94841 | 1.06E-09 |
| chr1 | chr2 | P | 42 | 95.238 | 344656 | 344697 | 102602 | 102643 | 1.06E-09 |
| chr1 | chr2 | F | 39 | 97.436 | 41477 | 41515 | 109805 | 109843 | 1.06E-09 |
| chr1 | chr2 | P | 80 | 83.75 | 205902 | 205972 | 113487 | 113566 | 1.06E-09 |
| chr1 | chr2 | F | 49 | 91.837 | 26274 | 26321 | 113556 | 113603 | 1.06E-09 |
| chr1 | chr2 | F | 42 | 95.238 | 106794 | 106835 | 113572 | 113613 | 1.06E-09 |
| chr1 | chr2 | P | 42 | 95.238 | 68907 | 68948 | 113572 | 113613 | 1.06E-09 |
| chr1 | chr2 | F | 48 | 91.667 | 69039 | 69086 | 113580 | 113627 | 1.06E-09 |
| chr1 | chr2 | F | 36 | 100 | 258855 | 258890 | 113599 | 113634 | 1.06E-09 |
| chr1 | chr2 | F | 53 | 90.566 | 87685 | 87737 | 122004 | 122053 | 1.06E-09 |
| chr1 | chr2 | F | 35 | 100 | 365371 | 365405 | 1608 | 1642 | 3.83E-09 |
| chr1 | chr2 | P | 69 | 85.507 | 101221 | 101284 | 7813 | 7878 | 3.83E-09 |
| chr1 | chr2 | P | 65 | 86.154 | 114097 | 114157 | 12714 | 12776 | 3.83E-09 |
| chr1 | chr2 | P | 65 | 86.154 | 247158 | 247221 | 19170 | 19230 | 3.83E-09 |
| chr1 | chr2 | F | 35 | 100 | 67533 | 67567 | 72501 | 72535 | 3.83E-09 |
| chr1 | chr2 | F | 52 | 90.385 | 26304 | 26353 | 102813 | 102863 | 3.83E-09 |
| chr1 | chr2 | P | 44 | 93.182 | 304331 | 304374 | 109727 | 109770 | 3.83E-09 |
| chr1 | chr2 | P | 46 | 93.478 | 247176 | 247221 | 109727 | 109769 | 3.83E-09 |
| chr1 | chr2 | P | 46 | 93.478 | 243803 | 243848 | 109727 | 109769 | 3.83E-09 |
| chr1 | chr2 | P | 35 | 100 | 199478 | 199512 | 111135 | 111169 | 3.83E-09 |
| chr1 | chr2 | F | 51 | 90.196 | 263251 | 263301 | 113314 | 113362 | 3.83E-09 |
| chr1 | chr2 | F | 65 | 86.154 | 247158 | 247221 | 113555 | 113615 | 3.83E-09 |
| chr1 | chr2 | P | 37 | 97.297 | 350686 | 350722 | 1646 | 1682 | 1.38E-08 |
| chr1 | chr2 | P | 34 | 100 | 69004 | 69037 | 1711 | 1744 | 1.38E-08 |
| chr1 | chr2 | F | 40 | 95 | 68851 | 68890 | 12617 | 12656 | 1.38E-08 |
| chr1 | chr2 | P | 58 | 87.931 | 304313 | 304366 | 12804 | 12860 | 1.38E-08 |
| chr1 | chr2 | P | 47 | 91.489 | 41573 | 41619 | 12806 | 12851 | 1.38E-08 |
| chr1 | chr2 | P | 58 | 87.931 | 243792 | 243848 | 19170 | 19223 | 1.38E-08 |
| chr1 | chr2 | F | 50 | 90 | 368363 | 368411 | 19182 | 19230 | 1.38E-08 |
| chr1 | chr2 | P | 34 | 100 | 156190 | 156223 | 23502 | 23535 | 1.38E-08 |
| chr1 | chr2 | F | 282 | 71.277 | 47107 | 47382 | 68202 | 68480 | 1.38E-08 |
| chr1 | chr2 | P | 64 | 84.375 | 297470 | 297533 | 68841 | 68904 | 1.38E-08 |
| chr1 | chr2 | F | 37 | 97.297 | 365551 | 365587 | 102849 | 102885 | 1.38E-08 |
| chr1 | chr2 | P | 74 | 82.432 | 365524 | 365595 | 109677 | 109750 | 1.38E-08 |
| chr1 | chr2 | F | 71 | 84.507 | 243779 | 243848 | 113551 | 113615 | 1.38E-08 |
| chr1 | chr2 | P | 50 | 90 | 368363 | 368411 | 113555 | 113603 | 1.38E-08 |
| chr1 | chr2 | P | 46 | 91.304 | 114138 | 114183 | 1638 | 1682 | 4.95E-08 |
| chr1 | chr2 | F | 49 | 89.796 | 247253 | 247299 | 1711 | 1759 | 4.95E-08 |
| chr1 | chr2 | F | 39 | 94.872 | 176590 | 176628 | 1717 | 1755 | 4.95E-08 |
| chr1 | chr2 | P | 44 | 93.182 | 313508 | 313551 | 9327 | 9367 | 4.95E-08 |
| chr1 | chr2 | P | 33 | 100 | 263885 | 263917 | 9341 | 9373 | 4.95E-08 |
| chr1 | chr2 | F | 61 | 86.885 | 356935 | 356988 | 12625 | 12685 | 4.95E-08 |
| chr1 | chr2 | F | 49 | 89.796 | 188645 | 188692 | 12759 | 12806 | 4.95E-08 |
| chr1 | chr2 | F | 48 | 89.583 | 356999 | 357046 | 12815 | 12862 | 4.95E-08 |
| chr1 | chr2 | P | 39 | 94.872 | 415718 | 415756 | 12820 | 12858 | 4.95E-08 |
| chr1 | chr2 | F | 40 | 95 | 30504 | 30542 | 19185 | 19223 | 4.95E-08 |
| chr1 | chr2 | P | 36 | 97.222 | 156190 | 156225 | 23358 | 23393 | 4.95E-08 |
| chr1 | chr2 | P | 36 | 97.222 | 156190 | 156225 | 75445 | 75480 | 4.95E-08 |
| chr1 | chr2 | P | 76 | 81.579 | 229873 | 229947 | 80045 | 80119 | 4.95E-08 |
| chr1 | chr2 | P | 33 | 100 | 368555 | 368587 | 94777 | 94809 | 4.95E-08 |
| chr1 | chr2 | F | 33 | 100 | 372350 | 372382 | 94777 | 94809 | 4.95E-08 |
| chr1 | chr2 | P | 33 | 100 | 3790 | 3822 | 94777 | 94809 | 4.95E-08 |
| chr1 | chr2 | P | 55 | 87.273 | 30504 | 30557 | 113547 | 113600 | 4.95E-08 |
| chr1 | chr2 | F | 51 | 90.196 | 22383 | 22432 | 113589 | 113635 | 4.95E-08 |
| chr1 | chr2 | F | 51 | 90.196 | 368318 | 368367 | 113589 | 113635 | 4.95E-08 |
| chr1 | chr2 | P | 43 | 93.023 | 425019 | 425059 | 1548 | 1589 | 1.78E-07 |
| chr1 | chr2 | P | 43 | 93.023 | 222092 | 222132 | 1548 | 1589 | 1.78E-07 |
| chr1 | chr2 | F | 43 | 93.023 | 258691 | 258731 | 1548 | 1589 | 1.78E-07 |
| chr1 | chr2 | P | 49 | 89.796 | 229524 | 229572 | 9327 | 9372 | 1.78E-07 |
| chr1 | chr2 | F | 45 | 91.111 | 202074 | 202117 | 12574 | 12617 | 1.78E-07 |
| chr1 | chr2 | F | 42 | 92.857 | 41607 | 41647 | 12725 | 12765 | 1.78E-07 |
| chr1 | chr2 | F | 41 | 92.683 | 22437 | 22477 | 12820 | 12860 | 1.78E-07 |
| chr1 | chr2 | P | 38 | 94.737 | 176590 | 176627 | 19226 | 19263 | 1.78E-07 |
| chr1 | chr2 | F | 109 | 77.064 | 238736 | 238842 | 31865 | 31971 | 1.78E-07 |
| chr1 | chr2 | P | 52 | 88.462 | 368331 | 368380 | 102813 | 102863 | 1.78E-07 |
| chr1 | chr2 | P | 52 | 88.462 | 22396 | 22445 | 102813 | 102863 | 1.78E-07 |
| chr1 | chr2 | P | 51 | 88.235 | 426022 | 426071 | 102994 | 103043 | 1.78E-07 |
| chr1 | chr2 | P | 51 | 88.235 | 223095 | 223144 | 102994 | 103043 | 1.78E-07 |
| chr1 | chr2 | P | 41 | 92.683 | 356960 | 357000 | 109729 | 109769 | 1.78E-07 |
| chr1 | chr2 | F | 32 | 100 | 22428 | 22459 | 109739 | 109770 | 1.78E-07 |
| chr1 | chr2 | P | 49 | 89.796 | 267779 | 267827 | 109743 | 109788 | 1.78E-07 |
| chr1 | chr2 | P | 54 | 87.037 | 30505 | 30557 | 109796 | 109848 | 1.78E-07 |
| chr1 | chr2 | F | 45 | 91.111 | 26274 | 26317 | 109805 | 109848 | 1.78E-07 |
| chr1 | chr2 | F | 32 | 100 | 123248 | 123279 | 110021 | 110052 | 1.78E-07 |
| chr1 | chr2 | F | 48 | 89.583 | 201811 | 201858 | 124401 | 124447 | 1.78E-07 |
| chr1 | chr2 | F | 37 | 94.595 | 41582 | 41618 | 1646 | 1682 | 6.40E-07 |
| chr1 | chr2 | F | 31 | 100 | 276341 | 276371 | 1675 | 1705 | 6.40E-07 |
| chr1 | chr2 | F | 38 | 94.737 | 41533 | 41570 | 9335 | 9370 | 6.40E-07 |
| chr1 | chr2 | P | 31 | 100 | 365501 | 365531 | 12620 | 12650 | 6.40E-07 |
| chr1 | chr2 | F | 31 | 100 | 68917 | 68947 | 12759 | 12789 | 6.40E-07 |
| chr1 | chr2 | P | 31 | 100 | 205942 | 205972 | 12759 | 12789 | 6.40E-07 |
| chr1 | chr2 | P | 31 | 100 | 106795 | 106825 | 12759 | 12789 | 6.40E-07 |
| chr1 | chr2 | F | 46 | 89.13 | 357005 | 357050 | 12769 | 12814 | 6.40E-07 |
| chr1 | chr2 | P | 58 | 86.207 | 41475 | 41528 | 12804 | 12860 | 6.40E-07 |
| chr1 | chr2 | P | 38 | 94.737 | 123087 | 123124 | 12806 | 12842 | 6.40E-07 |
| chr1 | chr2 | P | 51 | 88.235 | 415714 | 415762 | 19185 | 19234 | 6.40E-07 |
| chr1 | chr2 | F | 31 | 100 | 312760 | 312790 | 29896 | 29926 | 6.40E-07 |
| chr1 | chr2 | F | 86 | 79.07 | 137963 | 138048 | 31118 | 31201 | 6.40E-07 |
| chr1 | chr2 | P | 31 | 100 | 93938 | 93968 | 60590 | 60620 | 6.40E-07 |
| chr1 | chr2 | P | 91 | 79.121 | 237169 | 237256 | 61712 | 61799 | 6.40E-07 |
| chr1 | chr2 | P | 31 | 100 | 13000 | 13030 | 102771 | 102801 | 6.40E-07 |
| chr1 | chr2 | P | 46 | 89.13 | 113886 | 113931 | 109124 | 109169 | 6.40E-07 |
| chr1 | chr2 | P | 38 | 94.737 | 35074 | 35110 | 109124 | 109161 | 6.40E-07 |
| chr1 | chr2 | F | 31 | 100 | 368363 | 368393 | 109739 | 109769 | 6.40E-07 |
| chr1 | chr2 | F | 45 | 91.111 | 415714 | 415756 | 109800 | 109843 | 6.40E-07 |
| chr1 | chr2 | P | 41 | 92.683 | 368372 | 368411 | 109804 | 109843 | 6.40E-07 |
| chr1 | chr2 | F | 50 | 88 | 223096 | 223144 | 113226 | 113274 | 6.40E-07 |
| chr1 | chr2 | F | 50 | 88 | 426023 | 426071 | 113226 | 113274 | 6.40E-07 |
| chr1 | chr2 | P | 49 | 87.755 | 267779 | 267827 | 113491 | 113539 | 6.40E-07 |
| chr1 | chr2 | F | 45 | 91.111 | 415714 | 415756 | 113551 | 113594 | 6.40E-07 |
| chr1 | chr2 | F | 38 | 94.737 | 144857 | 144893 | 119815 | 119852 | 6.40E-07 |
| chr1 | chr2 | F | 41 | 92.683 | 381935 | 381973 | 121688 | 121728 | 6.40E-07 |
| chr1 | chr2 | P | 30 | 100 | 71923 | 71952 | 965 | 994 | 2.30E-06 |
| chr1 | chr2 | P | 30 | 100 | 263424 | 263453 | 1578 | 1607 | 2.30E-06 |
| chr1 | chr2 | F | 68 | 82.353 | 356931 | 356995 | 1616 | 1682 | 2.30E-06 |
| chr1 | chr2 | F | 46 | 89.13 | 117372 | 117416 | 7809 | 7853 | 2.30E-06 |
| chr1 | chr2 | P | 37 | 94.595 | 101221 | 101257 | 7811 | 7845 | 2.30E-06 |
| chr1 | chr2 | F | 37 | 94.595 | 258855 | 258890 | 12727 | 12762 | 2.30E-06 |
| chr1 | chr2 | F | 46 | 89.13 | 30497 | 30542 | 12757 | 12800 | 2.30E-06 |
| chr1 | chr2 | P | 49 | 87.755 | 304315 | 304362 | 12759 | 12806 | 2.30E-06 |
| chr1 | chr2 | F | 37 | 94.595 | 114137 | 114173 | 12806 | 12841 | 2.30E-06 |
| chr1 | chr2 | P | 36 | 94.444 | 258855 | 258890 | 19151 | 19186 | 2.30E-06 |
| chr1 | chr2 | F | 36 | 94.444 | 247216 | 247251 | 19170 | 19205 | 2.30E-06 |
| chr1 | chr2 | F | 43 | 90.698 | 22428 | 22469 | 19182 | 19223 | 2.30E-06 |
| chr1 | chr2 | F | 30 | 100 | 380507 | 380536 | 28361 | 28390 | 2.30E-06 |
| chr1 | chr2 | F | 42 | 90.476 | 137128 | 137169 | 29492 | 29533 | 2.30E-06 |
| chr1 | chr2 | F | 51 | 86.275 | 89668 | 89718 | 30168 | 30218 | 2.30E-06 |
| chr1 | chr2 | F | 116 | 76.724 | 90991 | 91106 | 31096 | 31202 | 2.30E-06 |
| chr1 | chr2 | P | 65 | 83.077 | 305657 | 305718 | 95653 | 95717 | 2.30E-06 |
| chr1 | chr2 | F | 30 | 100 | 335595 | 335624 | 97907 | 97936 | 2.30E-06 |
| chr1 | chr2 | F | 50 | 88 | 266070 | 266119 | 109647 | 109692 | 2.30E-06 |
| chr1 | chr2 | P | 37 | 94.595 | 258855 | 258890 | 109708 | 109743 | 2.30E-06 |
| chr1 | chr2 | P | 49 | 87.755 | 22428 | 22475 | 113556 | 113603 | 2.30E-06 |
| chr1 | chr2 | P | 36 | 94.444 | 247216 | 247251 | 113580 | 113615 | 2.30E-06 |
| chr1 | chr2 | F | 36 | 94.444 | 310286 | 310321 | 124411 | 124446 | 2.30E-06 |
| chr1 | chr2 | P | 32 | 96.875 | 22428 | 22459 | 1646 | 1677 | 8.28E-06 |
| chr1 | chr2 | P | 29 | 100 | 202134 | 202162 | 1701 | 1729 | 8.28E-06 |
| chr1 | chr2 | F | 32 | 96.875 | 365462 | 365493 | 1728 | 1759 | 8.28E-06 |
| chr1 | chr2 | P | 29 | 100 | 380507 | 380535 | 2585 | 2613 | 8.28E-06 |
| chr1 | chr2 | P | 29 | 100 | 350568 | 350596 | 9342 | 9370 | 8.28E-06 |
| chr1 | chr2 | P | 29 | 100 | 264030 | 264058 | 9342 | 9370 | 8.28E-06 |
| chr1 | chr2 | F | 46 | 89.13 | 202070 | 202112 | 12674 | 12719 | 8.28E-06 |
| chr1 | chr2 | P | 41 | 90.244 | 41573 | 41613 | 12759 | 12799 | 8.28E-06 |
| chr1 | chr2 | P | 29 | 100 | 202141 | 202169 | 12762 | 12790 | 8.28E-06 |
| chr1 | chr2 | F | 39 | 92.308 | 106874 | 106911 | 19192 | 19229 | 8.28E-06 |
| chr1 | chr2 | F | 130 | 75.385 | 137226 | 137354 | 30262 | 30383 | 8.28E-06 |
| chr1 | chr2 | P | 36 | 94.444 | 98275 | 98310 | 59636 | 59670 | 8.28E-06 |
| chr1 | chr2 | P | 32 | 96.875 | 276479 | 276510 | 83917 | 83948 | 8.28E-06 |
| chr1 | chr2 | P | 36 | 94.444 | 313600 | 313634 | 102829 | 102863 | 8.28E-06 |
| chr1 | chr2 | F | 35 | 94.286 | 188615 | 188649 | 102829 | 102863 | 8.28E-06 |
| chr1 | chr2 | F | 36 | 94.444 | 337569 | 337603 | 102829 | 102863 | 8.28E-06 |
| chr1 | chr2 | P | 29 | 100 | 205968 | 205996 | 102835 | 102863 | 8.28E-06 |
| chr1 | chr2 | P | 29 | 100 | 337543 | 337571 | 102859 | 102887 | 8.28E-06 |
| chr1 | chr2 | P | 29 | 100 | 415734 | 415762 | 109742 | 109770 | 8.28E-06 |
| chr1 | chr2 | P | 39 | 92.308 | 106874 | 106911 | 109805 | 109842 | 8.28E-06 |
| chr1 | chr2 | P | 45 | 88.889 | 199594 | 199637 | 110984 | 111028 | 8.28E-06 |
| chr1 | chr2 | P | 39 | 92.308 | 106874 | 106911 | 113556 | 113593 | 8.28E-06 |
| chr1 | chr2 | F | 50 | 86 | 305960 | 306009 | 125319 | 125368 | 8.28E-06 |
| chr2 | chr2 | P | 416 | 100 | 59182 | 59597 | 47441 | 47856 | 0 |
| chr2 | chr2 | P | 94 | 100 | 113226 | 113319 | 102949 | 103042 | 1.80E-42 |
| chr2 | chr2 | F | 113 | 94.69 | 113487 | 113599 | 109739 | 109848 | 6.49E-42 |
| chr2 | chr2 | P | 91 | 98.901 | 102922 | 103011 | 1481 | 1571 | 1.40E-38 |
| chr2 | chr2 | P | 95 | 96.842 | 113543 | 113637 | 19148 | 19242 | 5.05E-38 |
| chr2 | chr2 | F | 87 | 98.851 | 77077 | 77163 | 46424 | 46510 | 6.53E-37 |
| chr2 | chr2 | F | 64 | 98.438 | 113257 | 113319 | 1481 | 1544 | 1.43E-23 |
| chr2 | chr2 | F | 82 | 91.463 | 19226 | 19307 | 12619 | 12697 | 5.16E-23 |
| chr2 | chr2 | F | 70 | 94.286 | 13859 | 13928 | 11967 | 12033 | 2.40E-21 |
| chr2 | chr2 | P | 68 | 94.118 | 113572 | 113638 | 109704 | 109770 | 8.63E-21 |
| chr2 | chr2 | P | 57 | 98.246 | 109792 | 109848 | 19186 | 19242 | 3.11E-20 |
| chr2 | chr2 | F | 66 | 93.939 | 57602 | 57667 | 57572 | 57637 | 3.11E-20 |
| chr2 | chr2 | F | 87 | 86.207 | 48214 | 48300 | 48070 | 48156 | 1.44E-18 |
| chr2 | chr2 | F | 143 | 79.72 | 43877 | 44014 | 43655 | 43791 | 5.20E-18 |
| chr2 | chr2 | P | 55 | 96.364 | 109734 | 109788 | 1628 | 1682 | 1.87E-17 |
| chr2 | chr2 | F | 71 | 90.141 | 109702 | 109770 | 19144 | 19213 | 6.72E-17 |
| chr2 | chr2 | P | 49 | 97.959 | 19221 | 19269 | 1711 | 1759 | 8.69E-16 |
| chr2 | chr2 | P | 48 | 97.917 | 113314 | 113361 | 1520 | 1567 | 3.13E-15 |
| chr2 | chr2 | F | 94 | 84.043 | 23824 | 23917 | 23738 | 23823 | 3.13E-15 |
| chr2 | chr2 | F | 48 | 97.917 | 113314 | 113361 | 102926 | 102973 | 3.13E-15 |
| chr2 | chr2 | F | 70 | 87.143 | 36426 | 36495 | 36384 | 36452 | 1.45E-13 |
| chr2 | chr2 | F | 48 | 95.833 | 12820 | 12867 | 12768 | 12814 | 5.23E-13 |
| chr2 | chr2 | F | 53 | 92.453 | 48132 | 48184 | 48096 | 48148 | 5.23E-13 |
| chr2 | chr2 | P | 43 | 97.674 | 28350 | 28392 | 2582 | 2624 | 1.88E-12 |
| chr2 | chr2 | P | 40 | 100 | 109707 | 109746 | 12724 | 12763 | 1.88E-12 |
| chr2 | chr2 | P | 40 | 100 | 2585 | 2624 | 28350 | 28389 | 1.88E-12 |
| chr2 | chr2 | P | 45 | 95.556 | 12618 | 12662 | 1711 | 1755 | 6.77E-12 |
| chr2 | chr2 | P | 55 | 90.909 | 113556 | 113609 | 12806 | 12858 | 2.43E-11 |
| chr2 | chr2 | F | 55 | 90.909 | 19176 | 19229 | 12806 | 12858 | 2.43E-11 |
| chr2 | chr2 | P | 70 | 85.714 | 124100 | 124166 | 95649 | 95718 | 2.43E-11 |
| chr2 | chr2 | F | 36 | 100 | 75445 | 75480 | 23358 | 23393 | 3.15E-10 |
| chr2 | chr2 | F | 42 | 95.238 | 48276 | 48317 | 48240 | 48281 | 3.15E-10 |
| chr2 | chr2 | P | 45 | 93.333 | 109805 | 109848 | 12815 | 12858 | 1.13E-09 |
| chr2 | chr2 | P | 35 | 100 | 113599 | 113633 | 102829 | 102863 | 1.13E-09 |
| chr2 | chr2 | F | 35 | 100 | 120907 | 120941 | 120465 | 120499 | 1.13E-09 |
| chr2 | chr2 | P | 50 | 90 | 113555 | 113603 | 12759 | 12807 | 4.07E-09 |
| chr2 | chr2 | F | 50 | 90 | 19182 | 19230 | 12759 | 12807 | 4.07E-09 |
| chr2 | chr2 | F | 51 | 90.196 | 113589 | 113635 | 12714 | 12763 | 1.47E-08 |
| chr2 | chr2 | P | 52 | 88.462 | 102813 | 102863 | 12727 | 12776 | 5.27E-08 |
| chr2 | chr2 | F | 31 | 100 | 109739 | 109769 | 12759 | 12789 | 1.90E-07 |
| chr2 | chr2 | P | 41 | 92.683 | 109804 | 109843 | 12768 | 12807 | 1.90E-07 |
| chr2 | chr2 | F | 34 | 97.059 | 23502 | 23535 | 23360 | 23393 | 1.90E-07 |
| chr2 | chr2 | F | 44 | 90.909 | 75437 | 75480 | 23493 | 23535 | 1.90E-07 |
| chr2 | chr2 | P | 46 | 89.13 | 109124 | 109169 | 102602 | 102647 | 1.90E-07 |
| chr2 | chr2 | P | 58 | 86.207 | 15360 | 15413 | 15360 | 15413 | 6.82E-07 |
| chr2 | chr2 | F | 35 | 94.286 | 102829 | 102863 | 19152 | 19186 | 2.45E-06 |
| chr2 | chr2 | P | 45 | 88.889 | 60854 | 60898 | 43600 | 43643 | 2.45E-06 |
| chr2 | chr2 | F | 36 | 94.444 | 109709 | 109743 | 102829 | 102863 | 2.45E-06 |
| chr2 | chr2 | F | 48 | 87.5 | 121810 | 121857 | 109010 | 109055 | 2.45E-06 |
| chr2 | chr2 | P | 37 | 91.892 | 19177 | 19213 | 1646 | 1682 | 8.82E-06 |
| chr2 | chr2 | F | 37 | 91.892 | 113572 | 113608 | 1646 | 1682 | 8.82E-06 |
| chr2 | chr2 | P | 31 | 96.774 | 12759 | 12789 | 1647 | 1677 | 8.82E-06 |
| chr2 | chr2 | P | 54 | 85.185 | 28336 | 28388 | 7809 | 7859 | 8.82E-06 |
| chr2 | chr2 | F | 28 | 100 | 112216 | 112243 | 7937 | 7964 | 8.82E-06 |
| chr2 | chr2 | P | 51 | 86.275 | 125323 | 125371 | 94440 | 94488 | 8.82E-06 |
| chr2 | chr2 | P | 31 | 96.774 | 108701 | 108731 | 108701 | 108731 | 8.82E-06 |
| chr2 | chr1 | P | 78 | 97.436 | 113556 | 113633 | 188615 | 188692 | 3.06E-30 |
| chr2 | chr1 | P | 80 | 96.25 | 120224 | 120303 | 28428 | 28507 | 1.10E-29 |
| chr2 | chr1 | P | 81 | 90.123 | 19158 | 19237 | 41558 | 41637 | 2.40E-21 |
| chr2 | chr1 | P | 68 | 94.118 | 113425 | 113492 | 201892 | 201959 | 2.40E-21 |
| chr2 | chr1 | P | 51 | 100 | 109729 | 109779 | 106785 | 106835 | 1.44E-18 |
| chr2 | chr1 | P | 193 | 75.13 | 80873 | 81061 | 228566 | 228756 | 8.69E-16 |
| chr2 | chr1 | P | 57 | 92.982 | 108623 | 108679 | 45262 | 45318 | 3.13E-15 |
| chr2 | chr1 | P | 74 | 87.838 | 110240 | 110313 | 122834 | 122904 | 3.13E-15 |
| chr2 | chr1 | P | 86 | 83.721 | 19191 | 19269 | 263991 | 264076 | 1.88E-12 |
| chr2 | chr1 | P | 40 | 100 | 109707 | 109746 | 313597 | 313636 | 1.88E-12 |
| chr2 | chr1 | P | 65 | 87.692 | 12812 | 12874 | 26258 | 26321 | 6.77E-12 |
| chr2 | chr1 | P | 44 | 95.455 | 1711 | 1754 | 22288 | 22331 | 2.43E-11 |
| chr2 | chr1 | P | 51 | 92.157 | 109777 | 109826 | 64973 | 65021 | 8.76E-11 |
| chr2 | chr1 | P | 49 | 91.837 | 19182 | 19229 | 26274 | 26321 | 3.15E-10 |
| chr2 | chr1 | P | 57 | 87.719 | 37177 | 37233 | 64506 | 64562 | 3.15E-10 |
| chr2 | chr1 | P | 45 | 93.333 | 113572 | 113616 | 68904 | 68948 | 3.15E-10 |
| chr2 | chr1 | P | 42 | 95.238 | 19172 | 19213 | 106794 | 106835 | 3.15E-10 |
| chr2 | chr1 | F | 59 | 88.136 | 12806 | 12862 | 350685 | 350742 | 3.15E-10 |
| chr2 | chr1 | P | 71 | 84.507 | 19170 | 19234 | 243779 | 243848 | 4.07E-09 |
| chr2 | chr1 | P | 57 | 87.719 | 12806 | 12860 | 304313 | 304368 | 4.07E-09 |
| chr2 | chr1 | P | 40 | 95 | 113562 | 113600 | 30504 | 30542 | 1.47E-08 |
| chr2 | chr1 | P | 70 | 82.857 | 80051 | 80119 | 229873 | 229941 | 1.47E-08 |
| chr2 | chr1 | P | 47 | 91.489 | 9327 | 9370 | 313505 | 313551 | 1.47E-08 |
| chr2 | chr1 | P | 39 | 94.872 | 109811 | 109848 | 30505 | 30542 | 5.27E-08 |
| chr2 | chr1 | P | 32 | 100 | 19232 | 19263 | 176590 | 176621 | 5.27E-08 |
| chr2 | chr1 | P | 52 | 88.462 | 109743 | 109791 | 267776 | 267827 | 5.27E-08 |
| chr2 | chr1 | P | 64 | 84.375 | 109729 | 109791 | 356940 | 357000 | 5.27E-08 |
| chr2 | chr1 | P | 57 | 85.965 | 12806 | 12860 | 41475 | 41530 | 1.90E-07 |
| chr2 | chr1 | P | 37 | 94.595 | 60590 | 60626 | 93932 | 93968 | 1.90E-07 |
| chr2 | chr1 | P | 66 | 83.333 | 61736 | 61799 | 237169 | 237232 | 1.90E-07 |
| chr2 | chr1 | P | 52 | 86.538 | 113491 | 113542 | 267776 | 267827 | 1.90E-07 |
| chr2 | chr1 | F | 44 | 90.909 | 9327 | 9369 | 337603 | 337645 | 1.90E-07 |
| chr2 | chr1 | P | 45 | 91.111 | 19191 | 19234 | 415714 | 415756 | 1.90E-07 |
| chr2 | chr1 | P | 43 | 90.698 | 113562 | 113603 | 22428 | 22469 | 6.82E-07 |
| chr2 | chr1 | P | 36 | 94.444 | 1578 | 1613 | 263418 | 263453 | 6.82E-07 |
| chr2 | chr1 | F | 60 | 83.333 | 109790 | 109848 | 41553 | 41609 | 8.82E-06 |
| chr2 | chr1 | P | 32 | 96.875 | 112086 | 112117 | 65131 | 65161 | 8.82E-06 |
| chr2 | chr1 | F | 28 | 100 | 12701 | 12728 | 68882 | 68909 | 8.82E-06 |
| chr2 | chr1 | P | 64 | 81.25 | 40481 | 40544 | 91016 | 91079 | 8.82E-06 |
| chr2 | chr1 | P | 28 | 100 | 109821 | 109848 | 114147 | 114174 | 8.82E-06 |
| chr2 | chr1 | P | 37 | 91.892 | 1646 | 1682 | 188640 | 188676 | 8.82E-06 |
| chr2 | chr1 | P | 34 | 94.118 | 107481 | 107514 | 191012 | 191045 | 8.82E-06 |
| chr2 | chr1 | P | 28 | 100 | 109742 | 109769 | 202142 | 202169 | 8.82E-06 |
| chr2 | chr1 | P | 35 | 94.286 | 12587 | 12621 | 229584 | 229616 | 8.82E-06 |
| chr2 | chr1 | P | 79 | 81.013 | 19232 | 19306 | 258915 | 258985 | 8.82E-06 |
| chr2 | chr1 | F | 58 | 84.483 | 112085 | 112138 | 263354 | 263410 | 8.82E-06 |
| chr2 | chr1 | F | 109 | 75.229 | 70175 | 70283 | 295135 | 295243 | 8.82E-06 |
| chr2 | chr1 | P | 66 | 81.818 | 12795 | 12859 | 337641 | 337704 | 8.82E-06 |
| chr2 | chr1 | P | 52 | 86.538 | 12742 | 12789 | 350592 | 350641 | 8.82E-06 |
| chr2 | chr1 | P | 38 | 92.105 | 12806 | 12842 | 356959 | 356996 | 8.82E-06 |
| chr2 | chr1 | P | 31 | 96.774 | 1647 | 1677 | 368363 | 368393 | 8.82E-06 |

F:Forward dispersed repeats；P: palindromic dispersed repeats

**Supplementary table 7. Tandem repeats in *Magnolia kwangsiensis* mitochondrial genome**

| **NO.** | **Chr** | **Size** | **Copy** | **Repeat sequence** | **Percent Matches** | **Start** | **End** |
| --- | --- | --- | --- | --- | --- | --- | --- |
| 1 | chr1 | 34 | 1.9 | CTCCTATAGGAAGATCCTGCGAAACCTCAACAGG | 93 | 11672 | 11737 |
| 2 | chr1 | 27 | 2.3 | GCATCCCGTCAAAGTTCTTGCTGGAAA | 77 | 23377 | 23437 |
| 3 | chr1 | 19 | 3.9 | ATAAGGCTATTCCATTGAC | 88 | 34936 | 35012 |
| 4 | chr1 | 40 | 2 | GTATAAGGCTATTCCTTTGACATAAGGCTATTCCATTGAC | 100 | 34934 | 35012 |
| 5 | chr1 | 25 | 2 | ATGGAATAATGAGAATGAAATCAAT | 84 | 71051 | 71101 |
| 6 | chr1 | 17 | 1.9 | TGCAGCTTCAAAGCCGA | 100 | 88521 | 88553 |
| 7 | chr1 | 17 | 2.1 | CTCATGTGGCTTACCGG | 94 | 94577 | 94612 |
| 8 | chr1 | 18 | 2.2 | AGGAAGAGAAGAATCAAA | 100 | 175685 | 175724 |
| 9 | chr1 | 14 | 2.3 | TATATATATAGATT | 94 | 180286 | 180317 |
| 10 | chr1 | 20 | 1.9 | CTATAGTAAGTAGAGTAGC | 85 | 195661 | 195698 |
| 11 | chr1 | 22 | 2.1 | CGAGTCACCTTCTTCTAAGCTC | 95 | 206606 | 206651 |
| 12 | chr1 | 13 | 1.9 | ACCACAAACAAAT | 100 | 214340 | 214364 |
| 13 | chr1 | 18 | 2.4 | GACTATGACAGACTCGC | 77 | 227739 | 227781 |
| 14 | chr1 | 18 | 2 | TGATTGGAACATAGCATC | 100 | 237422 | 237457 |
| 15 | chr1 | 19 | 2.4 | GCTATTGGTACTTTCGAAA | 88 | 237861 | 237906 |
| 16 | chr1 | 17 | 2.6 | TAGCTATAAAGAATAAT | 85 | 272708 | 272752 |
| 17 | chr1 | 19 | 1.9 | AGCGAAGCGCATTAATAGG | 94 | 279643 | 279679 |
| 18 | chr1 | 22 | 2 | AGTCAGATCATGAAGGGATAA | 82 | 333343 | 333385 |
| 19 | chr1 | 18 | 2.3 | GCGCTTAAGAGCTTAAGG | 91 | 352875 | 352914 |
| 20 | chr1 | 20 | 2 | CCCCGCCAGCAGGCGGGTAC | 90 | 356755 | 356794 |
| 21 | chr1 | 21 | 2.6 | TTTTCAGCTGGCTCGCTGC | 77 | 357034 | 357086 |
| 22 | chr1 | 21 | 2 | AAGCCAGTAAAAGACAAGTAG | 90 | 379549 | 379590 |
| 23 | chr1 | 30 | 2.1 | TAACTTAAGCAACGGTCGCTAACGCTCCCT | 93 | 389096 | 389158 |
| 24 | chr1 | 16 | 2 | TTATAAGATATAATTG | 100 | 404032 | 404063 |
| 25 | chr1 | 44 | 2 | AATATCATGATCGGGTCGACCAGGTCAGGCCAGATCATGAGTGA | 95 | 405226 | 405314 |
| 26 | chr1 | 5 | 6.4 | TATAG | 96 | 406257 | 406288 |
| 27 | chr1 | 17 | 2 | AACATCATCAATCATGA | 88 | 415368 | 415401 |
| 28 | chr2 | 52 | 2.4 | GTTTTGAAGCTGGCTGTAAAAGCCTATTAGTAAAACGCGCTCATAC | 81 | 12751 | 12864 |
| 29 | chr2 | 42 | 2.6 | TGCTGTTAACGAGAGGAAAACAGTCAAAACGTGACTGTTTGC | 85 | 36384 | 36495 |
| 30 | chr2 | 21 | 2.8 | AAAGAAACGAATGGAAGGATG | 80 | 47819 | 47877 |
| 31 | chr2 | 36 | 2.5 | TTCGTCAGAATCTTATTCAACGAGTTTGATGCAACC | 92 | 48096 | 48184 |
| 32 | chr2 | 24 | 2 | TTCGTCTGAATCTTCTTCTAAGAG | 96 | 48216 | 48264 |
| 33 | chr2 | 36 | 2.2 | TTCGTCTGAATCTTCTTCTAAGAGTCTAGTGCAACC | 93 | 48240 | 48318 |
| 34 | chr2 | 21 | 2.6 | TCTCCTCGTCTGTATTAGGTC | 94 | 53434 | 53488 |
| 35 | chr2 | 30 | 3.2 | ATAGATGTGTCATTATTCTGATTATTGTCC | 93 | 57572 | 57667 |
| 36 | chr2 | 16 | 1.9 | ATAAATAAAAAGAAAG | 93 | 91409 | 91439 |
| 37 | chr2 | 26 | 2.2 | ATAAAGAAATAAAAAGAAAGATAAAG | 83 | 91405 | 91461 |
| 38 | chr2 | 21 | 2 | GACTACTATAGTAGAGTATAGT | 90 | 103484 | 103526 |
| 39 | chr2 | 21 | 2.2 | CCGTCACTAGATCAGGGCGTT | 84 | 124527 | 124572 |

**Supplementary table 8. Homologous analysis in Magnoliaceae**

| #ref | ref len | ref homo len | prop in ref | query | query len | query homo len | prop in query |
| --- | --- | --- | --- | --- | --- | --- | --- |
| Magnolia_kwangsiensis | 555318 | 492680 | 88.72% | Magnolia_biondii | 967100 | 553565 | 57.24% |
| Magnolia_biondii | 967100 | 647298 | 66.93% | Magnolia_officinalis | 930306 | 803307 | 86.35% |
| Magnolia_officinalis | 930306 | 775516 | 83.36% | Magnolia_figo | 773377 | 560587 | 72.49% |
| Magnolia_figo | 773377 | 626623 | 81.02% | Magnolia_liliiflora | 865191 | 666473 | 77.03% |
| Magnolia_liliiflora | 865191 | 436268 | 50.42% | Liriodendron_tulipifera | 551806 | 409879 | 74.28% |

**Supplementary table 9. Chloroplast DNA chloroplast transfer** **in *Magnolia kwangsiensis* mitochondrial genome**

| query-chl | subject-mt | percentage of identical matches | length | number of mismatches | number of gap openings | start of alignment in query | end of alignment in query | start of alignment in subject | end of alignment in subject | expect value | bitscore |
| --- | --- | --- | --- | --- | --- | --- | --- | --- | --- | --- | --- |
| Chloroplast | 1 | 96.936 | 4406 | 53 | 19 | 143096 | 147477 | 397549 | 393202 | 0 | 7313 |
| Chloroplast | 1 | 96.936 | 4406 | 53 | 19 | 100408 | 104789 | 393202 | 397549 | 0 | 7313 |
| Chloroplast | 1 | 91.626 | 2639 | 124 | 50 | 68181 | 70761 | 375544 | 372945 | 0 | 3559 |
| Chloroplast | 1 | 95.2 | 1375 | 37 | 13 | 51088 | 52454 | 387651 | 389004 | 0 | 2146 |
| Chloroplast | 1 | 87.33 | 1618 | 119 | 36 | 35901 | 37471 | 402861 | 404439 | 0 | 1773 |
| Chloroplast | 1 | 80.891 | 1324 | 139 | 49 | 31899 | 33189 | 342648 | 343890 | 0 | 939 |
| Chloroplast | 1 | 97.59 | 498 | 2 | 1 | 147862 | 148359 | 157215 | 156728 | 0 | 845 |
| Chloroplast | 1 | 97.59 | 498 | 2 | 1 | 99526 | 100023 | 156728 | 157215 | 0 | 845 |
| Chloroplast | 1 | 80.656 | 1220 | 124 | 52 | 29563 | 30744 | 340544 | 341689 | 0 | 843 |
| Chloroplast | 1 | 97.297 | 296 | 4 | 3 | 67879 | 68172 | 375893 | 375600 | 3.30E-140 | 499 |
| Chloroplast | 1 | 80.994 | 463 | 51 | 19 | 30848 | 31276 | 342110 | 342569 | 3.54E-90 | 333 |
| Chloroplast | 1 | 73.928 | 886 | 182 | 38 | 142062 | 142925 | 87510 | 86652 | 1.66E-83 | 311 |
| Chloroplast | 1 | 73.928 | 886 | 182 | 38 | 104960 | 105823 | 86652 | 87510 | 1.66E-83 | 311 |
| Chloroplast | 1 | 78.322 | 429 | 65 | 20 | 40705 | 41112 | 316126 | 315705 | 1.02E-65 | 252 |
| Chloroplast | 1 | 99.27 | 137 | 1 | 0 | 142287 | 142423 | 21088 | 20952 | 1.32E-64 | 248 |
| Chloroplast | 1 | 99.27 | 137 | 1 | 0 | 105462 | 105598 | 20952 | 21088 | 1.32E-64 | 248 |
| Chloroplast | 1 | 96.512 | 86 | 3 | 0 | 112875 | 112960 | 246497 | 246412 | 6.41E-33 | 143 |
| Chloroplast | 1 | 96.512 | 86 | 3 | 0 | 134925 | 135010 | 246412 | 246497 | 6.41E-33 | 143 |
| Chloroplast | 1 | 93.506 | 77 | 5 | 0 | 90051 | 90127 | 166602 | 166526 | 1.40E-24 | 115 |
| Chloroplast | 1 | 93.506 | 77 | 5 | 0 | 157758 | 157834 | 166526 | 166602 | 1.40E-24 | 115 |
| Chloroplast | 1 | 92.208 | 77 | 6 | 0 | 55639 | 55715 | 222390 | 222314 | 6.50E-23 | 110 |
| Chloroplast | 1 | 92.208 | 77 | 6 | 0 | 55639 | 55715 | 425317 | 425241 | 6.50E-23 | 110 |
| Chloroplast | 1 | 93.75 | 64 | 4 | 0 | 106110 | 106173 | 279415 | 279352 | 5.06E-19 | 97.1 |
| Chloroplast | 1 | 93.75 | 64 | 4 | 0 | 141712 | 141775 | 279352 | 279415 | 5.06E-19 | 97.1 |
| Chloroplast | 1 | 81.818 | 99 | 15 | 3 | 109045 | 109141 | 229337 | 229240 | 5.10E-14 | 80.5 |
| Chloroplast | 1 | 81.818 | 99 | 15 | 3 | 138744 | 138840 | 229240 | 229337 | 5.10E-14 | 80.5 |
| Chloroplast | 1 | 100 | 31 | 0 | 0 | 33912 | 33942 | 230193 | 230163 | 2.39E-07 | 58.4 |
| Chloroplast | 2 | 99.435 | 2834 | 6 | 2 | 137637 | 140468 | 81506 | 78681 | 0 | 5136 |
| Chloroplast | 2 | 99.435 | 2834 | 6 | 2 | 107417 | 110248 | 78681 | 81506 | 0 | 5136 |
| Chloroplast | 2 | 96.595 | 1204 | 27 | 4 | 83725 | 84914 | 116890 | 115687 | 0 | 1984 |
| Chloroplast | 2 | 84.663 | 163 | 12 | 8 | 96854 | 97015 | 17083 | 16933 | 3.83E-35 | 150 |
| Chloroplast | 2 | 84.663 | 163 | 12 | 8 | 150870 | 151031 | 16933 | 17083 | 3.83E-35 | 150 |

Mt: mitochondrion; cp: chloroplast

**Supplementary table 10. RNA editing type in *Magnolia kwangsiensis* mitochondrial genome**

| Type | RNA-editing | Number | Percentage |
| --- | --- | --- | --- |
| hydrophilic-hydrophilic | CAC (H) => TAC (Y) | 12 |  |
|  | CAT (H) => TAT (Y) | 29 |  |
|  | CGC (R) => TGC (C) | 17 |  |
|  | CGT (R) => TGT (C) | 39 |  |
|  | total | 97 | 11.05% |
| hydrophilic-hydrophobic | ACA (T) => ATA (I) | 12 |  |
|  | ACC (T) => ATC (I) | 7 |  |
|  | ACG (T) => ATG (M) | 14 |  |
|  | ACT (T) => ATT (I) | 10 |  |
|  | CGG (R) => TGG (W) | 43 |  |
|  | TCA (S) => TTA (L) | 109 |  |
|  | TCC (S) => TTC (F) | 60 |  |
|  | TCG (S) => TTG (L) | 70 |  |
|  | TCT (S) => TTT (F) | 89 |  |
|  | total | 414 | 47.15% |
| hydrophilic-stop | CAA (Q) => TAA (X) | 2 |  |
|  | CGA (R) => TGA (X) | 4 |  |
|  | total | 6 | 0.68% |
| hydrophobic-hydrophilic | CCA (P) => TCA (S) | 16 |  |
|  | CCC (P) => TCC (S) | 19 |  |
|  | CCG (P) => TCG (S) | 11 |  |
|  | CCT (P) => TCT (S) | 32 |  |
|  | total | 78 | 8.88% |
| hydrophobic-hydrophobic | CCA (P) => CTA (L) | 67 |  |
|  | CCC (P) => CTC (L) | 19 |  |
|  | CCC (P) => TTC (F) | 10 |  |
|  | CCG (P) => CTG (L) | 50 |  |
|  | CCT (P) => CTT (L) | 38 |  |
|  | CCT (P) => TTT (F) | 16 |  |
|  | CTC (L) => TTC (F) | 17 |  |
|  | CTT (L) => TTT (F) | 36 |  |
|  | GCA (A) => GTA (V) | 10 |  |
|  | GCC (A) => GTC (V) | 3 |  |
|  | GCG (A) => GTG (V) | 10 |  |
|  | GCT (A) => GTT (V) | 7 |  |
|  | total | 283 | 32.23% |

**Supplementary table 11. Ka/Ks value analysis in Magnoliaceae**

| Kaks | Gene | Vs | Kaks | Gene | Species 1 Vs Species 2 |
| --- | --- | --- | --- | --- | --- |
| NA | *atp1* | Magnolia_kwangsiensis vs MK340747.1 | 0.12887 | *nad5* | Magnolia_kwangsiensis vs MK340747.1 |
| NA | *atp1* | Magnolia_kwangsiensis vs NC_049134.1 | 0.325628 | *nad5* | Magnolia_kwangsiensis vs NC_049134.1 |
| NA | *atp1* | Magnolia_kwangsiensis vs NC_064401.1 | 0 | *nad5* | Magnolia_kwangsiensis vs NC_064401.1 |
| NA | *atp1* | Magnolia_kwangsiensis vs NC_082234.1 | 0 | *nad5* | Magnolia_kwangsiensis vs NC_082234.1 |
| NA | *atp1* | Magnolia_kwangsiensis vs NC_085212.1 | 0 | *nad5* | Magnolia_kwangsiensis vs NC_085212.1 |
| 0 | *atp4* | Magnolia_kwangsiensis vs MK340747.1 | 0.263993 | *nad6* | Magnolia_kwangsiensis vs MK340747.1 |
| NA | *atp4* | Magnolia_kwangsiensis vs NC_049134.1 | 0.262238 | *nad6* | Magnolia_kwangsiensis vs NC_049134.1 |
| NA | *atp4* | Magnolia_kwangsiensis vs NC_064401.1 | NA | *nad6* | Magnolia_kwangsiensis vs NC_064401.1 |
| NA | *atp4* | Magnolia_kwangsiensis vs NC_082234.1 | NA | *nad6* | Magnolia_kwangsiensis vs NC_082234.1 |
| NA | *atp4* | Magnolia_kwangsiensis vs NC_085212.1 | NA | *nad6* | Magnolia_kwangsiensis vs NC_085212.1 |
| NA | *atp6* | Magnolia_kwangsiensis vs MK340747.1 | NA | *nad7* | Magnolia_kwangsiensis vs MK340747.1 |
| NA | *atp6* | Magnolia_kwangsiensis vs NC_049134.1 | NA | *nad7* | Magnolia_kwangsiensis vs NC_049134.1 |
| NA | *atp6* | Magnolia_kwangsiensis vs NC_064401.1 | NA | *nad7* | Magnolia_kwangsiensis vs NC_064401.1 |
| NA | *atp6* | Magnolia_kwangsiensis vs NC_082234.1 | NA | *nad7* | Magnolia_kwangsiensis vs NC_082234.1 |
| NA | *atp6* | Magnolia_kwangsiensis vs NC_085212.1 | NA | *nad7* | Magnolia_kwangsiensis vs NC_085212.1 |
| NA | *atp8* | Magnolia_kwangsiensis vs MK340747.1 | 0 | *nad9* | Magnolia_kwangsiensis vs MK340747.1 |
| NA | *atp8* | Magnolia_kwangsiensis vs NC_049134.1 | 0 | *nad9* | Magnolia_kwangsiensis vs NC_049134.1 |
| NA | *atp8* | Magnolia_kwangsiensis vs NC_064401.1 | NA | *nad9* | Magnolia_kwangsiensis vs NC_064401.1 |
| NA | *atp8* | Magnolia_kwangsiensis vs NC_082234.1 | NA | *nad9* | Magnolia_kwangsiensis vs NC_082234.1 |
| NA | *atp8* | Magnolia_kwangsiensis vs NC_085212.1 | 0 | *nad9* | Magnolia_kwangsiensis vs NC_085212.1 |
| NA | *atp9* | Magnolia_kwangsiensis vs MK340747.1 | NA | *rpl10* | Magnolia_kwangsiensis vs MK340747.1 |
| 0 | *atp9* | Magnolia_kwangsiensis vs NC_049134.1 | 0.545563 | *rpl10* | Magnolia_kwangsiensis vs NC_049134.1 |
| NA | *atp9* | Magnolia_kwangsiensis vs NC_064401.1 | NA | *rpl10* | Magnolia_kwangsiensis vs NC_064401.1 |
| NA | *atp9* | Magnolia_kwangsiensis vs NC_082234.1 | NA | *rpl10* | Magnolia_kwangsiensis vs NC_082234.1 |
| 0 | *atp9* | Magnolia_kwangsiensis vs NC_085212.1 | NA | *rpl10* | Magnolia_kwangsiensis vs NC_085212.1 |
| NA | *ccmB* | Magnolia_kwangsiensis vs MK340747.1 | 0.687758 | *rpl2* | Magnolia_kwangsiensis vs MK340747.1 |
| NA | *ccmB* | Magnolia_kwangsiensis vs NC_049134.1 | 0.339389 | *rpl2* | Magnolia_kwangsiensis vs NC_049134.1 |
| NA | *ccmB* | Magnolia_kwangsiensis vs NC_064401.1 | 0.217628 | *rpl2* | Magnolia_kwangsiensis vs NC_064401.1 |
| NA | *ccmB* | Magnolia_kwangsiensis vs NC_082234.1 | 0.339389 | *rpl2* | Magnolia_kwangsiensis vs NC_082234.1 |
| NA | *ccmB* | Magnolia_kwangsiensis vs NC_085212.1 | 0.339389 | *rpl2* | Magnolia_kwangsiensis vs NC_085212.1 |
| 0.485049 | *ccmC* | Magnolia_kwangsiensis vs MK340747.1 | 0.305338 | *rpl5* | Magnolia_kwangsiensis vs MK340747.1 |
| 1.37927 | *ccmC* | Magnolia_kwangsiensis vs NC_049134.1 | 0.223745 | *rpl5* | Magnolia_kwangsiensis vs NC_049134.1 |
| NA | *ccmC* | Magnolia_kwangsiensis vs NC_064401.1 | NA | *rpl5* | Magnolia_kwangsiensis vs NC_064401.1 |
| NA | *ccmC* | Magnolia_kwangsiensis vs NC_082234.1 | 0.301398 | *rpl5* | Magnolia_kwangsiensis vs NC_082234.1 |
| NA | *ccmC* | Magnolia_kwangsiensis vs NC_085212.1 | 0.223745 | *rpl5* | Magnolia_kwangsiensis vs NC_085212.1 |
| 0.251298 | *ccmFc* | Magnolia_kwangsiensis vs MK340747.1 | 0.243591 | *rps1* | Magnolia_kwangsiensis vs MK340747.1 |
| 0.125513 | *ccmFc* | Magnolia_kwangsiensis vs NC_049134.1 | NA | *rps1* | Magnolia_kwangsiensis vs NC_049134.1 |
| 0.252222 | *ccmFc* | Magnolia_kwangsiensis vs NC_064401.1 | NA | *rps1* | Magnolia_kwangsiensis vs NC_064401.1 |
| 0.252222 | *ccmFc* | Magnolia_kwangsiensis vs NC_082234.1 | NA | *rps1* | Magnolia_kwangsiensis vs NC_082234.1 |
| 0.252222 | *ccmFc* | Magnolia_kwangsiensis vs NC_085212.1 | NA | *rps1* | Magnolia_kwangsiensis vs NC_085212.1 |
| 1.24985 | *ccmFn* | Magnolia_kwangsiensis vs MK340747.1 | 0.226617 | *rps10* | Magnolia_kwangsiensis vs MK340747.1 |
| 0.722504 | *ccmFn* | Magnolia_kwangsiensis vs NC_049134.1 | NA | *rps10* | Magnolia_kwangsiensis vs NC_049134.1 |
| NA | *ccmFn* | Magnolia_kwangsiensis vs NC_064401.1 | NA | *rps10* | Magnolia_kwangsiensis vs NC_064401.1 |
| NA | *ccmFn* | Magnolia_kwangsiensis vs NC_082234.1 | NA | *rps10* | Magnolia_kwangsiensis vs NC_082234.1 |
| NA | *ccmFn* | Magnolia_kwangsiensis vs NC_085212.1 | NA | *rps10* | Magnolia_kwangsiensis vs NC_085212.1 |
| 0 | *cob* | Magnolia_kwangsiensis vs MK340747.1 | 0.309265 | *rps11* | Magnolia_kwangsiensis vs MK340747.1 |
| NA | *cob* | Magnolia_kwangsiensis vs NC_049134.1 | NA | *rps11* | Magnolia_kwangsiensis vs NC_049134.1 |
| 0 | *cob* | Magnolia_kwangsiensis vs NC_064401.1 | NA | *rps11* | Magnolia_kwangsiensis vs NC_064401.1 |
| NA | *cob* | Magnolia_kwangsiensis vs NC_082234.1 | NA | *rps11* | Magnolia_kwangsiensis vs NC_082234.1 |
| NA | *cob* | Magnolia_kwangsiensis vs NC_085212.1 | NA | *rps11* | Magnolia_kwangsiensis vs NC_085212.1 |
| NA | *cox1* | Magnolia_kwangsiensis vs MK340747.1 | NA | *rps12* | Magnolia_kwangsiensis vs MK340747.1 |
| 0.268236 | *cox1* | Magnolia_kwangsiensis vs NC_049134.1 | NA | *rps12* | Magnolia_kwangsiensis vs NC_049134.1 |
| NA | *cox1* | Magnolia_kwangsiensis vs NC_064401.1 | NA | *rps12* | Magnolia_kwangsiensis vs NC_064401.1 |
| 0.328847 | *cox1* | Magnolia_kwangsiensis vs NC_082234.1 | NA | *rps12* | Magnolia_kwangsiensis vs NC_082234.1 |
| NA | *cox1* | Magnolia_kwangsiensis vs NC_085212.1 | NA | *rps12* | Magnolia_kwangsiensis vs NC_085212.1 |
| 0.183601 | *cox2* | Magnolia_kwangsiensis vs MK340747.1 | NA | *rps13* | Magnolia_kwangsiensis vs MK340747.1 |
| NA | *cox2* | Magnolia_kwangsiensis vs NC_049134.1 | NA | *rps13* | Magnolia_kwangsiensis vs NC_049134.1 |
| NA | *cox2* | Magnolia_kwangsiensis vs NC_064401.1 | NA | *rps13* | Magnolia_kwangsiensis vs NC_064401.1 |
| NA | *cox2* | Magnolia_kwangsiensis vs NC_082234.1 | NA | *rps13* | Magnolia_kwangsiensis vs NC_082234.1 |
| NA | *cox2* | Magnolia_kwangsiensis vs NC_085212.1 | NA | *rps13* | Magnolia_kwangsiensis vs NC_085212.1 |
| NA | *cox3* | Magnolia_kwangsiensis vs MK340747.1 | NA | *rps14* | Magnolia_kwangsiensis vs MK340747.1 |
| NA | *cox3* | Magnolia_kwangsiensis vs NC_049134.1 | NA | *rps14* | Magnolia_kwangsiensis vs NC_049134.1 |
| NA | *cox3* | Magnolia_kwangsiensis vs NC_064401.1 | NA | *rps14* | Magnolia_kwangsiensis vs NC_064401.1 |
| NA | *cox3* | Magnolia_kwangsiensis vs NC_082234.1 | NA | *rps14* | Magnolia_kwangsiensis vs NC_082234.1 |
| NA | *cox3* | Magnolia_kwangsiensis vs NC_085212.1 | NA | *rps14* | Magnolia_kwangsiensis vs NC_085212.1 |
| 1.96261 | *matR* | Magnolia_kwangsiensis vs MK340747.1 | NA | *rps19* | Magnolia_kwangsiensis vs MK340747.1 |
| 0 | *matR* | Magnolia_kwangsiensis vs NC_049134.1 | NA | *rps19* | Magnolia_kwangsiensis vs NC_049134.1 |
| NA | *matR* | Magnolia_kwangsiensis vs NC_064401.1 | NA | *rps19* | Magnolia_kwangsiensis vs NC_064401.1 |
| 0 | *matR* | Magnolia_kwangsiensis vs NC_082234.1 | NA | *rps19* | Magnolia_kwangsiensis vs NC_082234.1 |
| 0 | *matR* | Magnolia_kwangsiensis vs NC_085212.1 | NA | *rps19* | Magnolia_kwangsiensis vs NC_085212.1 |
| 0 | *mttB* | Magnolia_kwangsiensis vs MK340747.1 | NA | *rps2* | Magnolia_kwangsiensis vs MK340747.1 |
| NA | *mttB* | Magnolia_kwangsiensis vs NC_049134.1 | 0.505267 | *rps2* | Magnolia_kwangsiensis vs NC_049134.1 |
| NA | *mttB* | Magnolia_kwangsiensis vs NC_064401.1 | NA | *rps2* | Magnolia_kwangsiensis vs NC_064401.1 |
| NA | *mttB* | Magnolia_kwangsiensis vs NC_082234.1 | NA | *rps2* | Magnolia_kwangsiensis vs NC_082234.1 |
| NA | *mttB* | Magnolia_kwangsiensis vs NC_085212.1 | NA | *rps2* | Magnolia_kwangsiensis vs NC_085212.1 |
| 0.895272 | *nad1* | Magnolia_kwangsiensis vs MK340747.1 | NA | *rps4* | Magnolia_kwangsiensis vs MK340747.1 |
| NA | *nad1* | Magnolia_kwangsiensis vs NC_049134.1 | 0 | *rps4* | Magnolia_kwangsiensis vs NC_049134.1 |
| NA | *nad1* | Magnolia_kwangsiensis vs NC_064401.1 | NA | *rps4* | Magnolia_kwangsiensis vs NC_064401.1 |
| NA | *nad1* | Magnolia_kwangsiensis vs NC_082234.1 | NA | *rps4* | Magnolia_kwangsiensis vs NC_082234.1 |
| NA | *nad1* | Magnolia_kwangsiensis vs NC_085212.1 | 0 | *rps4* | Magnolia_kwangsiensis vs NC_085212.1 |
| 3.02648 | *nad2* | Magnolia_kwangsiensis vs MK340747.1 | NA | *rps7* | Magnolia_kwangsiensis vs MK340747.1 |
| NA | *nad2* | Magnolia_kwangsiensis vs NC_049134.1 | 0.95379 | *rps7* | Magnolia_kwangsiensis vs NC_049134.1 |
| NA | *nad2* | Magnolia_kwangsiensis vs NC_064401.1 | NA | *rps7* | Magnolia_kwangsiensis vs NC_064401.1 |
| NA | *nad2* | Magnolia_kwangsiensis vs NC_082234.1 | 1.3518 | *rps7* | Magnolia_kwangsiensis vs NC_082234.1 |
| NA | *nad2* | Magnolia_kwangsiensis vs NC_085212.1 | 1.3518 | *rps7* | Magnolia_kwangsiensis vs NC_085212.1 |
| NA | *nad3* | Magnolia_kwangsiensis vs MK340747.1 | 0.314128 | *sdh3* | Magnolia_kwangsiensis vs MK340747.1 |
| NA | *nad3* | Magnolia_kwangsiensis vs NC_049134.1 | NA | *sdh3* | Magnolia_kwangsiensis vs NC_049134.1 |
| NA | *nad3* | Magnolia_kwangsiensis vs NC_064401.1 | NA | *sdh3* | Magnolia_kwangsiensis vs NC_064401.1 |
| NA | *nad3* | Magnolia_kwangsiensis vs NC_082234.1 | NA | *sdh3* | Magnolia_kwangsiensis vs NC_082234.1 |
| NA | *nad3* | Magnolia_kwangsiensis vs NC_085212.1 | NA | *sdh3* | Magnolia_kwangsiensis vs NC_085212.1 |
| 0.748716 | *nad4* | Magnolia_kwangsiensis vs MK340747.1 | 0 | *sdh4* | Magnolia_kwangsiensis vs MK340747.1 |
| 0.599757 | *nad4* | Magnolia_kwangsiensis vs NC_049134.1 | NA | *sdh4* | Magnolia_kwangsiensis vs NC_049134.1 |
| NA | *nad4* | Magnolia_kwangsiensis vs NC_064401.1 | NA | *sdh4* | Magnolia_kwangsiensis vs NC_064401.1 |
| 0.518854 | *nad4* | Magnolia_kwangsiensis vs NC_082234.1 | NA | *sdh4* | Magnolia_kwangsiensis vs NC_082234.1 |
| 0.22534 | *nad4* | Magnolia_kwangsiensis vs NC_085212.1 | NA | *sdh4* | Magnolia_kwangsiensis vs NC_085212.1 |
| NA | *nad4L* | Magnolia_kwangsiensis vs MK340747.1 |  |  |  |
| 0 | *nad4L* | Magnolia_kwangsiensis vs NC_049134.1 |  |  |  |
| NA | *nad4L* | Magnolia_kwangsiensis vs NC_064401.1 |  |  |  |
| NA | *nad4L* | Magnolia_kwangsiensis vs NC_082234.1 |  |  |  |
| 0 | *nad4L* | Magnolia_kwangsiensis vs NC_085212.1 |  |  |  |
